# A database of general knowledge question performance in older adults

**DOI:** 10.3758/s13428-020-01493-2

**Published:** 2021-01-14

**Authors:** Jennifer H. Coane, Sharda Umanath

**Affiliations:** 1 Department of Psychology, Colby College, Waterville, Maine 04901 USA; 2 Claremont McKenna College, Claremont, CA USA

**Keywords:** Cognitive aging, General knowledge, Retrieval failures, Recall, Multiple-choice testing

## Abstract

General knowledge questions are used across a variety of research and clinical settings to measure cognitive processes such as metacognition, knowledge acquisition, retrieval processes, and intelligence. Existing norms only report performance in younger adults, rendering them of limited utility for cognitive aging research because of well-documented differences in semantic memory and knowledge as a function of age. Specifically, older adults typically outperform younger adults in tasks assessing retrieval of information from the knowledge base. Here we present older adult performance on 421 general knowledge questions across a range of difficulty levels. Cued recall data, including data on the phenomenology of retrieval failures, and multiple-choice data are available. These norms will allow researchers to identify questions that are not likely to be known by older adult participants to examine learning or acquisition processes, or to select questions within a range of marginal accessibility, for example. Comparisons with young adult data from prior databases confirms previous findings of greater knowledge in older adults and indicates there is preservation of knowledge from early adulthood into older adulthood.

**Supplementary Information:**

The online version contains supplementary material available at 10.3758/s13428-020-01493-2.

A standard categorization of long-term, declarative memory is as either episodic or semantic (Tulving, 1972). Many, if not most, empirical studies of memory focus on the nature of episodic (Tulving, 1983) or event (Rubin & Umanath, 2015) memory. Since the publication of Ebbinghaus’ (1885/1913) ground-breaking work on the nature of forgetting and retention, a wealth of research has examined the processes by which we learn, remember, and forget information. A key element of Ebbinghaus’ empirical approach was to study nonsense syllables – information devoid of pre-existing meaning and therefore removing the influence of prior knowledge – to obtain a relatively “pure” measure of retention. In the decades following, a substantial amount of research in memory labs around the world has relied on simple stimuli, such as images, words, or syllables. Thus, much of this research has focused on memory for specific episodes or events over short retention intervals, mostly within minutes or days (see Bahrick, Hall, & Baker, 2013).

In contrast, the nature of semantic memory or the knowledge base is such that assessing the contents of this system can be challenging. The assumption is that the contents include pre-experimentally acquired information that is relatively stable over the lifespan and can be accessed across contexts (Tulving, 1972, 1985). In this view, general knowledge (GK) is defined as culturally relevant information that is shared by individuals living within a specific social environment. This knowledge can be acquired through formal education or through exposure to media (e.g., news, radio and television programming, books, magazines, Internet) either intentionally or incidentally (Irwing, Cammock, & Lynn, 2001). There is a vast quantity of information stored in the knowledge base, and it is accessed or retrieved with speed and relative efficiency and accuracy. Defining, and thus studying, this body of knowledge presents a set of specific challenges. As the term “general” implies, GK should be broadly shared across individuals within the same cultural milieu.

Interestingly, GK has been found to predict recent and current event knowledge (Beier & Ackerman, 2001), and Ackerman, Bowen, Beier, and Kanfer (2001) note that there are individual differences in GK that can influence the overall relationship between knowledge (crystallized intelligence) and fluid intelligence. Furthermore, GK is important in text comprehension and memory because it provides access to organizational structures (e.g., Bransford & Johnson, 1972). In the context of memory, prior knowledge has powerful effects on the execution of episodic memory tasks (e.g., false memory paradigms, Roediger & McDermott, 1995; schema-based remembering, Bartlett, 1932; long-term working memory, Ericsson & Kintsch, 1995). It is also important to note that GK varies with demographic variables, such as age and gender (Furnham & Chamorro-Premuzic, 2006).

GK questions are one tool used to probe the contents of long-term memory that are not dependent on a prior specific encoding event. GK questions have been used across a variety of tasks and domains, from research on metacognition and the phenomenology of memory (e.g., Coane & Umanath, 2019; Marquié & Huet, 2000; Morson, Moulin, & Souchay, 2015; Singer & Tiede, 2008; Tullis, 2018), long-term memory (e.g., Berger, Hall, & Bahrick, 1999; Cantor, Eslick, Marsh, Bjork, & Bjork, 2015; Marsh, Meade, & Roediger, 2003; McIntyre & Craik, 1987; Sitzman, Rhodes, & Tauber, 2014), the role of curiosity in learning (e.g., Kang et al., 2009; McGillivray, Murayama, & Castel, 2015; Wade & Kidd, 2019), educational applications (e.g., Arnold, Graham, & Hollingsworth-Hughes, 2017), tip-of-the tongue (TOT) states (e.g., Brown, 1991; Burke, MacKay, Worthley, & Wade, 1991), and age-related changes in cognitive function (e.g., Dodson, Bawa, & Krueger, 2007; Marsh, Balota, & Roediger, 2005; Sitzman, Rhodes, Tauber, & Liceraide, 2015; see Umanath & Marsh, 2014, for a review). GK questions are also often included in intelligence tests (e.g., Wechsler, Stanford-Binet), regardless of attempts to make tests “culture-neutral” and are elements of many cognitive batteries that assess cognitive functioning in older adults or patient populations (e.g., Stone, Dodrill, & Johnson, 2001).

Given this extensive use of GK questions in research and clinical settings, having normative data on a large set of items is important. In 1980, Nelson and Narens published a database of 300 GK questions, in which they provided recall accuracy, recall latency, and feeling-of-knowing (FOK; Hart, 1965) ratings. More recently, Tauber, Dunlosky, Rawson, Rhodes, and Sitzman (2013) revised and updated these norms, noting some important changes in accessibility of information over the intervening three decades. Whereas some items in the norms showed relative stability over time, others did not, thus emphasizing the importance of having cohort-specific GK norms. However, both Nelson and Narens’ and Tauber et al.’s norms were obtained from only younger adult participants, thus raising the question of whether these norms are equally valid for older adult samples. To give one specific example from our own work, Coane and Umanath (2019), using GK items from Cantor et al. (2015) that yielded approximately 35% accuracy in younger adults, found accuracy rates over 60% in older adults.

One of the most robust findings in cognitive aging is that the knowledge base/semantic memory/crystallized intelligence increases over the lifespan and is maintained into very old age (e.g., Dixon, 2003; Park, 2000; Salthouse, 2004; Spreng & Turner, 2019; Umanath & Marsh, 2014). Older adults frequently outperform younger adults on tests of vocabulary (Arbuckle, Cooney, Milne, & Melchior, 1994; Bahrick, 1984; Mitchell, 1989; Perlmutter, 1978) and other forms of crystallized intelligence (Brod, Werkle-Bergner, & Shing, 2013; Cornelius & Caspi, 1987; Staudinger, Cornelius, & Baltes, 1989). In some cases, it can be hard to isolate age-related changes in cognitive processes because of the vast reserve OAs have in terms of prior knowledge. Indeed, OAs can be considered knowledge experts (Hoyer, Rybash, & Roodin, 1989; Perlmutter, 1978), with vast, highly organized knowledge bases (for a review, see Umanath & Marsh, 2014).

However, retrieval struggles increase in old age (e.g., Burke et al., 1991; Cavanaugh, Grady, & Perlmutter, 1983), as manifested by higher memory complaints and more frequent tip-of-the-tongue (TOT) states. Thus, although OAs have greater knowledge than YAs, this knowledge is not always accessible. Marginal knowledge is defined operationally by inconsistent retrieval success. Typically, participants answer a series of GK questions; after initially being unable to produce a correct answer (retrieval failure), participants often then select it from a set of options, demonstrating its availability in memory (Berger et al., 1999; Cantor et al., 2015; see Umanath, 2016, for another operationalization). Other evidence for the fluctuation in access to knowledge is revealed by the finding that OAs still show spreading activation in priming and memory tasks (e.g., Balota et al., 1999), which reflects the availability of related information in memory, but are often slower to respond and sometimes struggle to retrieve their knowledge (e.g., Brod et al., 2013; Burke & Shafto, 2004). This demonstrates unstable access to the knowledge base (Umanath, 2016).

Thus, age-specific norms are important for a number of reasons. First, appropriate norms can avoid under- or over-estimating knowledge. Second, knowledge can affect performance in a number of other tasks/situations (e.g., language comprehension, episodic memory), so having an accurate assessment of what someone knows is important, to control for differential effects of prior knowledge. For example, researchers examining marginal knowledge (Berger et al., 1999; Cantor et al., 2015) or illusory truth (e.g., Fazio, Brashier, Payne, & Marsh, 2015) can more effectively identify items that are likely to elicit the desired level of familiarity or accessibility. Researchers examining TOTs can also benefit by having access to a large pool of GK questions, which would allow them to predict with greater accuracy what items might elicit a TOT state, thereby increasing the number of potential observations. Third, age-appropriate norms allow researchers to examine different groups of participants controlling for overall level of performance. For example, using norms, researchers can select different items for OA and YA to match on levels of difficulty to minimize effects of baseline differences. Fourth, in studies in which learning of information is a direct measure, finding material that is not already known to the participants is essential for avoiding ceiling effects and isolating the influence of manipulated variables.

In some cases, researchers have used different materials for younger and older adults (e.g., Mutter, Lindsey, & Pliske, 1995; Pliske & Mutter, 1996) to account for differences in baseline knowledge levels. This generally requires that researchers do extensive piloting of materials to select items that are equally difficult or easy for participants of different ages. Considering some of the challenges inherent in aging research (e.g., limited number of participants in a pool, costs associated with compensation), this can become an obstacle to researchers, especially those working in less urban areas or with limited access to funding.

Here, we present a database consisting of 421 GK questions that have been normed in cued-recall and multiple-choice testing using older adult participants. The questions ranged in difficulty and came from a variety of sources. A subset of the questions was selected from the Nelson and Narens (1980) and Tauber et al. (2013) norms, thereby allowing us to examine potential cohort differences between younger and older adult participants’ knowledge by comparing our sample to Tauber et al. This comparison broadly addresses the question as to whether certain items are similarly accessible at this particular historical context (i.e., 30–40 years after the original norms were gathered), regardless of age. For example, as Tauber et al. noted, some items in the original norms were less known to college-aged participants around 2013 than to participants in the late 1970s/early 1980s (such as the name of the Lone Ranger’s sidekick), whereas others were more known to the former group (e.g., the capital of Iraq). By comparing older adults today to the participants in the original Nelson and Narens’ norms, who are, on average, in their 60s and 70s now, we can begin to examine the preservation of knowledge over time. It is possible that older adults might show preserved knowledge of information that was relevant to them or more commonly present in popular media when they were younger (as is commonly found in autobiographical memory, where the reminiscence bump refers to better memory for events occurring in one’s teens and 20s; Berntsen & Rubin, 2004; Rathbone, Moulin, & Conway, 2008). Given that Tauber et al. noted significant changes in the knowledge base over time in college students, a cross-sample comparison might provide some insights into whether general knowledge within a cohort changes in the same way as it does across cohorts.^1^

A body of research has examined cohort effects in another measure of crystallized intelligence: Vocabulary. Older adults typically outperform younger adults in these measures, a finding that has been attributed to a number of factors, among them differences in education levels (older adult samples generally have more years of education than the first- and second-year students who participate in research studies), to item selection effects (a commonly used vocabulary task, Shipley, was developed in 1940), to changes in reading habits among younger cohorts (see Verhaeghen, 2003, for a discussion). Similarly, recent research on category norms reported changes in category dominance and exemplar generation across cohorts of younger adults in earlier norm studies and older adults (Castro, Curley, & Hertzog, 2020). Thus, existing research on cohort effects in different measures of knowledge suggest that performance is likely to change over time.

In the two studies reported here, we examined performance on both open-ended questions (cued-recall) and multiple-choice questions. The former typically require more effortful search strategies in memory, whereas the latter, because the answer is provided, are more sensitive to discrimination among related foils. Older adults, in episodic tasks, generally show more marked deficits in tests that offer less environmental support, such as cued-recall, than tests such as recognition (Craik & Byrd, 1982; see Balota, Dolan, & Duchek, 2000, for a review).

## Experiment 1

A total of 421 questions, ranging in difficulty and selected from a variety of sources (see Materials for more details), were normed in a cued-recall test. The questions were divided into four sets ranging from 70 to 148 questions each. For each question, participants had the option of providing an answer, indicating they could not remember (DR), or indicating they did not know (DK) the answer. Specific guidelines on when and how to use DR and DK were not provided (Coane & Umanath, 2019). Participants were recruited from an online platform or tested in the laboratory to provide access to different populations.

### Method

#### Participants

Responses to the open-ended questions were obtained from laboratory studies and online sources. Two sets of data were collected online using Amazon’s Mechanical Turk (MTurk) platform (Mason & Suri, 2012), and the other two came from experimental studies (Coane & Umanath, 2019; Umanath, Coane, & Walsh, n.d.). See Table 1 for demographic information for all participants.

**Table 1:** Demographic information for participants in Experiment 1

	*N*	Age (SD)	Education (SD)	*N* women (%)	Shipley vocabulary (SD)	MMSE (SD)
Set A	57	62.7 (5.05)*	15.05 (2.43)*	31 (54)	N/A	N/A
Set B	55	67.76 (5.30)	15.83 (2.95)	31 (57)	N/A	N/A
Set C	67	68.4 (6.45)	16.36 (2.79)	49 (73)	35.4 (3.88)	29.57 (.63)*
Set D	66	74.18 (7.12)	16.70 (2.28)	50 (76)	35.92 (2.82)	28.58 (1.34)^

*Due to programming errors, exact age and years of education are available for 37 participants in Set A

**MMSE scores were only available for 28 participants. Scores ranged from 28 to 30

^One participant was missing an MMSE score. Scores ranged from 24 to 30

For the two groups recruited online, we set the following requirements on MTurk, using the platform’s pre-screening qualifications: Participants had to be over age 55, be in possession of a high school diploma, have a US IP address, and have a 95% approval rate. Participants were only allowed to participate in one task (i.e., we filtered all HITs [jobs available to MTurk workers are called HITs] after the first batch to exclude previous participants). Fifty-seven participants completed the first set of questions (Set A; see Table 1 for demographic information). Due to a programming error, detailed demographic data are only available for 37 participants (the first batch of data collection only requested age range and categorical responses for education levels). Of the remaining 20 participants for whom specific information is not available, eight reported their age between 51 and 60 and 12 reported their age between 61 and 80. In terms of education, the 20 participants for whom we did not have exact years of education reported the following: three high school diploma, 11 some college/college graduate, six some graduate training/graduate degree. All reported being native speakers of English.

The second group of participants (Set B) consisted of 55 older adults recruited on MTurk (see Table 1). One participant reported being 48; their data were omitted from the analyses. All participants were native speakers of English.

Participants tested in the lab were community-dwelling older adults (ages 60+). For the Set C questions, the participants were 67 older adults recruited from the Waterville, Maine, community who participated in two experimental studies examining the phenomenology of retrieval failures (see Coane & Umanath, 2019. All but two participants reported English as their native language.^2^ The final participant group (Set D) were 66 older adults tested at Colby College (*n* = 32) and at Claremont McKenna College (*n* = 34; Umanath et al, n.d). Five participants reported English was not their first language (see Table 1 for demographic information).

Overall, the online samples were slightly younger, in part due to the fact that the default age qualification in MTurk is “55 and older,” whereas participants in the lab are recruited at age 60 and older. Online samples also had approximately 1 year less education than the samples tested in the laboratory.

#### Materials

As mentioned above, four different sets of questions were used. Two sets (A and B) were developed for the purposes of gathering the present normative data; the other two (C and D) were originally used in experimental tasks in our labs. The encoding phase of the experimental tasks was similar to the norming task, in that participants provided responses to open-ended questions about a variety of topics or responded DR or DK. The questions in all sets covered a variety of topics, ranging from literature to sports, geography, history, science and technology, pop culture, and music (see the Appendix and the online supplement [http://web.colby.edu/memoryandlanguagelab/publications/stimuli-and-data-sets/] for the full set of items).

Set A consisted of 148 questions selected from two online sources, GitHub (https://github.com/el-cms/Open-trivia-database) and the online version of the Encyclopedia Britannica, which includes an online quiz platform (www.britannica.com/quiz). Set B included 134 questions from Burke et al. (1991), in which the main objective was to study tip of the tongue states, and Wang, Brashier, Wing, Marsh, and Cabeza (2016), in which the authors examined illusory truth effects. Seven items were omitted from analyses because they were accidentally excluded from the multiple-choice version of the task (see Experiment 2), leaving 127 items in the analyses. Set C included 84 questions from Cantor et al. (2015), in which the main objective was to study marginal knowledge. Items in this set had a mean difficulty of .39 (range .2 to .68) in younger adults (as reported in Cantor et al., Experiment 1). Set D included 70 questions from the Nelson and Narens (1980) and Tauber et al. (2013) norms.^3^ The mean accuracy of these items was .12 (SD = .08) in the Nelson and Narens norms and .02 (SD = .02) in the Tauber et al. norms (these items were selected for a different experiment [Umanath et al., n.d.] with the goal of eliciting more DR and DK responses in older adults). The analyses below only include 62 items from Set D because eight questions were also included in Set B, and we wanted to avoid having a small set of stimuli over-sampled. The sets used in the online testing also included two bot check questions (participants were asked to enter a specific response). See Appendix A for a full set of stimuli as well as item-level response information.

#### Procedure

Participants recruited through MTurk completed the task at a time and location of their choosing. The task was programmed using Gorilla software (gorilla.sc; Anwyl-Irvine, Massonnié, Flitton, Kirkham & Evershed, 2019). After providing consent and basic demographic information, participants were presented the questions, one at a time in random order. They were informed that they should answer by typing on the computer’s external keyboard or they could indicate “don’t remember”/DR or “don’t know”/DK. The exact instructions presented to participants were:

“Some of the questions may be quite difficult. Please do your best to answer them. Do

not use the Internet or other resources to look up the answer. Please do not leave any

questions blank. We are just interested in learning what people know/do not know. Most

questions require only a one- or two-word response. If you do not know the answer, please type DK ("Don't Know"). If you do not remember the answer, please type DR ("Don't Remember"). Please do not leave any questions blank.”

Importantly, no additional instructions were provided on when and how to use these options. They were further told some of the general knowledge questions were quite difficult and were informed that the purpose of the study was simply to assess what people know. Because MTurk participants are paid by the number of jobs they complete online, we were reasonably confident participants would not spend additional time searching for correct answers online.^4^ No additional measures of cognitive performance were obtained from the online samples.

Participants who were tested in the lab were tested individually. The study was programmed using E-Prime (Schneider, Eschman, & Zuccolotto, 2012). After providing consent and basic demographic information, participants were given instructions similar to those provided to the online sample.^5^ Participants tested in the lab were compensated at a rate of $10/hr; MTurk participants were compensated $5 (the task was completed on average in 50 min).

### Results and Discussion

Analyses are reported at the item level. As noted above, for each item between 54 and 67 participants responded (M = 59.38, SD = 5.37). Responses were hand scored by a research assistant. Responses were scored as correct if the participant provided the correct answer or a clear misspelling or minor morphological/wording variation (e.g., for the question *What did the Wright Brothers do before inventing an aircraft?*, correct answers included *built bicycles* or *bicycle manufacturers*). Errors were coded as errors of omission (i.e., no response was entered or a single letter was given) or errors of commission (i.e., an incorrect response). DR and DK responses were coded as such. Table A1 in the Appendix includes item-level information for the cued-recall task.

#### Accuracy

Overall, the distribution of responses varied substantially. Accuracy ranged from 0 to .98, with a mean of .33 (SEM = .01), indicating a wide range of question difficulty was successfully obtained. Errors ranged from 0 to .82 (M = .20, SEM = .01). The proportion of DR responses was overall low (M = .11, SEM = .005); however, the range of 0 to .56 does indicate some questions in the pool might be reflective of marginal knowledge. DK response rates ranged from 0 to .93 (M = .36, SEM = .013). Very few questions were left blank overall (M = .003, SD = .008), indicating participants were generally following instructions. Rank was calculated in order of increasing difficulty; ties were left as such in the database.

**Fig. 1:**
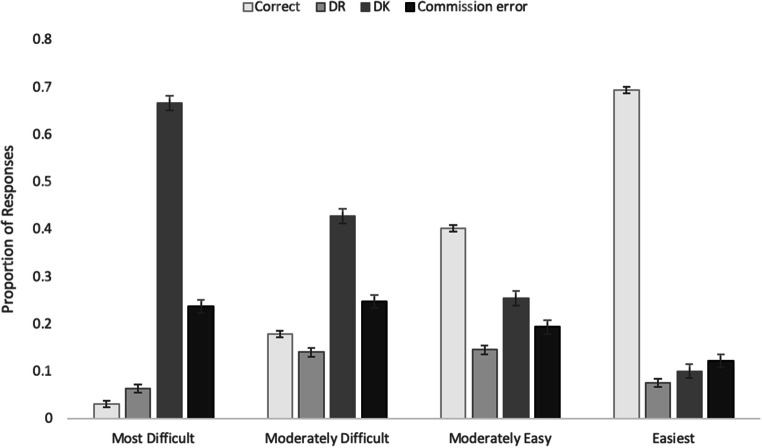
Proportion of correct, DR, DK, and commission errors as a function of question difficulty in Experiment 1 (cued-recall). *Error bars* represent standard error of the mean

To examine whether the rate of responses varied as a function of item difficulty, we binned all questions into quartiles, using accuracy as a proxy for difficulty. By binning the items into quartiles, we provide a basic stimulus selection framework for researchers, who might be interested in identifying items with a specific level of difficulty or items that elicit a high rate of DR or DK items. Because the data are necessarily ipsative and not independent (i.e., as the proportion of correct responses increases, proportions of other responses decrease), we focus on the descriptive statistics rather than providing any inferential statistics. Thus, the 421 questions were divided into quartiles ranging in overall accuracy from most difficult (M = .03, SEM = .007), to moderately difficult (M = .18, SEM = .007), to moderately easy (M = .40, SEM = .007), to easiest (M = .70, SEM = .01). As can be seen in Figure 1, generally, as correct responses increased, DK responses decreased. The rate of DR responses was similar for the most difficult and easiest questions, increased at intermediate levels of difficulty relative to the extremes, but was similar in the moderately easy and moderately difficult quartiles. Commission error rates were similar in the two most difficult quartiles, but decreased from the moderately difficult to the easiest.

The variable rates of DR and DK responses suggest that questions that are in a moderate difficulty range elicit the highest proportion of DR responses whereas questions that are too difficult elicit the highest rate of DK responses. This reflects the fact that general knowledge varies in accessibility as well as in availability (e.g., Tulving & Pearlstone, 1966). Thus, researchers interested in marginal knowledge or TOTs might select from the two intermediate quartiles, whereas those interested in learning of new GK might select from the most difficult quartile to avoid ceiling effects.

#### Response Times

Mean response times (RT) in milliseconds as a function of response (correct, DR, DK, or commission error) were calculated for each participant. DK responses were faster (M = 11413, SEM = 364) than all other response types, which were similar (M_*correct*_ = 17767, SEM = 475; M_*DR*_ = 18127, SEM = 364; *M*_*commission error*_ = 18385, SEM = 643).

**Fig. 2:**
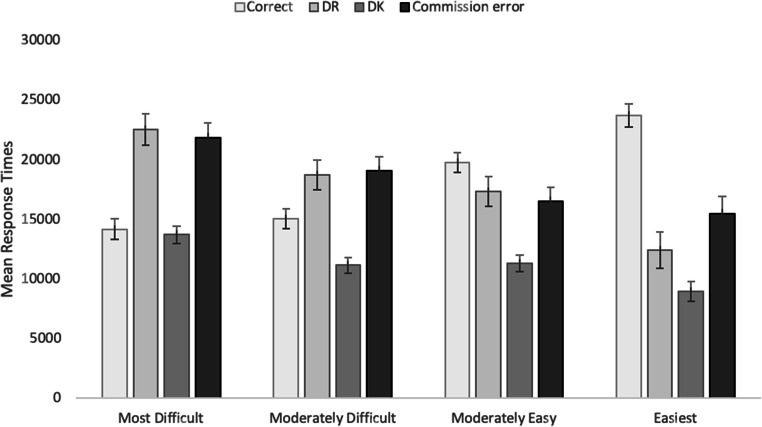
Mean response latencies as a function of difficulty and response in Experiment 1 (cued-recall). *Error bars* represent standard error of the mean

RTs as a function of item difficulty are presented in Fig. 2. Consistent with the accuracy analyses, we only report descriptive statistics. In general, RTs for correct answers increased as item difficulty decreased. This is likely due to the fact that more items were in this response category, rather than a cognitive processing explanation. In contrast, for the most difficult items, only a handful of correct responses were made. DK responses were generally faster and showed less change as a function of difficulty than other responses, presumably reflecting the fact that items receiving a DK response were rapidly identified as not being part of the knowledge base, and that this occurred regardless of item difficulty. DR responses tended to get faster as response accessibility increased, and errors showed a similar pattern. Overall, in concert with the accuracy data, the RTs suggest that items at intermediate levels of difficulty might fit into the category of marginal knowledge, resulting in relatively long searches through memory before a DR response is given. The relative slowness of DR responses is consistent with the idea that participants were searching the knowledge base prior to responding.

#### Comparison to Nelson and Narens (1980) and Tauber et al. (2013)

For the items in Set D, we performed an additional set of analyses comparing performance in our older adult sample to performance reported in the original studies. Tauber et al. noted that the rank order and overall accuracy of some questions had changed significantly over time due to changes in the knowledge base. However, because our older adult sample were young adults at the time Nelson and Narens’ data were collected, a cross-sectional analysis allowed us to examine whether the reported change in knowledge noted by Tauber et al. reflects changes at a global level or whether it is an artifact of the sample being tested. In other words, knowledge that was salient or accessible in 1980 might be inaccessible or unavailable to college students in 2012, but older adults might have preserved the knowledge that was relevant in their youth. Thus, it is possible that older adults’ performance now is more similar to that of young adults in 1980 than to young adults in 2012.

As was reported by Tauber et al. (2013), we compared the rank order of items in our database to the ranks from the two previous studies using Spearman’s *ρ*. Rank orders from Tauber et al. and from the Nelson and Narens (1980) norms were positively correlated to one another for the subset of questions included in our sample (*ρ* = .54, *p* < .001). The rank order in the present study was correlated to the orders in previous studies: Compared to Tauber et al. (*ρ* = .54, *p* < .001) and to Nelson and Narens (*ρ* = .67, *p* < .001) there appears to be both generational and longitudinal stability overall.

In addition to examining overall ranking of difficulty as Tauber et al. (2013) did, we examined accuracy across the three samples. The proportion of correct answers from the present norms, Tauber et al., and Nelson and Narens were entered into a one-way repeated measures ANOVA in which source was a between-items factor. Overall, accuracy on this subset of questions was highest in the older adults tested in the present sample (*M* = .24, *SEM* = .02), intermediate in the Nelson and Narens’ norms (M = .12, SEM = .01), and lowest in the Tauber et al. norms (M = .02, SEM = .002), F(1.28, 78.29) = 82.98, MSE = .014, *p* < .001, η_p_^2^ = .58 (all pairwise comparisons were reliable, *p*s < .001). This suggests that there is greater overlap in the knowledge base between today’s older adults and college students from the late 70s than between older adults and current young adults. Furthermore, although the knowledge base does increase over the lifespan (as evidenced by the higher accuracy among our older adults), the information that was known by young adults at the time of Nelson and Narens’ study appears to be preserved in older adults but very obscure to today’s younger adults. In concert with Tauber et al.’s conclusions about the need to regularly update general knowledge norms for younger adults due to fluctuations in the relevance of information, these findings point to the need to have age-appropriate norms for older participants as well. We acknowledge that these analyses are predicated on the strong assumption that age (or cohort) is the main factor that distinguishes our sample from the college students tested in the Nelson and Narens and Tauber et al. studies. Clearly, other factors, such as overall educational achievement, individual and group differences in information-seeking behaviors, and even personality characteristics such as conscientiousness, might contribute to the age-related differences. We return to these points in the General Discussion.

## Experiment 2

Because general knowledge questions are used across a variety of tasks, in addition to providing cued-recall performance, we administered a multiple-choice version of the task to a set of new participants, all from online sources. Multiple-choice questions provide additional environmental support and, on tests that do so, such as item recognition, older adults often show reduced performance deficits relative to younger adults (Craik, 1983, 1986; Craik & Byrd, 1982; see also, Bäckman & Nilsson, 1985; Charness & Bosman, 1995). Thus, providing normative data for multiple-choice questions in addition to cued-recall questions will allow researchers greater control over baseline performance differences. In addition, a substantial portion of what any individual has stored in memory might fluctuate in accessibility, and this is especially true for older adults (Umanath, 2016). Retrieval success can vary within individuals even in the absence of corrective feedback following an initial retrieval attempt (Fazio, Barber, Rajaram, Ornstein, & Marsh, 2013; Heine et al., 1999; Umanath, 2016). In other words, different tests and paradigms provide converging evidence that what can be assessed on a single test may not accurately reflect the contents of the knowledge base, especially in OAs. However, older adults might also be negatively affected by the presence of related foils on a multiple-choice test, due to deficits in inhibitory processes (Hasher & Zacks, 1988). Incorrect information presented as foils might create challenges due to increased familiarity, which, coupled with the difficulty older adults might have in ignoring irrelevant information, could lead to greater error rates, especially for items that fall within a marginal knowledge zone.

### Participants

A total of 201 participants were recruited. All participants were recruited on MTurk, using the same restrictions described above. Data from five participants were omitted because they self-reported their age under 55 (three from Set A and one each from Sets C and D), leaving 49 participants in each set. All but four participants (two in Set B and two in Set C) reported being native speakers of English (see Table 2 for demographic information). Because we had restricted participation to US IP addresses and because we had no evidence that these participants were incapable of performing the task (their accuracy rates and response times were comparable to the native English speakers), we did not omit them from the database.

**Table 2:** Demographic information for participants in Experiment 2

	*N*	Age (SD)	Education (SD)	*N* Women (%)
Set A	49	64.16 (5.94)	15.51 (2.35)	32 (65)
Set B	49	62.10 (4.37)	15.61 (3.13)	28 (57)
Set C	49	63.45 (5.73)	15.25 (2.34)	38 (78)
Set D	49	62.65 (5.27)	15.74 (2.57)	36 (73)

Six participants made an error on one bot check but correctly answered the other. After examining their overall accuracy and RTs compared to the rest of the sample, we determined to leave these participants in the analyses, because they did not appear to differ from the rest of the sample on either measure (i.e., their overall accuracy and RTs were not outliers, which would be expected if participants were guessing, pressing keys at random, or failing to read the questions). We also confirmed that these participants were not pressing the same key on every trial nor were they responding quickly.

### Materials

The same sets of questions were used. For each question, three alternative, incorrect responses were added. For set A, the foils were developed in the lab or were selected from the online quizzes where provided. For the questions in Set B, the Burke et al. (1991) foils were obtained from the original set of materials; those from Wang et al. (2016) were developed in our lab. In Set C, all foils were from the original source (Cantor et al., 2015). For the Set D items (those from Nelson & Narens, 1980), foils were developed in the lab. Foils developed in the lab were selected as follows. High frequency errors from an earlier pilot study were selected, as well as alternatives generated by research assistants. These were developed by choosing items that are either from the same category as the correct answer, or closely related with the correct answers. For example, for the question “*What is the last name of the criminal who was killed by FBI agents outside of a Chicago movie theater*”, to which the correct answer is *Dillinger*, names of criminals who were called “Public Enemies” by the FBI, just as Dillinger was, were listed as foil choices. The Appendix includes the percentage of times each foil was selected (this information might be of use to researchers interested in eliciting errors). We opted not to include DR and DK as options out of concern that participants might overly rely on these options in the face of uncertainty, thereby potentially reducing the number of responses.^6^ The two bot check questions, presented at random points throughout the task, required participants to select a specific response option.

### Procedure

The procedure was very similar to that used in Experiment 1, and the study was programmed using Gorilla software (Anwyl-Irvine et al., 2019). After providing consent and answering basic demographic information, participants were presented the GK questions, one at a time, in random order. The position of the correct answer varied across the four options approximately an equal number of times. Participants were allowed unlimited time for each question and were asked to not consult any external sources.

### Results and Discussion

#### Accuracy

Item-level information is presented in Table A2 in the Appendix. Rank for the multiple-choice questions was calculated separately from the rank for cued-recall. The rank order correlation between the cued-recall and multiple-choice tests was high and positive, *ρ*(419) = .81, *p* < .001, although not perfect, indicating some variability in performance as a function of the demands on the retrieval process. To examine whether item difficulty based on the cued-recall task also predicted accuracy in multiple-choice, we used the quartile bins based on cued recall accuracy from Experiment 1 to analyze multiple choice accuracy in a one-way ANOVA with difficulty as a between-items factor, accuracy increased across quartiles, from .29 (*SEM* = .02) for the most difficult questions to .51 (*SEM* = .02), to .69 (*SEM* = .02), to .86 (*SEM* = .02) for the easiest questions, *F*(3, 417) = 212.00, *MSE* = .03, *p* < .001, η_p_^2^ = .60. At all levels of difficulty, performance was above chance, estimated at .25 given four alternatives (all *p*s ≤ .04). Thus, the questions that were difficult to answer in one format were similarly difficult in the other format.

#### Response Times

**Fig. 3:**
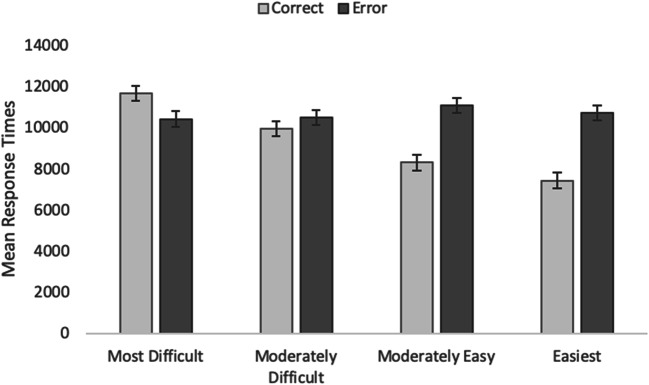
Average response times in the multiple-choice task as a function of accuracy and question difficulty in Experiment 2. *Error bars* represent standard error of the mean

RTs to correct and incorrect multiple-choice questions were examined in a one-way ANOVA as a function of question difficulty using the cued-recall rank as a between-items factor (Fig. 3). This analysis provides an indirect means of examining accessibility, such that easier items are expected to be answered correctly relatively quickly but more difficult items might take longer, and errors might even be faster, if the foils are familiar. Overall, RTs increased as question difficulty increased, *F*(3, 413) = 8.84, MSE = 16433694, *p* < .001, η_p_^2^ = .06. Correct responses (M = 9364, SEM = 183) were faster than errors (M = 10715, SEM = 187), *F*(1, 413) = 31.34, MSE = 12122700, *p* < .001, η_p_^2^ = .07. The interaction was significant, *F*(3, 413) = 18.93, MSE = 12122700, *p* < .001, η_p_^2^ = .12. For easy and moderately easy questions, correct responses were faster than errors for the easier items (*t*[100] = 7.08, *p* < .001, *d* = .70; and *t*[104] = 6.58, *p* < .001, *d* = .64, respectively); however, as difficulty increased, the pattern reversed, such that errors were slightly faster than correct responses for the most difficult items *t*(105) = 2.0, *p* = .047, *d* = 20. RTs for moderately difficult items did not differ as a function of accuracy, *t*(104) = 1.44, *p* = .15, *d* = .14.

#### Comparison between Cued-Recall and Multiple-Choice Performance

**Fig. 4:**
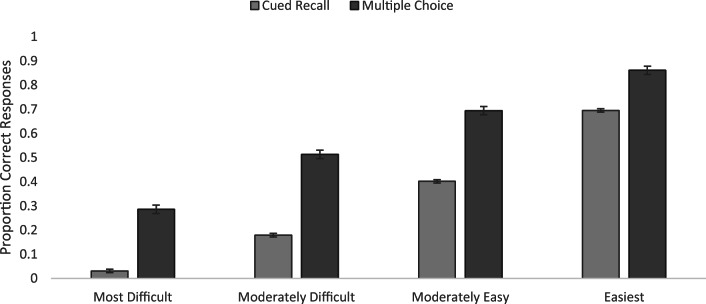
Cued-recall vs. multiple-choice accuracy as a function of difficulty. *Error bars* represent standard error of the mean

To examine more directly the differences between cued-recall and multiple-choice testing, we compared accuracy as a function of test type and question difficulty in a 2 (test type) x 4 (question difficulty based on cued recall performance) mixed ANOVA. Unsurprisingly, accuracy was higher in the multiple-choice format (M = .59, SEM = .008) than in the cued-recall format (M = .33, SEM = .004), *F*(1, 417) = 1018.79, MSE = .014, *p* < .001, η_p_^2^ = .71 (cf. Craik & Byrd, 1982). A significant interaction emerged, *F*(3, 417) = 19.41, MSE = .014, *p* < .001, η_p_^2^ = .12. The interaction (see Fig. 4) was driven by the fact that the difference in mean accuracy as a function of test type varied as a function of difficulty, with smaller discrepancies between test formats when items were easy (M = .17) than when items were more difficult (M = .25, for the most difficult items, M = .33, for the moderately difficult items, and M = .29, for the moderately easy items).

The Appendix includes a difference score between accuracy on the multiple choice and accuracy on the cued recall versions of the same question. Although for the vast majority of questions (398) performance was better in the multiple-choice task, a subset of questions was answered correctly more in the open-ended version (*n* = 23). Thus, there appear to be some GK items that are more affected by incorrect foil presentation than others; although we acknowledge a very small set in our database fit this description. Overall, items that are more accessible in a cued-recall task also seem to be more accessible in a multiple-choice task. One caveat of this conclusion is that different participants completed the two tasks; future studies aimed at more directly assessing the relative accessibility or availability of specific units of knowledge might use alternative methods, such as directly comparing free recall, cued-recall, and recognition in a within-participants design. Alternatively, methods such as Buschke’s (1973) selective reminding procedure would allow researchers to more directly examine the effects of repeated retrieval attempts on non-retrieved items and assess marginal knowledge or temporary retrieval failures, to discriminate them from failures in accessibility.

## General Discussion

This study presents normative data from older adults on over 400 general knowledge questions ranging in difficulty. For each question, the database includes cued-recall and multiple-choice accuracy and response latencies. The cued-recall data further include information about the phenomenological responses associated with retrieval failures (DR and DK; Coane & Umanath, 2019), a novel measure of metacognition that is based in natural language use. DR responses reflect retrieval failures based on a (potentially temporary) lack of accessibility and DK responses are associated with a lack of availability (or storage) in memory (Tulving, 1985). For many of the items included in the norms, data from younger adults are available elsewhere (e.g., Cantor et al., 2015; Tauber et al., 2013). Norms exclusively for older adults were, to our knowledge, not previously available.

The main results can be summarized as follows: As item difficulty, estimated based on the proportion of correct responses, increased, the proportion of DR responses in cued-recall increased from the most difficult to the moderately easy, and then decreased again for the easiest items, showing a somewhat inverted U-shaped pattern. DK responses, in contrast, decreased consistently. Jointly, this suggests that as accessibility increases, non-retrieved items are less likely to be judged as not known and somewhat more likely to be judged as not remembered. Thus, moderately easy and moderately difficult items, which are correctly retrieved by older adults between 20 and 40% of the time, might be targets for studies on marginal knowledge. However, it is important to note that in a multiple-choice test, even moderately easy items were correctly identified over 70% of the time. In fact, even the most difficult items were correctly identified at above chance levels. These data underscore the importance of using normative data based on the type of test participants will be completing.

The availability of age-appropriate general knowledge questions can facilitate research in a variety of ways. The wide range of difficulty in the present norms will provide researchers the ability to select stimuli to meet specific needs. If the goal is to identify a set of to-be-learned materials that allow a wide range of prior familiarity or knowledge, while keeping an experimental paradigm reasonably short, starting with normative data can be useful. For example, if researchers are interested in assessing feeling-of-knowing and obtaining a wide range of responses, these norms provide a large set of items from which to select targets. In situations in which the goal is to minimize prior knowledge, to assess learning or acquisition of new knowledge, more difficult items can be selected. This can be particularly useful in research examining effective learning strategies in older adults; although much research uses paired associates or word lists (e.g., Coane, 2013; Pastötter & Baüml, 2019) or more complex prose passages (e.g., Roediger & Karpicke, 2006; see Rowland, 2014; van Gog & Sweller, 2015, for reviews), there are experimental paradigms and conditions in which meaningful yet easily controlled material is necessary. As another example, measuring marginal knowledge or TOT states can be challenging when participants’ prior knowledge levels are not known. Through these norms, researchers can select subsets of items that fall within the desired range of difficulty, to increase the likelihood of obtaining the desired distributions of retrieval successes and failures.

Much research in cognitive aging includes young adult participants as well. As noted in the Introduction, older adults typically outperform younger adults in most measures of knowledge, from vocabulary to general knowledge (Salthouse, 2004; Umanath & Marsh, 2014). Thus, it can be challenging to obtain accurate measures of age-related differences due to marked disparities in baseline performance. If the goal of a particular research study is to examine performance across age while controlling for overall difficulty, the same items for both age groups might not be ideal. In combination with the excellent norms of Tauber et al. (2013), the present norms can allow researchers to carefully select items to yield equivalent baseline levels of performance, thus rendering age-related differences more transparent. Existing databases do include extremely difficult/not known information for younger adults already. Identifying extremely difficult items for older adults, however, can be a challenge for researchers. The present database includes a set of such items. For example, a number of items in the database yielded extremely low rates of accurate recall by older adults. These items could be selected in concert with items that yield similar rates of recall in younger adults from existing databases to provide similar baseline performance levels in studies comparing age-related changes in performance.

The items included in these norms cover a wide variety of topics, from geography to literature to science. More nuanced analyses could reveal some insights into what bodies of knowledge older adults have strengths and gaps in knowledge. Although the preservation, or even increase, of knowledge in aging is well documented, it is still unclear exactly what the content of this knowledge is beyond the impressive but small body of work done by Bahrick and colleagues (see Bahrick et al., 2013, for a review). The present norms provide some initial insight into what older adults do and do not know, as well as what they identify as not remembered. In our earlier work (Coane & Umanath, 2019), not remembering was associated with forgetting or with a temporary retrieval failure. Thus, in these norms, high rates of DR might indicate information that older adults identify as once having been known, but forgotten, or information that is known but not accessible at that point in time. Such items are likely to be learned (or re-learned) more quickly than items identified as not known, which is assumed to reflect content deemed to be outside of the knowledge base (see Coane & Umanath, Experiment 2). Items with a high rate of DR responses, in particular, might be of use to researchers interested in the fluctuation of knowledge or in TOT states. Given the evidence that word-finding difficulties reported by older adults are more common for proper names (see Paolieri, Marful, Morales, & Bajo, 2018), additional analyses might examine whether retrieval failures occur more often for questions in which an individual’s name is sought after compared to questions that tap into more conceptual knowledge or object names. In addition, researchers interested in examining other measures of retrieval failure, such as feeling-of-knowing (FOK; Hart, 1965), could select a mixture of items that elicited high DR response rates (which would be expected to yield a high FOK rating) as well as items that elicited fast or slow DK responses (which should elicit low FOK and intermediate FOK responses, respectively). The inclusion of response time information can further inform researchers on the extent to which participants are engaging in a search through memory. Slower retrieval latencies reflect longer searches, whereas rapid responses can indicate easy access, in the case of correct answers, or an effortless assessment that the information is not in the knowledge base, in the case of DK responses.

A secondary set of analyses reported in the Results of Experiment 1 illustrates the importance of identifying age-specific normative performance. A subset of the questions included here were selected from the Nelson and Narens (1980) norms, which were updated by Tauber et al. (2013). Comparison of performance on these items across the three databases revealed two key findings: First, older adults outperformed college-aged students in both studies, confirming prior findings that knowledge increases over the lifespan; and, second, whereas participants in Tauber et al.’s study performed worse than those in Nelson and Narens’ study, reflecting declines in the availability or certain items, older adults’ rate of correct responses increased, suggesting some knowledge preservation over the course of four decades. It is likely that the most difficult items in this database, which were close to floor for older adults, would be virtually impossible for younger adults. We acknowledge that these analyses were based on a subset of the items in the norms and that we had specifically selected the most difficult items in the previous norms, thereby raising the concern of floor effects in younger adults. Such analyses are also, necessarily, based on different cohorts.

As discussed in the Introduction, cohort effects in vocabulary have been repeatedly reported and have been attributed to a number of factors (e.g., reading habits, education, item selection effects; Verhaeghen, 2003). Castro et al. (2020) recently reported both stability and change in a category norming task as a function of age. Taken together, these studies point to the importance of examining cohort differences in a variety of measures of crystallized knowledge.

One important way in which the samples tested here and those included in the previous studies differ concerns student status and overall educational attainment. Whereas participants in Tauber et al. (2013) and Nelson and Narens (1980) were current college students, the older adults were not. Although the mean number of years of education for our participants in both studies was between 15 and 16, suggesting post-secondary education for the majority of participants, we cannot ensure that the final educational status of the younger adults would be comparable. Thus, in addition to age, there is a possible confound of education across samples. To examine whether education level affected performance in the present sample, we conducted some additional analyses at the participant level by correlating education with accuracy. In the recall task, over all sets, education was positively correlated with accuracy (*r* = .24, *p* < .001). Although significant, the effect was not particularly large, accounting for less 6% of the variance. This pattern held in all sets (B: *r* = .33, *p* = .013; C: *r* = .30, *p* = .015; D: *r* = .33, *p* = .008), except for Set A (*r* = .07, *p* = .7), which included the most difficult questions, suggesting that education predicts general knowledge level across a range of difficulty, but not when items are largely inaccessible. There was no systematic relation between rate of DR responses and education (*r* = .01). Years of education were negatively correlated with DK responses for all sets (all *r*s ≤ -.35, *p*s ≤ .012, other than Set D (*p* = .63), in which DK was the modal response. This suggests that some items are simply not known and do not depend on educational attainment. Note that the items in Set D were specifically selected based on young adult norms to be especially difficult. In the multiple-choice task, there was an overall modest positive correlation between education and accuracy (*r* = .15, *p* = .04). Within each set of questions, the correlation was only significant for participants in Set C (*r* = .33, *p* = .02), which had the highest rate of correct answers overall. Thus, education, not surprisingly, does affect accuracy and presumably the knowledge base of participants.

According to several theories of intelligence (e.g., Hayes, 1962; von Strumm & Ackerman, 2013), knowledge, or crystallized intelligence, is the result of accumulated experiences over time. As Cattell (1963) suggested, fluid intelligence can transform into crystallized intelligence. Importantly, individual differences in knowledge seeking lead to different behaviors and thus to differences in crystallized intelligence. Individuals or groups who are more likely to seek out learning opportunities will thus accumulate more knowledge. College students presumably are peak information gatherers, whereas it is less evident to what extent older adults in the present sample can be described in such terms. Furthermore, according to Carstensen’s socio-emotional selectivity theory (1992, 2006), as individuals age, their focus shifts from information-seeking to relational and emotional goals. Thus, it is possible that cohort differences in terms of information and knowledge seeking behavior impact the results of this study. However, we note that, if anything, older adults would be expected to engage in less information seeking than younger counterparts, making the differences in performance for those items for which young adult data are available even more compelling in terms of knowledge preservation in aging. Another factor that might explain the relatively high performance of older adults in the present study is greater conscientiousness. This personality trait, which is associated with being hard-working and task-oriented, has been found to increase from early adulthood to middle age and is typically higher in older adults than college aged adults (e.g., Donnellan & Lucas, 2008) and individuals higher in this domain perform better on memory tests and overall measures of cognitive status (e.g., Luchetti, Terracciano, Stephan, & Sutin, 2016). Thus, older adults might be more likely to put effort into the task and thus might retrieve more knowledge, in addition to having a larger knowledge base.

Although we have been framing the age differences observed in terms of differences in overall contents of the knowledge base and the retrieval of this knowledge, it is important to consider another factor: Some of the information may not have been available to some participants due to lack of exposure. What is accessible in any individual’s memory store is going to depend not only on the ability to retrieve this knowledge when needed, but on whether the information is available in the first place. Similarly, forgetting is going to depend on the extent to which knowledge was originally known. In brief, knowledge not only varies within individuals, but across time – what is popular or broadly discussed in media or taught in schools varies, so the failure to retrieve may be due not to forgetting but to failure to learn in the first place, given shifts in educational curricula, media discussions, and access to informal learning such as what might be acquired through television, movies, or other forms of entertainment. A similar point was highlighted by Tauber et al. (2013): Whereas few participants in the original Nelson and Narens’ (1980) study knew the capital of Iraq is Baghdad, this information was largely known by participants tested after the US invasion of Iraq, given widespread news coverage of the Middle East.

We conclude our discussion by briefly addressing some additional limitations. First, although most of the items have been tested elsewhere with younger adult samples, a subset of our items was developed uniquely for the present norming study; thus, younger adult norms are not available. Second, as highlighted by Tauber et al. (2013), normative data need to be regularly updated and adapted for changes over time. Thus, the present norms should be updated and extended to other samples. Third, the present norms might be specific to participants in the United States, and not all items may generalize to other cultures and countries. Although a subset of items is likely to be relatively universal (e.g., those relating to science or geography), others might be somewhat culturally bound (e.g., items relating to popular culture or history). Fourth, as with most research studies, our sample was based on specific populations (i.e., participants living near colleges or with access to online data collection sites). However, it is worth noting that our samples were broadly diverse in terms of geographic location, thus, it is possible they represent a reasonable swath of the US older adult population likely to participate in future research. It is important to note that half of our sample in Experiment 1 and all participants in Experiment 2 were tested online, unlike participants in earlier studies. Although this might be a concern given unknown differences between research participants enrolled online and those recruited for in-person laboratory studies, the online data collection has the advantage of recruiting from a more diverse sample in terms of location, education, and cultural background than might be the case in the communities surrounding research institutions.

In conclusion, the norms presented here will be useful for researchers interested in a wide range of topics in the area of cognitive aging. Having access to normed stimuli can reduce the cost and time associated with pilot studies to determine baseline levels of knowledge and accelerate the rate of understanding what is preserved and what declines in healthy cognitive aging. Future studies could include participants with known memory or other cognitive declines, to better characterize the changing nature of knowledge in disordered aging and to inform further theoretical and model development concerning the interactive nature of lexical and semantic knowledge and how overall cognitive performance is affected by these factors (Wulff et al., 2019).

## Supplementary Information

ESM 1 (DOCX 463 kb)

## Footnotes

1 Examination of the questions we selected from the Tauber et al. (2013) norms mostly reflected what might be considered relatively stable knowledge, such as questions about history, geography, and literature and the arts. There were not enough questions in our selection to allow us to compare items from the popular culture of the time to this more stable GK. Thus, we cannot directly address the extent to which specific information may be more or less relevant in a given historical era.

2 In both of the studies conducted in the lab, the two experimental conditions differed in the nature of the final test (multiple choice vs. cued recall); the encoding phase from which the present data were collected was similar. Specifically, both groups of participants answered the same questions under the same time parameters (self-paced) and were given the same instructions. One group of participants was given correct answer feedback after their response attempt; however, there were no differences in overall performance in the task, suggesting that the presence of feedback did not systematically affect participants’ response strategies (see Coane & Umanath, 2019, for details). In fact, performance on the initial task was very similar across conditions in both experiments. Thus, the data from both conditions were combined for each set of participants.

3 Sets C and D also included some easy filler questions that elicited almost 100% correct recall (16 in set C and 30 in set D); these are not included here.

4 We do not have data indicating whether participants left the page during the task; thus, we cannot rule out that participants looked up some answers using other sources. Of course, given the wide availability of smartphones, even such a measure would not guarantee participants did not search online for information.

5 Following this phase, lab participants completed either a final multiple-choice test or a cued recall test (these data are not reported here; see Coane & Umanath, 2019; Umanath et al., n.d.). Following the final test, participants responded to two questions addressing what they meant when they said DR or DK and completed a measure of vocabulary (Shipley, 1940) and the MMSE (Folstein, Folstein, & McHugh, 1975).

6 Because the multiple-choice test presents the correct answer, it seemed unlikely participants would be in a tip-of-the-tongue state or other accessibility failures. Also, we have not examined the use of DR/DK in multiple choice tests previously; thus, it is unclear to what extent participants would use it to indicate lack of confidence or actual failures in availability or accessibility. We know the properties of this measure in recall tasks, but we have not examined the retrieval dynamics that would lead someone to endorse DR or DK in a recognition context. Thus, to avoid interpretation issues that could confound the utility of the data, we opted to not include these as response options. Furthermore, recognition is easier than recall, especially for older adults, and the inclusion of these response options might have resulted in an underestimation of knowledge.
