# Supplementary material for: A database of general knowledge question performance in older adults
Source: Behav Res Methods. 2021 Jan 14;53(1):415–29. doi: 10.3758/s13428-020-01493-2 (PMC7880974; doi:10.3758/s13428-020-01493-2)
Supplement: Supplementary file 1 — (DOCX 463 kb) [file 13428_2020_1493_MOESM1_ESM.docx]

Appendix

Table A1

*Item Level Cued Recall Performance (Proportion of Responses and Response Times)*

|  |  |  |  |  |  | CR (proportion of responses) | | | | | CR (response times) | | | | |
| --- | --- | --- | --- | --- | --- | --- | --- | --- | --- | --- | --- | --- | --- | --- | --- |
| Set | CR Rank | T. et al. rank | N & N rank | General Knowledge Question | Correct Answer | Correct | DR | DK | CE | OE | Correct | DR | DK | CE | OE |
| B | 1 | NA | NA | Which band was Paul McCartney a member of? | The Beatles | .98 | .00 | .00 | .02 | .00 | 8611 |  |  | 4582 |  |
| B | 2 | NA | NA | What is the short pleated skirt worn by Scottish men? | Kilt | .96 | .00 | .00 | .04 | .00 | 7763 |  |  | 11733 |  |
| B | 3 | NA | NA | What is the hard, white material sourced from elephant tusks? | Ivory | .94 | .00 | .04 | .02 | .00 | 6901 |  | 14494 | 9386 |  |
| B | 4 | NA | NA | What word means to trade by exchanging goods for other goods rather than money? | Barter | .93 | .00 | .02 | .04 | .02 | 8453 |  | 29489 | 32738 | 1364 |
| B | 5 | NA | NA | What is an airplane without an engine called? | Glider | .89 | .02 | .07 | .02 | .00 | 6880 | 5430 | 7717 | 32545 |  |
| C | 6 | NA | NA | What is the term for the first aid instrument which stops the flow of blood in an artery preventing it from being lost through a wound? | Tourniquet | .88 | .00 | .01 | .10 | .00 | 23202 |  | 16600 | 39152 |  |
| B | 7 | NA | NA | What is the name of the nylon fabric which has two pieces which stick to each other and is used as a fastener? | Velcro | .87 | .04 | .04 | .06 | .00 | 12326 | 183957 | 62285 | 11946 |  |
| C | 8.5 | NA | NA | What is the last name of the person who sang 'I did it my way'? | Sinatra | .87 | .03 | .03 | .06 | .01 | 10785 | 10784 | 5366 | 14341 | 6594 |
| C | 8.5 | NA | NA | What is the name of the song traditionally sung at the stroke of midnight on New Year's Eve? | Auld Lang Syne | .87 | .04 | .00 | .09 | .00 | 18159 | 41149 |  | 23715 |  |
|  |  |  |  |  |  |  |  |  |  |  |  |  |  |  |  |
|  |  |  |  |  |  | CR (proportion of responses) | | | | | CR (response times) | | | | |
| Set | CR Rank | T. et al. rank | N & N rank | General Knowledge Question | Correct Answer | Correct | DR | DK | CE | OE | Correct | DR | DK | CE | OE |
|  |  |  |  |  |  |  |  |  |  |  |  |  |  |  |  |
| B | 11 | NA | NA | What is the last name of the boxer who later become known as Mohammed Ali? | Clay | .85 | .04 | .07 | .02 | .02 | 9379 | 21630 | 5736 | 27818 | 1288 |
| B | 11 | NA | NA | What is the traditional daytime sleep in Spain? | Siesta | .85 | .00 | .07 | .06 | .02 | 9749 |  | 12661 | 10791 | 1300 |
| B | 11 | NA | NA | In what park is "Old Faithful" located? | Yellowstone | .85 | .00 | .04 | .11 | .00 | 9123 |  | 2761 | 8365 |  |
| B | 14.5 | NA | NA | What is the name of the art of Japanese paper folding? | Origami | .83 | .04 | .11 | .02 | .00 | 7933 | 9414 | 7108 | 4293 |  |
| B | 14.5 | NA | NA | Who was England's prime minister during World War II? | Churchill | .83 | .04 | .09 | .04 | .00 | 10940 | 31309 | 6683 | 11696 |  |
| B | 14.5 | NA | NA | What is the word "memo" short for? | Memorandum | .83 | .00 | .04 | .13 | .00 | 10208 |  | 30041 | 17991 |  |
| B | 14.5 | NA | NA | What is the colored portion of the eye? | Iris | .83 | .00 | .09 | .07 | .00 | 8243 |  | 6351 | 14192 |  |
| C | 17 | NA | NA | What is the name of the shell from which people hear the sea? | Conch | .82 | .03 | .03 | .12 | .00 | 8982 | 12665 | 10463 | 20702 |  |
| B | 18.5 | NA | NA | What do you call the vessel, usually an ornamental vase on a pedestal, which is used to preserve the ashes of the dead? | Urn | .81 | .04 | .09 | .06 | .00 | 13077 | 25170 | 12579 | 12573 |  |
| B | 18.5 | NA | NA | What do you call an instrument for performing calculations by sliding beads along rods or grooves? | Abacus | .81 | .06 | .09 | .02 | .02 | 10870 | 24474 | 28189 | 30351 | 1201 |
| C | 20 | NA | NA | What device blows air on to a fire to make it hotter? | Bellows | .81 | .07 | .03 | .07 | .01 | 10347 | 20636 | 21092 | 25225 | 8690 |
|  |  |  |  |  |  |  |  |  |  |  |  |  |  |  |  |
|  |  |  |  |  |  | CR (proportion of responses) | | | | | CR (response times) | | | | |
| Set | CR Rank | T. et al. rank | N & N rank | General Knowledge Question | Correct Answer | Correct | DR | DK | CE | OE | Correct | DR | DK | CE | OE |
|  |  |  |  |  |  |  |  |  |  |  |  |  |  |  |  |
| B | 21 | NA | NA | What is the proper name for a "tidal wave"? | Tsunami | .80 | .06 | .09 | .06 | .00 | 16305 | 15825 | 12321 | 7275 |  |
| C | 22.5 | NA | NA | What is a goat's offspring called? | Kid | .79 | .07 | .03 | .09 | .01 | 6630 | 20904 | 7186 | 14710 | 20997 |
| C | 22.5 | NA | NA | What optical instrument allows crews of submerged submarines to look at surface ships? | Periscope | .79 | .01 | .03 | .16 | .00 | 11907 | 13929 | 22403 | 24938 |  |
| B | 25.5 | NA | NA | What is the term for nautical mile per hour? | Knot | .78 | .04 | .13 | .06 | .00 | 11021 | 9352 | 7664 | 7696 |  |
| B | 25.5 | NA | NA | What is the name of the loch (lake) in Scotland that is supposedly home of a legendary monster? | Ness | .78 | .06 | .09 | .07 | .00 | 11120 | 12073 | 9026 | 19304 |  |
| B | 25.5 | NA | NA | What is the name of the Egyptian plant that is cut into strips and pressed into a material to write on? | Papyrus | .78 | .02 | .15 | .04 | .02 | 15293 | 9127 | 9356 | 7977 | 3148 |
| B | 25.5 | NA | NA | Which video game character is a plumber? | Super Mario | .78 | .02 | .19 | .02 | .00 | 8654 | 32307 | 6906 | 6511 |  |
| C | 28 | NA | NA | What is the craft of tying knots to make belts, bags, and plant hangers? | Macrame | .76 | .13 | .07 | .03 | .00 | 11578 | 27153 | 20167 | 38612 |  |
| D | 29 | 189 | 157 | What is the last name of the doctor who first developed a vaccine against polio? | Salk | .76 | .14 | .06 | .05 | .00 | 9560 | 19466 | 24428 | 27554 |  |
| C | 31 | NA | NA | What is the name of the hillbilly family who had the famous feud with the McCoys? | Hatfield | .75 | .16 | .01 | .06 | .01 | 10934 | 28654 | 8260 | 25953 | 41363 |
| C | 31 | NA | NA | What is the name of the man who created the comic strip 'Peanuts'? | Shultz | .75 | .16 | .01 | .06 | .01 | 11725 | 14732 | 11453 | 18545 | 19988 |
|  |  |  |  |  |  | CR (proportion of responses) | | | | | CR (response times) | | | | |
| Set | CR Rank | T. et al. rank | N & N rank | General Knowledge Question | Correct Answer | Correct | DR | DK | CE | OE | Correct | DR | DK | CE | OE |
| C | 31 | NA | NA | In mammals the period of time between fertilization of the egg and the birth of the young is known by what scientific term? | Gestation | .75 | .03 | .04 | .18 | .00 | 15394 | 34180 | 21189 | 23490 |  |
| B | 33 | NA | NA | Which was the 49th state to join the Union? | Alaska | .74 | .00 | .09 | .17 | .00 | 14660 |  | 8934 | 15111 |  |
| C | 34.5 | NA | NA | What is the term frequently applied to Christ's doctrine of doing to others as we would wish them to do unto us? | Golden Rule | .73 | .09 | .04 | .13 | .00 | 14155 | 27071 | 11225 | 57917 |  |
| C | 34.5 | NA | NA | What was the name given to the geographic line in the United States which was the separation of slavery and freedom? | Mason-Dixon Line | .73 | .15 | .03 | .09 | .00 | 15053 | 31728 | 29593 | 23151 |  |
| D | 36 | 181 | 150 | Of which country is Budapest the capital? | Hungary | .73 | .02 | .03 | .23 | .00 | 12079 | 12334 | 8975 | 19847 |  |
| B | 38.5 | NA | NA | What do you call a question that is asked for effect with no answer expected? | Rhetorical | .72 | .09 | .09 | .09 | .00 | 19252 | 18609 | 8903 | 16234 |  |
| B | 38.5 | NA | NA | What word means to formally renounce a throne? | Abdicate | .72 | .04 | .17 | .07 | .00 | 10897 | 15910 | 8556 | 17215 |  |
| B | 38.5 | NA | NA | Which president suffered from polio? | Roosevelt | .72 | .09 | .07 | .11 | .00 | 14592 | 7243 | 8777 | 14247 |  |
| B | 38.5 | NA | NA | What do you call a savory sauce in which meat, fish, or a vegetable is soaked before cooking to enhance the flavor? | Marinade | .72 | .06 | .04 | .17 | .02 | 13633 | 33683 | 7328 | 19297 | 3417 |
| C | 41.5 | NA | NA | What is the term for someone who doubts but does not deny the existence of God? | Agnostic | .72 | .06 | .01 | .19 | .01 | 12441 | 31471 | 14796 | 22470 | 27294 |
|  |  |  |  |  |  |  |  |  |  |  |  |  |  |  |  |
|  |  |  |  |  |  |  |  |  |  |  |  |  |  |  |  |
|  |  |  |  |  |  | CR (proportion of responses) | | | | | CR (response times) | | | | |
| Set | CR Rank | T. et al. rank | N & N rank | General Knowledge Question | Correct Answer | Correct | DR | DK | CE | OE | Correct | DR | DK | CE | OE |
| C | 41.5 | NA | NA | What was the name of the body of water where Thoreau studied for two years? | Walden Pond | .72 | .04 | .16 | .06 | .01 | 13551 | 22281 | 12283 | 18319 | 18023 |
| B | 44 | NA | NA | Where was the Declaration of Independence signed? | Philadelphia | .70 | .06 | .04 | .19 | .02 | 12027 | 6227 | 8907 | 14219 | 4935 |
| B | 44 | NA | NA | What word means to shed hair, feathers, or an outer layer periodically, as with the changing seasons? | Molt | .70 | .15 | .11 | .04 | .00 | 13636 | 25854 | 14925 | 6364 |  |
| B | 44 | NA | NA | What is the musical term that means "without instrumental accompaniment"? | A capella | .70 | .07 | .13 | .09 | .00 | 15668 | 36799 | 13172 | 7133 |  |
| C | 47.5 | NA | NA | What do we call the bone which composes the lower jaw? | Mandible | .70 | .03 | .12 | .15 | .00 | 13662 | 19805 | 17737 | 19754 |  |
| C | 47.5 | NA | NA | What is the last name of the first female pilot to cross the Atlantic? | Earhart | .70 | .19 | .04 | .06 | .00 | 18780 | 29294 | 82532 | 174108 |  |
| C | 47.5 | NA | NA | What nickname was given to Northerners who went south after the Civil War and took advantage of Southern poverty? | Carpetbaggers | .70 | .10 | .07 | .12 | .00 | 14557 | 21740 | 31168 | 21197 |  |
| C | 47.5 | NA | NA | What was the last name of the author who wrote the fable about the fox who assumed the grapes he couldn't reach were sour anyway? | Aesop | .70 | .06 | .19 | .03 | .01 | 13237 | 13150 | 13333 | 17003 | 8411 |
| C | 50 | NA | NA | What are the wooden clappers called that Spanish dancers hold in their hands? | Castanets | .69 | .12 | .04 | .15 | .00 | 12060 | 25695 | 9706 | 16126 |  |
| C | 51 | NA | NA | What was the name of the vehicle used to carry and cook food on cattle drives? | Chuckwagon | .67 | .00 | .01 | .31 | .00 | 12581 |  | 12289 | 29955 |  |
| B | 53 | NA | NA | What are people who make maps called? | Cartographers | .67 | .06 | .24 | .04 | .00 | 13531 | 17546 | 8952 | 9951 |  |
|  |  |  |  |  |  | CR (proportion of responses) | | | | | CR (response times) | | | | |
| Set | CR Rank | T. et al. rank | N & N rank | General Knowledge Question | Correct Answer | Correct | DR | DK | CE | OE | Correct | DR | DK | CE | OE |
| B | 53 | NA | NA | What is the unit that marks sound intensity? | Decibel | .67 | .07 | .13 | .13 | .00 | 14154 | 28216 | 10407 | 13674 |  |
| B | 53 | NA | NA | What is the front section of a boat? | Bow | .67 | .07 | .02 | .24 | .00 | 9888 | 11350 | 2986 | 7807 |  |
| C | 55.5 | NA | NA | Humans are classified as what species? | Sapiens | .66 | .01 | .00 | .33 | .00 | 12219 | 21122 |  | 15264 |  |
| C | 55.5 | NA | NA | What is the name of the palace in London in which the Monarch of England resides? | Buckingham | .66 | .13 | .01 | .19 | .00 | 13549 | 24646 | 9408 | 23046 |  |
| B | 61.5 | NA | NA | What is the resort city on the French Riviera between Nice and St. Tropez where a famous film festival is held? | Cannes | .65 | .04 | .22 | .09 | .00 | 16071 | 10514 | 10032 | 13112 |  |
| B | 61.5 | NA | NA | What do you call a feeling of resentment, often at some fancied slight or insult? (usually follows the verb "take") | Umbrage | .24 | .00 | .19 | .56 | .02 | 21661 | 17694 | 34802 | 17455 | 1050 |
| B | 61.5 | NA | NA | What is the name of a mixture of dried spices and flowers used for perfuming a room? | Potpourri | .65 | .09 | .06 | .19 | .02 | 27416 | 12405 | 8202 | 15423 | 1607 |
| B | 61.5 | NA | NA | What is the order of lower mammals including kangaroos and opossums which carry their young in an abdominal pouch? | Marsupialia | .65 | .11 | .22 | .02 | .00 | 21352 | 11133 | 10723 | 1668 |  |
| B | 61.5 | NA | NA | Who is the artist that painted everyday objects such as soup cans? | Warhol | .65 | .11 | .20 | .04 | .00 | 18177 | 44745 | 6601 | 8508 |  |
| B | 61.5 | NA | NA | What do you call a plant material such as straw used as a roofing material for a house? | Thatch | .65 | .04 | .20 | .11 | .00 | 11185 | 6252 | 12353 | 14477 |  |
| B | 61.5 | NA | NA | What is the name of the unrhymed verse form of Japanese origin having three lines containing usually 5, 7, and 5 syllables? | Haiku | .65 | .04 | .28 | .04 | .00 | 17762 | 7252 | 12685 | 7920 |  |
|  |  |  |  |  |  | CR (proportion of responses) | | | | | CR (response times) | | | | |
| Set | CR Rank | T. et al. rank | N & N rank | General Knowledge Question | Correct Answer | Correct | DR | DK | CE | OE | Correct | DR | DK | CE | OE |
| B | 61.5 | NA | NA | What is the capital of Thailand? | Bangkok | .65 | .06 | .20 | .07 | .02 | 11171 | 13498 | 5702 | 13651 | 2790 |
| B | 61.5 | NA | NA | Who is the oldest sister in "The Brady Bunch"? | Marcia | .65 | .15 | .15 | .06 | .00 | 10644 | 9633 | 5459 | 12208 |  |
| B | 61.5 | NA | NA | What word means to divide an area into electoral district in order to give special advantage to one political party? | Gerrymander | .65 | .07 | .22 | .06 | .00 | 18490 | 22447 | 16468 | 16391 |  |
| C | 68.5 | NA | NA | What is the fin on the back of a fish called? | Dorsal | .64 | .07 | .16 | .10 | .01 | 9555 | 19907 | 14615 | 23654 | 35858 |
| C | 68.5 | NA | NA | What mammal has armor-like bony plates as its most distinguishing feature? | Armadillo | .64 | .07 | .09 | .19 | .00 | 13987 | 27685 | 13863 | 29768 |  |
| C | 68.5 | NA | NA | What did the Seven Dwarves do for a living? | Mining | .64 | .09 | .10 | .16 | .00 | 10040 | 10197 | 13572 | 17626 |  |
| C | 68.5 | NA | NA | What river separates Washington D.C. from Virginia? | Potomac | .64 | .16 | .03 | .15 | .01 | 10585 | 27007 | 22975 | 16876 | 55723 |
| B | 71 | NA | NA | What do you call a member of a volunteer group organized to suppress and punish crime (as when legal processes seem inadequate)? | Vigilante | .63 | .07 | .15 | .15 | .00 | 23342 | 12417 | 13172 | 15686 |  |
| C | 74 | NA | NA | What instrument is used to measure earthquakes? | Seismograph | .63 | .10 | .01 | .25 | .00 | 17372 | 18051 | 6303 | 22728 |  |
| C | 74 | NA | NA | What was the land where Puff the Magic Dragon lives? | Honalee | .63 | .10 | .04 | .21 | .01 | 14819 | 17209 | 14188 | 20557 | 40858 |
| C | 74 | NA | NA | What sailor's disease resulted from a deficiency in vitamin C? | Scurvy | .63 | .06 | .06 | .25 | .00 | 9952 | 18811 | 14685 | 21058 |  |
| C | 74 | NA | NA | What is the technical term for the phenomenon commonly known as the Northern Lights? | Aurora Borealis | .63 | .09 | .15 | .12 | .01 | 18364 | 25828 | 11650 | 56979 | 998 |
|  |  |  |  |  |  |  |  |  |  |  |  |  |  |  |  |
|  |  |  |  |  |  | CR (proportion of responses) | | | | | CR (response times) | | | | |
| Set | CR Rank | T. et al. rank | N & N rank | General Knowledge Question | Correct Answer | Correct | DR | DK | CE | OE | Correct | DR | DK | CE | OE |
| C | 74 | NA | NA | What type of paper is commonly used as an acid-base indicator in chemistry classes? | Litmus | .63 | .07 | .19 | .10 | .00 | 12999 | 21669 | 14651 | 15841 |  |
| C | 77 | NA | NA | What is the name of the short sword fastened to the end of a musket or rifle? | Bayonet | .61 | .07 | .12 | .18 | .01 | 12494 | 21794 | 18927 | 19045 | 12927 |
| B | 78 | NA | NA | Where is the Amazon rainforest located? | Brazil | .61 | .02 | .06 | .31 | .00 | 12450 | 32661 | 8818 | 17521 |  |
| C | 80 | NA | NA | What device is used to measure levels of radioactivity? | Geiger Counter | .60 | .21 | .09 | .09 | .01 | 12760 | 17459 | 26642 | 21462 | 61341 |
| C | 80 | NA | NA | Who was the magician and prophet from King Arthur's era? | Merlin | .60 | .21 | .12 | .04 | .03 | 12250 | 21436 | 10224 | 41090 | 12911 |
| C | 80 | NA | NA | What was the name of the disorder depicted by Dustin Hoffman's character in the movie 'Rainman' otherwise known as Idiot Savantism? | Autism | .60 | .13 | .15 | .10 | .01 | 22682 | 28438 | 22400 | 42181 | 29355 |
| A | 82.5 | NA | NA | What does VoIP stand for? | Voice over Internet Protocol | .60 | .02 | .26 | .12 | .00 | 23624 | 10021 | 8741 | 23351 |  |
| A | 82.5 | NA | NA | Who defeated Pres. Harry Truman in the 1948 election according to an infamous Chicago Tribune headline? | Thomas Dewey | .60 | .16 | .21 | .04 | .00 | 18543 | 13155 | 9920 | 28748 |  |
| B | 86 | NA | NA | What is the name of the migratory grasshopper that travels in vast swarms and strips areas passed of all vegetation? | Locust | .59 | .09 | .17 | .15 | .00 | 11649 | 25911 | 14144 | 12684 |  |
| B | 86 | NA | NA | What city is the capital of Iceland? | Reykjavik | .59 | .09 | .24 | .06 | .02 | 15816 | 17614 | 8555 | 7203 | 2382 |
|  |  |  |  |  |  |  |  |  |  |  |  |  |  |  |  |
|  |  |  |  |  |  | CR (proportion of responses) | | | | | CR (response times) | | | | |
| Set | CR Rank | T. et al. rank | N & N rank | General Knowledge Question | Correct Answer | Correct | DR | DK | CE | OE | Correct | DR | DK | CE | OE |
| B | 86 | NA | NA | Who was the first female Supreme Court justice? | O'Connor | .59 | .13 | .19 | .09 | .00 | 27108 | 23430 | 5649 | 16562 |  |
| B | 86 | NA | NA | Who was the composer who worked with deafness? | Beethoven | .59 | .02 | .17 | .22 | .00 | 19775 | 6175 | 19919 | 9748 |  |
| B | 86 | NA | NA | What is the last name of the man who assassinated Robert Kennedy? | Sirhan | .59 | .19 | .07 | .15 | .00 | 17317 | 13717 | 9523 | 15723 |  |
| C | 89 | NA | NA | What is a community of ants called? | Colony | .58 | .03 | .04 | .33 | .01 | 8538 | 13121 | 13066 | 17543 | 18102 |
| B | 91.5 | NA | NA | What is the last name of the actor who played Perry Mason on TV? | Burr | .57 | .22 | .15 | .06 | .00 | 13623 | 13016 | 5072 | 55740 |  |
| B | 91.5 | NA | NA | What do you call a stone building (often found in cemetery) with places for entombment of the dead above ground? | Mausoleum | .57 | .09 | .09 | .24 | .00 | 23320 | 52414 | 23210 | 15755 |  |
| B | 91.5 | NA | NA | What is the former name of Istanbul, which was used when the city was occupied by Christians? | Constantinople | .57 | .07 | .30 | .06 | .00 | 22830 | 32674 | 7001 | 19019 |  |
| B | 91.5 | NA | NA | What was the last name of Lucille Ball's first husband, who starred with her on "I Love Lucy"? | Arnaz | .57 | .04 | .02 | .35 | .02 | 12671 | 43324 | 8677 | 12491 | 2209 |
| C | 96 | NA | NA | What is the name of the three leaf clover which is the emblem of Ireland? | Shamrock | .57 | .15 | .22 | .04 | .01 | 12728 | 28581 | 18578 | 8360 | 13480 |
| C | 96 | NA | NA | What was the name of the infamous American traitor in the Revolutionary War? | Arnold | .57 | .21 | .01 | .21 | .00 | 17009 | 30200 | 16031 | 89377 |  |
| C | 96 | NA | NA | What is a group of geese called? | Gaggle | .57 | .03 | .01 | .37 | .01 | 7744 | 23742 | 7498 | 11176 | 11964 |
| C | 96 | NA | NA | What is a young female horse called? | Filly | .57 | .06 | .00 | .37 | .00 | 9430 | 51608 |  | 15419 |  |
|  |  |  |  |  |  |  |  |  |  |  |  |  |  |  |  |
|  |  |  |  |  |  |  |  |  |  |  |  |  |  |  |  |
|  |  |  |  |  |  | CR (proportion of responses) | | | | | CR (response times) | | | | |
| Set | CR Rank | T. et al. rank | N & N rank | General Knowledge Question | Correct Answer | Correct | DR | DK | CE | OE | Correct | DR | DK | CE | OE |
| C | 96 | NA | NA | What is the name of the disease of the liver caused by abnormally high levels of bile in the blood that gives the skin a yellow color? | Jaundice | .57 | .09 | .00 | .34 | .00 | 15856 | 39291 |  | 28184 |  |
| D | 99 | 190 | 181 | What is the last name of the man who began the Reformation in Germany? | Luther | .56 | .09 | .27 | .08 | .00 | 12457 | 28706 | 9909 | 11438 |  |
| B | 101 | NA | NA | What is the name of the islands off the coast of Ecuador that Darwin visited to study unique species of birds and animals? | Galapagos | .56 | .15 | .22 | .07 | .00 | 26153 | 17483 | 7989 | 10490 |  |
| B | 101 | NA | NA | What is the word that means to cause to explode or to set off, for example: a bomb? | Detonate | .56 | .06 | .06 | .33 | .00 | 11987 | 21066 | 10114 | 15789 |  |
| B | 101 | NA | NA | What is the orbiting particle of an atom? | Electron | .56 | .07 | .20 | .17 | .00 | 18131 | 11170 | 4067 | 36049 |  |
| C | 104 | NA | NA | What was the last name of the female mouseketeer who later appeared in commercials for peanut butter? | Funicello | .55 | .09 | .19 | .15 | .01 | 14773 | 15804 | 15241 | 17899 | 61201 |
| C | 104 | NA | NA | What is the famous prehistoric structure situated on Salisbury Plain England? | Stonehenge | .55 | .24 | .12 | .06 | .03 | 15418 | 30043 | 8442 | 63415 | 37594 |
| C | 104 | NA | NA | What seasonal South Asian wind is characterized by heavy rains? | Monsoon | .55 | .18 | .09 | .18 | .00 | 14005 | 33728 | 14703 | 14906 |  |
| C | 107 | NA | NA | What is the name of the legendary race of female warriors? | Amazons | .54 | .18 | .15 | .12 | .01 | 10165 | 21414 | 7737 | 35494 | 6365 |
| C | 107 | NA | NA | What is the wand called which is held in the hand as a symbol of regal or imperial power? | Scepter | .54 | .13 | .13 | .19 | .00 | 14527 | 19472 | 20402 | 26568 |  |
|  |  |  |  |  |  |  |  |  |  |  |  |  |  |  |  |
|  |  |  |  |  |  | CR (proportion of responses) | | | | | CR (response times) | | | | |
| Set | CR Rank | T. et al. rank | N & N rank | General Knowledge Question | Correct Answer | Correct | DR | DK | CE | OE | Correct | DR | DK | CE | OE |
| C | 107 | NA | NA | What was the name of the medieval code of honor practiced by knights that among other things elevated the position of women? | Chivalry | .54 | .07 | .30 | .07 | .01 | 18371 | 17359 | 15311 | 28334 | 17338 |
| B | 109.5 | NA | NA | Who said the phrase, "To be or not to be"? | Hamlet | .54 | .00 | .04 | .43 | .00 | 17050 |  | 23751 | 13586 |  |
| B | 109.5 | NA | NA | What is a group of lines in a poem called? | Stanza | .54 | .06 | .11 | .30 | .00 | 8950 | 27119 | 6864 | 12630 |  |
| D | 111.5 | 168 | 221 | Over which river is the George Washington Bridge? | Hudson | .53 | .11 | .09 | .27 | .00 | 12472 | 18206 | 16185 | 20233 |  |
| D | 111.5 | 179 | 183 | What is the last name of the actor who received the Best Actor award for the movie "On The Waterfront"? | Brando | .53 | .18 | .24 | .05 | .00 | 15552 | 34635 | 9566 | 27133 |  |
| C | 114 | NA | NA | What was the location of George Washington's encampment where his men suffered every conceivable hardship from 1777-1778? | Valley Forge | .52 | .21 | .06 | .21 | .00 | 16050 | 23017 | 13499 | 35336 |  |
| C | 114 | NA | NA | What was the last name of the nearly blind impressionist artist who painted a series of mural-sized pictures of water lilies near the end of his life? | Monet | .52 | .06 | .25 | .16 | .00 | 18685 | 38750 | 14012 | 22168 |  |
| C | 114 | NA | NA | What dwarf kills himself when a young bride guesses his highly unusual name? | Rumpelstiltskin | .52 | .04 | .36 | .04 | .03 | 23723 | 26134 | 12035 | 17087 | 15174 |
| B | 117 | NA | NA | What word means to bleed heavily or uncontrollably? | Hemorrhage | .52 | .15 | .19 | .15 | .00 | 15296 | 36515 | 13950 | 18404 |  |
|  |  |  |  |  |  |  |  |  |  |  |  |  |  |  |  |
|  |  |  |  |  |  | CR (proportion of responses) | | | | | CR (response times) | | | | |
| Set | CR Rank | T. et al. rank | N & N rank | General Knowledge Question | Correct Answer | Correct | DR | DK | CE | OE | Correct | DR | DK | CE | OE |
| B | 117 | NA | NA | What is the name of the strait between Alaska and Siberia? | Bering | .52 | .15 | .22 | .11 | .00 | 10217 | 19904 | 7177 | 10296 |  |
| B | 117 | NA | NA | What is the last name of the current mayor of New York City? | De Blasio | .52 | .13 | .19 | .17 | .00 | 19371 | 6967 | 14276 | 12934 |  |
| C | 119.5 | NA | NA | What beetle was held sacred by ancient Egyptians? | Scarab | .51 | .07 | .27 | .15 | .00 | 10050 | 15124 | 10559 | 15569 |  |
| C | 119.5 | NA | NA | What river in Hades does Charon ferry dead souls across? | Styx | .51 | .15 | .21 | .13 | .00 | 9193 | 15805 | 10766 | 23811 |  |
| C | 121 | NA | NA | Who shot an apple off of his son's head in the 14th century? | William Tell | .49 | .25 | .03 | .21 | .01 | 12220 | 26229 | 15688 | 14664 | 51468 |
| B | 122.5 | NA | NA | What is the last name of the author of Little Women? | Alcott | .48 | .20 | .17 | .15 | .00 | 37061 | 8259 | 5191 | 8383 |  |
| B | 122.5 | NA | NA | What is last name of the woman who wrote Gone With the Wind? | Mitchell | .48 | .19 | .26 | .07 | .00 | 16023 | 16498 | 7662 | 13867 |  |
| C | 124 | NA | NA | What is the last name of the author of 'The Hobbit'? | Tolkien | .48 | .25 | .22 | .03 | .01 | 13096 | 17086 | 8675 | 9164 | 6040 |
| A | 125 | NA | NA | What did the Wright Brothers do before inventing an aircraft? | Built bicycles | .47 | .16 | .25 | .12 | .00 | 14550 | 11813 | 8266 | 19067 |  |
| B | 128 | NA | NA | What is the last name of the actress who played Gloria on TV's "All in the Family"? | Struthers | .46 | .26 | .19 | .09 | .00 | 18179 | 15717 | 9929 | 14871 |  |
| B | 128 | NA | NA | What is the last name of the actor who starred in both the Broadway production and the film, "A Streetcar Named Desire"? | Brando | .46 | .11 | .35 | .07 | .00 | 15059 | 14938 | 12973 | 31913 |  |
|  |  |  |  |  |  |  |  |  |  |  |  |  |  |  |  |
|  |  |  |  |  |  | CR (proportion of responses) | | | | | CR (response times) | | | | |
| Set | CR Rank | T. et al. rank | N & N rank | General Knowledge Question | Correct Answer | Correct | DR | DK | CE | OE | Correct | DR | DK | CE | OE |
| B | 128 | NA | NA | What is the last name of the Romanian gymnast who scored 7 perfect "10's" and won 3 gold medals in the 1976 Olympics? | Comaneci | .46 | .17 | .19 | .17 | .02 | 20441 | 21253 | 6408 | 48542 | 1337 |
| B | 128 | NA | NA | What do you call a habitual spasmodic motion or twitching of particular muscles, especially in the face? | Tic | .46 | .06 | .19 | .30 | .00 | 11627 | 13298 | 7301 | 18202 |  |
| B | 128 | NA | NA | What is the river that flows north from Switzerland to Holland and forms the border between Germany and France? | Rhine | .46 | .06 | .33 | .13 | .02 | 16345 | 13932 | 10680 | 29014 | 1451 |
| C | 131 | NA | NA | What was the harp-like instrument used in ancient Greece to accompany singing and recitation? | Lyre | .46 | .12 | .12 | .30 | .00 | 8399 | 26891 | 13863 | 14945 |  |
| C | 134 | NA | NA | What was the name of the zeppelin, blimp, that exploded in Lake Hurst, New Jersey in 1937? | Hindenburg | .45 | .24 | .18 | .12 | .01 | 19734 | 25608 | 9646 | 30901 | 8585 |
| C | 134 | NA | NA | What is a male witch called? | Warlock | .45 | .13 | .22 | .18 | .01 | 7696 | 20333 | 15558 | 14827 | 6692 |
| C | 134 | NA | NA | What is the first name of the school teacher who was chased by the headless horseman in 'The Legend of Sleepy Hollow'? | Ichabod | .45 | .31 | .13 | .09 | .01 | 14820 | 20651 | 16466 | 19242 | 26015 |
| C | 134 | NA | NA | What is the last name of the author of 'Uncle Tom's Cabin'? | Stowe | .45 | .25 | .07 | .21 | .01 | 10921 | 19733 | 15840 | 15033 | 9981 |
|  |  |  |  |  |  |  |  |  |  |  |  |  |  |  |  |
|  |  |  |  |  |  | CR (proportion of responses) | | | | | CR (response times) | | | | |
| Set | CR Rank | T. et al. rank | N & N rank | General Knowledge Question | Correct Answer | Correct | DR | DK | CE | OE | Correct | DR | DK | CE | OE |
| C | 134 | NA | NA | What was the last name of Scrooge's dead partner in Dickens' 'A Christmas Carol?' | Marley | .45 | .33 | .07 | .12 | .03 | 10754 | 18808 | 9492 | 38353 | 19456 |
| B | 138 | NA | NA | What is the term for when three musical notes are played together? | Chord | .44 | .06 | .37 | .13 | .00 | 10404 | 10520 | 11631 | 14153 |  |
| B | 138 | NA | NA | What do you call the three periods(…) used to indicate an omission or a pause? | Ellipsis | .44 | .17 | .31 | .06 | .02 | 16454 | 15037 | 18928 | 31341 | 3045 |
| B | 138 | NA | NA | What word would means having a healthy reddish color? | Ruddy | .44 | .06 | .17 | .33 | .00 | 9958 | 18168 | 10961 | 13975 |  |
| D | 140 | 175 | 192 | What is the last name of the author who wrote the Sherlock Holmes stories? | Doyle | .44 | .27 | .23 | .06 | .00 | 13247 | 16687 | 10180 | 18556 |  |
| A | 141.5 | NA | NA | What game was Deep Blue skilled at? | Chess | .44 | .02 | .49 | .05 | .00 | 11453 | 6328 | 10317 | 9464 |  |
| A | 141.5 | NA | NA | Who was Time Magazine's 1938 "Man of the Year"? | Adolph Hitler | .44 | .02 | .44 | .09 | .02 | 12692 | 7598 | 6612 | 11766 | 4510 |
| C | 144 | NA | NA | Who was the fictional character who married his mother and blinded himself? | Oedipus | .43 | .10 | .30 | .13 | .03 | 15334 | 27306 | 10883 | 19234 | 16339 |
| C | 144 | NA | NA | What was the name of the German Secret State Police? | Gestapo | .43 | .16 | .09 | .31 | .00 | 14481 | 18990 | 12145 | 18042 |  |
| C | 144 | NA | NA | What peace treaty ended World War I? | Versailles | .43 | .24 | .10 | .21 | .01 | 21092 | 11662 | 8258 | 13381 | 18428 |
| B | 146 | NA | NA | Which bone does the colloquial term "jawbone" refer to? | Mandible | .43 | .04 | .35 | .19 | .00 | 15843 | 5788 | 22563 | 16024 |  |
| A | 147 | NA | NA | Which Aerosmith song was re-made by Run D.M.C.? | Walk this Way | .42 | .05 | .44 | .09 | .00 | 15422 | 17090 | 8209 | 23847 |  |
| C | 148 | NA | NA | What is the name for the legendary Egyptian sculpture which asked people a famous riddle? | Sphinx | .42 | .15 | .33 | .09 | .01 | 13569 | 13821 | 13950 | 16995 | 28409 |
|  |  |  |  |  |  | CR (proportion of responses) | | | | | CR (response times) | | | | |
| Set | CR Rank | T. et al. rank | N & N rank | General Knowledge Question | Correct Answer | Correct | DR | DK | CE | OE | Correct | DR | DK | CE | OE |
| D | 150 | 191 | 211 | What is the capital of Finland? | Helsinki | .41 | .20 | .30 | .09 | .00 | 8028 | 20694 | 11643 | 14970 |  |
| D | 150 | 202 | 218 | What is the capital of Canada? | Ottawa | .41 | .09 | .08 | .42 | .00 | 10504 | 15453 | 13054 | 21362 |  |
| D | 150 | 230 | 219 | What is the last name of the inventor of the wireless radio? | Marconi | .41 | .21 | .18 | .20 | .00 | 13999 | 25400 | 11541 | 14638 |  |
| B | 152 | NA | NA | What is the largest city and capital of Kenya? | Nairobi | .41 | .06 | .46 | .06 | .02 | 18053 | 16362 | 6323 | 12371 | 1114 |
| D | 153.5 | 173 | 161 | Who was the most famous Greek doctor? | Hippocrates | .39 | .12 | .21 | .27 | .00 | 19515 | 20391 | 9241 | 18776 |  |
| D | 153.5 | 183 | 193 | What is the last name of the husband-wife spies who were electrocuted in 1951 for passing atomic secrets to Russia? | Rosenberg | .39 | .27 | .15 | .18 | .00 | 17697 | 23073 | 12346 | 34039 |  |
| B | 155 | NA | NA | What is the gas that forms dry ice when frozen? | Carbon dioxide | .39 | .13 | .26 | .22 | .00 | 17463 | 15328 | 14612 | 12558 |  |
| C | 156.5 | NA | NA | What was the last name of the explorer who tramped through what is now Florida looking for the fountain of youth? | Ponce De Leon | .39 | .36 | .04 | .19 | .01 | 17279 | 26548 | 22413 | 23120 | 83110 |
| C | 156.5 | NA | NA | What is the last name of the author of 'Call of the Wild'? | London | .39 | .27 | .13 | .21 | .00 | 7466 | 20181 | 8631 | 16361 |  |
| D | 158.5 | 157 | 156 | What kind of poison did Socrates take as his execution? | Hemlock | .38 | .18 | .18 | .26 | .00 | 9557 | 23021 | 11658 | 21520 |  |
| D | 158.5 | 216 | 230 | What is the last name of the woman who founded the American Red Cross? | Barton | .38 | .24 | .17 | .21 | .00 | 13056 | 29249 | 14914 | 25207 |  |
| C | 160.5 | NA | NA | Who was the person who visited the Lilliputians? | Gulliver | .37 | .22 | .16 | .21 | .03 | 13245 | 17662 | 7730 | 22453 | 11182 |
| C | 160.5 | NA | NA | Who was Robin Hood's greatest enemy? | Sheriff of Nottingham | .37 | .27 | .09 | .27 | .00 | 16435 | 12692 | 8626 | 19842 |  |
| B | 166 | NA | NA | What is the navigation instrument used at sea to plot positioning by the stars? | Sextant | .37 | .20 | .17 | .24 | .02 | 7849 | 31003 | 9990 | 10885 | 2965 |
|  |  |  |  |  |  | CR (proportion of responses) | | | | | CR (response times) | | | | |
| Set | CR Rank | T. et al. rank | N & N rank | General Knowledge Question | Correct Answer | Correct | DR | DK | CE | OE | Correct | DR | DK | CE | OE |
| B | 166 | NA | NA | What do you call a word or sentence that reads the same backward or forward, such as "Madam, I'm Adam"? | Palindrome | .37 | .28 | .24 | .09 | .02 | 23654 | 14017 | 9705 | 40702 | 2251 |
| B | 166 | NA | NA | Which body of water separates Central and South America? | Panama Canal | .37 | .09 | .33 | .19 | .02 | 20127 | 7904 | 20838 | 19086 | 1480 |
| B | 166 | NA | NA | What word means to cut or chop (food) into very small pieces? | Mince | .37 | .00 | .13 | .50 | .00 | 9414 |  | 17944 | 8958 |  |
| B | 166 | NA | NA | What was the capital of Czechoslovakia? | Prague | .37 | .17 | .43 | .04 | .00 | 13652 | 12626 | 7099 | 24787 |  |
| B | 166 | NA | NA | What is the mixture of two or more metallic elements? | Alloy | .37 | .07 | .33 | .22 | .00 | 13970 | 21799 | 11463 | 12449 |  |
| B | 166 | NA | NA | What do you call the leather band formerly used for sharpening an old-fashioned razor? | Strop | .37 | .04 | .17 | .43 | .00 | 12720 | 16622 | 8616 | 12371 |  |
| B | 166 | NA | NA | What is the colloquial term for patella? | Kneecap | .37 | .04 | .44 | .15 | .00 | 12896 | 22050 | 9169 | 10984 |  |
| B | 166 | NA | NA | What is the name of the hardwood of an (Asian) Indian timber tree often used for furniture? | Teak | .37 | .04 | .26 | .33 | .00 | 16059 | 13892 | 10839 | 20636 |  |
| A | 171.5 | NA | NA | Who was Elton John's chief lyricist? | Bernie Taupin | .37 | .05 | .49 | .09 | .00 | 22554 | 11281 | 5855 | 13945 |  |
| A | 171.5 | NA | NA | Which serial killer called himself "Son of Sam"? | David Berkowitz | .37 | .37 | .11 | .16 | .00 | 20828 | 12289 | 25257 | 14242 |  |
| D | 173.5 | 182 | 191 | Who is known as "The Father of Geometry"? | Euclid | .36 | .08 | .41 | .15 | .00 | 10631 | 17897 | 8111 | 19240 |  |
| D | 173.5 | 260 | 186 | What was the name of the Union ironclad ship that fought the Confederate ironclad Merrimack? | Monitor | .36 | .15 | .30 | .18 | .00 | 12552 | 18434 | 13227 | 20739 |  |
| C | 175 | NA | NA | In 'Romeo and Juliet' who were the Montagues feuding with? | Capulets | .36 | .31 | .21 | .09 | .03 | 11248 | 17099 | 10769 | 17569 | 13876 |
|  |  |  |  |  |  |  | | | | |  | | | | |
|  |  |  |  |  |  | CR (proportion of responses) | | | | | CR (response times) | | | | |
| Set | CR Rank | T. et al. rank | N & N rank | General Knowledge Question | Correct Answer | Correct | DR | DK | CE | OE | Correct | DR | DK | CE | OE |
| B | 177.5 | NA | NA | What is the last name of the actor who starred in "Hart to Hart" and "Switch" on TV and also in many movies? | Wagner | .35 | .30 | .26 | .09 | .00 | 19704 | 26555 | 11739 | 17514 |  |
| B | 177.5 | NA | NA | What do you call a person who collects and studies stamps? | Philatelist | .35 | .19 | .30 | .17 | .00 | 20061 | 12167 | 10722 | 9479 |  |
| B | 177.5 | NA | NA | What is the name of the river that runs through Rome? | Tiber | .35 | .15 | .35 | .15 | .00 | 18322 | 29232 | 9246 | 9626 |  |
| D | 177.5 | 265 | 222 | What is the name of the mountain range that separates Asia from Europe? | Ural | .35 | .15 | .13 | .59 | .00 | 10587 | 14281 | 7748 | 21818 |  |
| A | 182.5 | NA | NA | What was the 2001 event that led to the bankruptcy of a U.S. energy company and resulted in the dissolution of Arthur Anderson? | The Enron Scandal | .35 | .12 | .37 | .16 | .00 | 29993 | 20232 | 15233 | 29509 |  |
| A | 182.5 | NA | NA | Whose autobiography is titled "Lady Sings the Blues"? | Billie Holiday | .35 | .18 | .23 | .25 | .00 | 19102 | 15308 | 7420 | 17408 |  |
| A | 182.5 | NA | NA | What is the name of the Viking who discovered Greenland? | Erik the Red | .35 | .05 | .39 | .21 | .00 | 20313 | 7873 | 9053 | 16546 |  |
| A | 182.5 | NA | NA | What was Bob Dylan's birth name? | Robert Zimmerman | .35 | .25 | .30 | .11 | .00 | 17191 | 5963 | 4733 | 17168 |  |
| A | 182.5 | NA | NA | Where was the 1939 World's Fair held? | New York City | .35 | .04 | .25 | .37 | .00 | 14279 | 12441 | 7100 | 9086 |  |
| A | 182.5 | NA | NA | What did the irascible Ted Williams do in the final at bat of his 19-year major-league career? | Hit a home run | .35 | .02 | .46 | .18 | .00 | 19439 | 8705 | 10985 | 14000 |  |
| C | 186 | NA | NA | On top of which mountain was Moses given the Ten Commandments? | Sinai | .34 | .18 | .09 | .36 | .03 | 12419 | 21276 | 11509 | 22839 | 7730 |
| B | 188.5 | NA | NA | What is the last name of the Cuban leader that Castro overthrew? | Batista | .33 | .20 | .35 | .11 | .00 | 21911 | 14789 | 10694 | 10976 |  |
|  |  |  |  |  |  |  |  |  |  |  |  |  |  |  |  |
|  |  |  |  |  |  | CR (proportion of responses) | | | | | CR (response times) | | | | |
| Set | CR Rank | T. et al. rank | N & N rank | General Knowledge Question | Correct Answer | Correct | DR | DK | CE | OE | Correct | DR | DK | CE | OE |
| B | 188.5 | NA | NA | What is the name of the fountain in Rome into which coins are thrown for good luck? | Trevi | .33 | .19 | .33 | .15 | .00 | 13752 | 9282 | 9785 | 21725 |  |
| B | 188.5 | NA | NA | What was the English settlement that vanished in the late 1580s? | Roanoke | .33 | .15 | .31 | .20 | .00 | 26206 | 14714 | 9510 | 11927 |  |
| B | 188.5 | NA | NA | What do you call a moderately fast gait of a horse in which the legs move in diagonal pairs? | Trot | .33 | .04 | .17 | .44 | .02 | 11539 | 8628 | 8463 | 16664 | 17029 |
| C | 192 | NA | NA | What is the term given to pure spiritual love which is devoid of carnal desires between members of opposite sexes? | Platonic | .33 | .16 | .18 | .33 | .00 | 21466 | 40394 | 24656 | 21349 |  |
| C | 192 | NA | NA | What was the disease known as the 'Black Plague'? | Bubonic | .33 | .18 | .09 | .39 | .01 | 15796 | 30330 | 12067 | 23670 | 29883 |
| C | 192 | NA | NA | What was the last name of the person known as the 'desert fox' | Rommel | .33 | .25 | .21 | .19 | .01 | 9243 | 18926 | 9501 | 22745 | 9786 |
| D | 195.5 | 199 | 209 | What is the last name of the first person to climb Mount Everest? | Hillary | .32 | .30 | .33 | .05 | .00 | 14459 | 15809 | 9437 | 23883 |  |
| D | 195.5 | 223 | 237 | What is the last name of the British admiral who won the Battle of Trafalgar? | Nelson | .32 | .18 | .47 | .03 | .00 | 8790 | 33900 | 8005 | 13392 |  |
| D | 195.5 | 235 | 210 | What is the last name of the French author who wrote “The Stranger”? | Camus | .32 | .05 | .61 | .03 | .00 | 8231 | 24239 | 6837 | 10414 |  |
| D | 195.5 | 267 | 229 | What is the last name of the man who created the comic strip "Li'l Abner"? | Capp | .32 | .21 | .44 | .03 | .00 | 8744 | 16972 | 9153 | 12085 |  |
| A | 198 | NA | NA | To which continent is the yam native? | Africa | .32 | .02 | .21 | .46 | .00 | 12121 | 9624 | 7457 | 14907 |  |
| B | 199.5 | NA | NA | What word means to raise into position by means of a pulley? | Hoist | .31 | .13 | .35 | .20 | .00 | 13801 | 24273 | 16542 | 14042 |  |
|  |  |  |  |  |  |  |  |  |  |  |  |  |  |  |  |
|  |  |  |  |  |  | CR (proportion of responses) | | | | | CR (response times) | | | | |
| Set | CR Rank | T. et al. rank | N & N rank | General Knowledge Question | Correct Answer | Correct | DR | DK | CE | OE | Correct | DR | DK | CE | OE |
| B | 199.5 | NA | NA | What is the old name of Taiwan? | Formosa | .31 | .20 | .31 | .17 | .00 | 15419 | 23829 | 10995 | 11200 |  |
| C | 201 | NA | NA | What was the creature killed by Theseus which had the body of a man and the head of a bull? | Minotaur | .31 | .22 | .30 | .15 | .01 | 15216 | 16539 | 14147 | 11044 | 16205 |
| D | 203 | 153 | 203 | In which city is Michelangelo's statue of David located? | Florence | .30 | .02 | .02 | .67 | .00 | 11882 | 21675 | 5976 | 14103 |  |
| D | 203 | 169 | 189 | What is the last name of the man who invented dynamite? | Nobel | .30 | .09 | .56 | .05 | .00 | 14272 | 19273 | 7005 | 21708 |  |
| D | 203 | 259 | 180 | What is John Kenneth Galbraith's profession? | Economist | .30 | .11 | .24 | .35 | .00 | 9809 | 11568 | 10200 | 15109 |  |
| C | 205 | NA | NA | Who was the bouncy and egotistical friend of Christopher Robin? | Tigger | .30 | .07 | .18 | .42 | .03 | 18875 | 19530 | 10810 | 21063 | 12321 |
| A | 207 | NA | NA | What is a network designed to allow communication within an organization? | An intranet | .30 | .05 | .40 | .25 | .00 | 22483 | 13167 | 13777 | 19969 |  |
| A | 207 | NA | NA | What was invented by Wilhelm Rontgen in 1895? | X-ray machine | .30 | .02 | .58 | .11 | .00 | 11992 | 5932 | 7946 | 13628 |  |
| A | 207 | NA | NA | Who is the national poet of Scotland? | Robert Burns | .30 | .07 | .51 | .12 | .00 | 14158 | 10172 | 5262 | 17142 |  |
| B | 209.5 | NA | NA | What do you call a sharp and sudden pain in the side? | Stitch | .30 | .04 | .31 | .35 | .00 | 8820 | 38043 | 11990 | 29205 |  |
| B | 209.5 | NA | NA | What is the metric for gemstone quality? | Carat | .30 | .09 | .37 | .24 | .00 | 18197 | 51490 | 9185 | 40476 |  |
| D | 211 | 196 | 176 | What was the last name of the composer of the "Maple Leaf Rag"? | Joplin | .29 | .06 | .58 | .05 | .03 | 9199 | 23910 | 6967 | 17193 | 5726 |
| C | 212 | NA | NA | What is the long process by which a dead organism turns to stone? | Petrification | .28 | .06 | .07 | .57 | .01 | 21869 | 13767 | 40346 | 21935 | 158735 |
|  |  |  |  |  |  |  |  |  |  |  |  |  |  |  |  |
|  |  |  |  |  |  | CR (proportion of responses) | | | | | CR (response times) | | | | |
| Set | CR Rank | T. et al. rank | N & N rank | General Knowledge Question | Correct Answer | Correct | DR | DK | CE | OE | Correct | DR | DK | CE | OE |
| A | 214 | NA | NA | What was the first feature-length motion picture with sound? | The Jazz Singer | .28 | .09 | .44 | .19 | .00 | 14652 | 14498 | 6868 | 18699 |  |
| A | 214 | NA | NA | Which sport has the highest amount of doping? | Cycling | .28 | .00 | .25 | .47 | .00 | 19921 |  | 10045 | 15750 |  |
| A | 214 | NA | NA | What is the only nut tree native to North America? | Pecan | .28 | .00 | .12 | .60 | .00 | 14881 |  | 6869 | 15229 |  |
| B | 217.5 | NA | NA | What do you call the purification or purgation of the emotions that is supposed to happen through art? | Catharsis | .28 | .00 | .67 | .06 | .00 | 21550 |  | 11313 | 12703 |  |
| B | 217.5 | NA | NA | What is the name of the island on which Napoleon was born? | Corsica | .28 | .11 | .37 | .22 | .02 | 17157 | 11753 | 6326 | 9247 | 2603 |
| B | 217.5 | NA | NA | What is the last name of the man who said, "I only regret that I have but one life to lose for my country"? | Hale | .28 | .20 | .15 | .37 | .00 | 23298 | 32231 | 7836 | 15899 |  |
| B | 217.5 | NA | NA | What is Jane Goodall famous for studying? | Chimpanzees | .28 | .00 | .09 | .61 | .02 | 13766 |  | 5993 | 6950 | 1423 |
| D | 220.5 | 161 | 231 | What is the city in which the baseball hall of fame is located? | Cooperstown | .27 | .11 | .30 | .32 | .00 | 15846 | 29957 | 16395 | 17366 |  |
| D | 220.5 | 215 | 184 | What is the last name of the movie actor who portrayed Spartacus? | Douglas | .27 | .14 | .30 | .27 | .02 | 17987 | 26993 | 8154 | 17235 | 15567 |
| A | 222 | NA | NA | Which famous explorer first introduced iced desserts into Europe? | Marco Polo | .26 | .02 | .61 | .11 | .00 | 15323 | 22975 | 11958 | 12593 |  |
| B | 223 | NA | NA | What is the name of the Mediterranean island south of Turkey that is an independent republic and a member of the Commonwealth? | Cyprus | .26 | .06 | .52 | .17 | .00 | 17103 | 24961 | 16212 | 21996 |  |
|  |  |  |  |  |  | CR (proportion of responses) | | | | | CR (response times) | | | | |
| Set | CR Rank | T. et al. rank | N & N rank | General Knowledge Question | Correct Answer | Correct | DR | DK | CE | OE | Correct | DR | DK | CE | OE |
| D | 224.5 | 165 | 164 | In addition to the Kentucky Derby and the Belmont Stakes what horse race comprises the Triple Crown? | Preakness | .26 | .33 | .26 | .15 | .00 | 17419 | 33554 | 13336 | 38009 |  |
| D | 224.5 | 185 | 190 | What is the last name of the author who wrote the James Bond novels? | Fleming | .26 | .24 | .44 | .06 | .00 | 15281 | 18820 | 10692 | 10623 |  |
| C | 226 | NA | NA | What open-air public theater was home to William Shakespeare's theatrical company? | Globe | .25 | .28 | .34 | .10 | .01 | 13837 | 21823 | 11494 | 17332 | 16600 |
| A | 228 | NA | NA | What is a Japanese dance drama featuring stylized narrative choreographic movements? | Kabuki | .25 | .05 | .54 | .16 | .00 | 16458 | 38472 | 8958 | 9470 |  |
| A | 228 | NA | NA | Auguste Rodin's "The Thinker" is found in the garden of a museum in which city? | Paris | .25 | .07 | .35 | .33 | .00 | 15409 | 12466 | 6763 | 17381 |  |
| A | 228 | NA | NA | What is Tiger Woods' real first name? | Eldrick | .25 | .16 | .44 | .16 | .00 | 20950 | 11943 | 5779 | 7966 |  |
| D | 230.5 | 197 | 216 | What is the last name of the playwright who wrote "A Streetcar Named Desire"? | Williams | .24 | .36 | .26 | .14 | .00 | 10647 | 17301 | 11345 | 20307 |  |
| D | 230.5 | 258 | 179 | What is the last name of the judge who was known as "The Law West of the Pecos?" | Bean | .24 | .12 | .64 | .00 | .00 | 16086 | 15225 | 10501 |  |  |
| B | 234 | NA | NA | What is the word meaning the promises from God to humans? | Covenant | .24 | .07 | .57 | .09 | .02 | 20923 | 39672 | 14397 | 17627 | 1436 |
| B | 234 | NA | NA | What is the last name of the cosmonaut who was the first person to orbit the earth? | Gagarin | .24 | .15 | .41 | .19 | .02 | 21094 | 13735 | 6844 | 15092 | 204 |
| B | 234 | NA | NA | What word denotes the series of units of weight used to measure gold, in which a pound equals twelve ounces? | Troy | .24 | .07 | .24 | .44 | .00 | 29836 | 34807 | 21159 | 24430 |  |
|  |  |  |  |  |  |  |  |  |  |  |  |  |  |  |  |
|  |  |  |  |  |  | CR (proportion of responses) | | | | | CR (response times) | | | | |
| Set | CR Rank | T. et al. rank | N & N rank | General Knowledge Question | Correct Answer | Correct | DR | DK | CE | OE | Correct | DR | DK | CE | OE |
| B | 234 | NA | NA | What do you call a formal exercise by a team of marchers? | Drill | .20 | .07 | .48 | .24 | .00 | 9686 | 11489 | 11632 | 16497 |  |
| B | 234 | NA | NA | What do you call stage entertainment consisting of various unrelated acts, such as magicians, acrobats, etc? | Vaudeville | .24 | .07 | .13 | .56 | .00 | 18930 | 18884 | 8936 | 14537 |  |
| C | 237.5 | NA | NA | What was the last name of the man who asked you not to squeeze the Charmin in the toilet paper commercial? | Whipple | .24 | .27 | .30 | .19 | .00 | 13727 | 17720 | 11837 | 16304 |  |
| C | 237.5 | NA | NA | What was built to confine the Minotaur and now refers to a maze? | Labyrinth | .24 | .09 | .57 | .07 | .03 | 17681 | 24425 | 16652 | 17404 | 14747 |
| A | 239.5 | NA | NA | What was Bugsy Siegel's real first name? | Benjamin | .23 | .12 | .53 | .12 | .00 | 15443 | 13702 | 7205 | 9675 |  |
| A | 239.5 | NA | NA | Which first lady was the first to live in the White House? | Abigail Adams | .23 | .11 | .40 | .26 | .00 | 24812 | 13319 | 7548 | 13851 |  |
| C | 241 | NA | NA | Who was the mythical Roman goddess of love? | Venus | .22 | .15 | .07 | .55 | .00 | 10481 | 20048 | 12811 | 12533 |  |
| B | 243.5 | NA | NA | What word means to voluntarily sacrifice (e.g., cargo) in order to lighten a ship or aircraft's load in time of distress? | Jettison | .22 | .28 | .39 | .09 | .02 | 25084 | 21433 | 15495 | 28439 | 1047 |
| B | 243.5 | NA | NA | What is the last name of the actor who played the scarecrow in the movie, "The Wizard of Oz"? | Bolger | .22 | .30 | .41 | .07 | .00 | 18611 | 13955 | 6108 | 12287 |  |
| B | 243.5 | NA | NA | What do you call the position of the arms when the hands are on the hips and the elbows are out? | Akimbo | .22 | .06 | .59 | .11 | .02 | 12283 | 18975 | 14391 | 20245 | 1587 |
|  |  |  |  |  |  |  |  |  |  |  |  |  |  |  |  |
|  |  |  |  |  |  | CR (proportion of responses) | | | | | CR (response times) | | | | |
| Set | CR Rank | T. et al. rank | N & N rank | General Knowledge Question | Correct Answer | Correct | DR | DK | CE | OE | Correct | DR | DK | CE | OE |
| B | 243.5 | NA | NA | What is the name of the substance derived from a whale that is used to make perfume? | Ambergris | .22 | .11 | .31 | .35 | .00 | 13285 | 16163 | 14049 | 19117 |  |
| D | 247 | 178 | 236 | What is the longest river In Asia? | Yangtze | .21 | .08 | .33 | .38 | .00 | 18020 | 26663 | 11236 | 18084 |  |
| D | 247 | 244 | 220 | What was the last name of the female star of the movie "Casablanca"? | Bergman | .21 | .21 | .26 | .32 | .00 | 16489 | 30673 | 11285 | 18596 |  |
| D | 247 | 255 | 163 | What was the last name of the Captain of the British ship "Bounty" when the mutiny occurred? | Bligh | .21 | .26 | .33 | .18 | .02 | 13568 | 17758 | 11469 | 20684 | 34556 |
| A | 249.5 | NA | NA | Halloween traces its origins to which pagan festival? | Samhain | .21 | .21 | .26 | .32 | .00 | 30885 | 18574 | 12555 | 14578 |  |
| A | 249.5 | NA | NA | Who wrote "The Rime of the Ancient Mariner"? | Samuel Taylor Coleridge | .21 | .11 | .51 | .18 | .00 | 21028 | 8416 | 6387 | 15276 |  |
| C | 251 | NA | NA | What was Fonzie's first name in television's 'Happy Days'? | Arthur | .21 | .36 | .19 | .22 | .01 | 9824 | 12425 | 8518 | 19532 | 17451 |
| B | 252 | NA | NA | What do you call a chain of rocks or coral at or near the surface of the water in an ocean? | Reef | .65 | .04 | .17 | .15 | .00 | 13373 | 24010 | 11476 | 16079 |  |
| D | 253.5 | 257 | 173 | What is the name of Germany's largest battleship that was sunk in World War II? | Bismarck | .20 | .15 | .58 | .08 | .00 | 17546 | 11639 | 10234 | 16802 |  |
| D | 253.5 | 269 | 239 | What is the last name of the first man to run the mile in under four minutes? | Bannister | .20 | .29 | .39 | .12 | .00 | 13070 | 13629 | 8544 | 10571 |  |
| A | 256.5 | NA | NA | What album holds the world record for copies sold? | Thriller | .19 | .05 | .30 | .46 | .00 | 25932 | 11216 | 8174 | 19628 |  |
|  |  |  |  |  |  |  |  |  |  |  |  |  |  |  |  |
|  |  |  |  |  |  | CR (proportion of responses) | | | | | CR (response times) | | | | |
| Set | CR Rank | T. et al. rank | N & N rank | General Knowledge Question | Correct Answer | Correct | DR | DK | CE | OE | Correct | DR | DK | CE | OE |
| A | 256.5 | NA | NA | What food is often used as a substitute for ice cream in advertising photo shoots? | Mashed Potato | .19 | .00 | .44 | .37 | .00 | 29158 |  | 10944 | 19367 |  |
| A | 256.5 | NA | NA | What was Sinatra's chart-topping duet with his daughter Nancy? | Somethin' Stupid | .19 | .37 | .32 | .11 | .02 | 35848 | 13217 | 11467 | 11553 | 14281 |
| A | 256.5 | NA | NA | What philosopher was considered the ugliest man in Athens? | Socrates | .19 | .04 | .61 | .16 | .00 | 20594 | 7249 | 6896 | 8814 |  |
| B | 259 | NA | NA | What word means relating to or situated on the back, especially of an animal? | Dorsal | .19 | .06 | .31 | .44 | .00 | 17108 | 25175 | 17826 | 19952 |  |
| D | 260 | 270 | 240 | What is the last name of the author who wrote "The Brothers Karamazov"? | Dostoyevsky | .18 | .29 | .35 | .17 | .02 | 20312 | 17265 | 10628 | 22741 | 18257.00 |
| C | 261 | NA | NA | What is the name of the obnoxious rooster in Warner Brothers cartoons? | Foghorn Leghorn | .18 | .27 | .39 | .15 | .01 | 15442 | 16981 | 13138 | 20264 | 7556 |
| A | 262.5 | NA | NA | Who is the lead singer of Limp Bizkit? | Fred Durst | .18 | .04 | .75 | .04 | .00 | 17644 | 5162 | 4291 | 10280 |  |
| A | 262.5 | NA | NA | In 1984 Pres. Ronald Reagan won every state except one. Which one did he lose? | Minnesota | .18 | .14 | .32 | .37 | .00 | 34411 | 13749 | 29632 | 14984 |  |
| B | 266.5 | NA | NA | What is the name of the man who removed the thorn from the lion's paw in the story from Aesop's fables? | Androcles | .17 | .43 | .37 | .04 | .00 | 31326 | 12655 | 11297 | 8455 |  |
| B | 266.5 | NA | NA | What is the last name of 1980 Olympic hopeful Randy Gardener's ice skating partner? | Babilonia | .17 | .19 | .59 | .06 | .00 | 16390 | 7661 | 9571 | 7556 |  |
| B | 266.5 | NA | NA | What is the name of the formerly independent country north of Lithuania and Latvia that was absorbed into USSR? | Estonia | .17 | .11 | .61 | .11 | .00 | 13973 | 16063 | 14160 | 23478 |  |
|  |  |  |  |  |  | CR (proportion of responses) | | | | | CR (response times) | | | | |
| Set | CR Rank | T. et al. rank | N & N rank | General Knowledge Question | Correct Answer | Correct | DR | DK | CE | OE | Correct | DR | DK | CE | OE |
| B | 266.5 | NA | NA | Who is the messenger of the gods in Greek mythology? | Hermes | .17 | .15 | .30 | .37 | .02 | 20573 | 9507 | 5386 | 9099 | 1224 |
| D | 266.5 | 170 | 228 | What is the name of the Chinese religion founded by Lao Tse? | Taoism | .17 | .15 | .27 | .41 | .00 | 16549 | 20342 | 13993 | 23661 |  |
| D | 266.5 | 195 | 171 | What was the name of Alexander Graham Bell's assistant? | Watson | .17 | .30 | .42 | .11 | .00 | 8786 | 12086 | 9479 | 18726 |  |
| A | 272 | NA | NA | What is a poem in which the first letters of each line spell a word? | An acrostic | .16 | .07 | .63 | .14 | .00 | 22330 | 9354 | 9710 | 16068 |  |
| A | 272 | NA | NA | What story is the first to feature Edgar Allan Poe's amateur detective C. Auguste Dupin? | Murders in the Rue Morgue | .16 | .09 | .67 | .09 | .00 | 31622 | 13857 | 7980 | 10150 |  |
| A | 272 | NA | NA | Niels Bohr used quantum mechanics to describe which element? | Hydrogen | .16 | .04 | .60 | .21 | .00 | 16408 | 8823 | 7726 | 16595 |  |
| A | 272 | NA | NA | What is the alternative name for the Sea of Cortez? | Gulf of California | .16 | .05 | .61 | .18 | .00 | 33543 | 8571 | 5307 | 14776 |  |
| A | 272 | NA | NA | Ailurophobia is the fear of what? | Cats | .16 | .11 | .54 | .19 | .00 | 23270 | 16096 | 10261 | 14244 |  |
| D | 275.5 | 184 | 261 | What is the last name of the composer who wrote the opera "Don Giovanni?" | Mozart | .15 | .15 | .47 | .23 | .00 | 12890 | 11289 | 9230 | 11911 |  |
| D | 275.5 | 274 | 253 | What is the last name of the author of "Our Town"? | Wilder | .15 | .39 | .29 | .15 | .02 | 8889 | 12942 | 9899 | 14399 | 7210.00 |
| B | 277.5 | NA | NA | What word means to observe and offer unwanted advice or comment, especially at a card game? | Kibitz | .15 | .11 | .63 | .11 | .00 | 18413 | 14267 | 16651 | 26847 |  |
| B | 277.5 | NA | NA | What is the last name of the author of Jonathan Livingston Seagull? | Bach | .15 | .33 | .37 | .13 | .02 | 32444 | 10649 | 7267 | 16826 | 1058 |
| A | 280.5 | NA | NA | Who invented flexible photographic film? | George Eastman | .14 | .02 | .47 | .37 | .00 | 18771 | 10976 | 6508 | 10567 |  |
| A | 280.5 | NA | NA | Who said "You'd be surprised how much it costs to look this cheap?" | Dolly Parton | .14 | .02 | .60 | .23 | .02 | 16053 | 9550 | 6320 | 26671 | 6635 |
|  |  |  |  |  |  | CR (proportion of responses) | | | | | CR (response times) | | | | |
| Set | CR Rank | T. et al. rank | N & N rank | General Knowledge Question | Correct Answer | Correct | DR | DK | CE | OE | Correct | DR | DK | CE | OE |
| A | 280.5 | NA | NA | Which Egyptian god was often represented as a falcon? | Horus | .14 | .07 | .56 | .23 | .00 | 20733 | 9235 | 7019 | 9759 |  |
| A | 280.5 | NA | NA | What is U2 singer Bono's real name? | Paul Hewson | .14 | .14 | .60 | .12 | .00 | 28549 | 10073 | 4676 | 13602 |  |
| D | 283.5 | 174 | 165 | In which city does the Cotton Bowl take place? | Dallas | .14 | .12 | .29 | .45 | .00 | 8941 | 18173 | 11757 | 19731 |  |
| D | 283.5 | 207 | 284 | What is the capital of Australia? | Canberra | .14 | .11 | .08 | .68 | .00 | 10217 | 29768 | 6353 | 12974 |  |
| B | 286 | NA | NA | What do you call a secret agreement or pact (as between lovers)? | Tryst | .13 | .17 | .50 | .20 | .00 | 11224 | 28895 | 20193 | 20711 |  |
| B | 286 | NA | NA | What was the name of the capital of the Aztec empire, which was located where Mexico City is today? | Tenochtitlan | .13 | .30 | .52 | .06 | .00 | 19826 | 12445 | 16208 | 16009 |  |
| B | 286 | NA | NA | What do you call a law that gives exclusive right of inheritance to the eldest son? | Primogeniture | .13 | .09 | .63 | .13 | .02 | 32369 | 100482 | 12464 | 28574 | 1422 |
| A | 288 | NA | NA | Which lawyer and presidential adviser in 1969 became the only Supreme Court justice to resign under threat of impeachment? | Abe Fortas | .12 | .16 | .56 | .16 | .00 | 58768 | 16354 | 13337 | 18721 |  |
| D | 289 | 236 | 250 | Who was the first ruler of the Holy Roman Empire? | Charlemagne | .12 | .18 | .20 | .50 | .00 | 15337 | 18988 | 11463 | 16869 |  |
| B | 290 | NA | NA | What is the national park in Utah, northeast of Zion, which is famous for the rock formations in its canyon? | Bryce | .11 | .11 | .48 | .30 | .00 | 11623 | 20662 | 12561 | 34242 |  |
| D | 292 | 166 | 204 | What is the last name of the criminal who was killed by FBI agents outside of a Chicago movie theater? | Dillinger | .11 | .26 | .23 | .41 | .00 | 16426 | 25934 | 16669 | 21629 |  |
| D | 292 | 273 | 252 | What is the name of the instrument used to measure wind speed? | Anemometer | .11 | .35 | .29 | .26 | .00 | 13160 | 24394 | 16035 | 19183 |  |
|  |  |  |  |  |  |  |  |  |  |  |  |  |  |  |  |
|  |  |  |  |  |  | CR (proportion of responses) | | | | | CR (response times) | | | | |
| Set | CR Rank | T. et al. rank | N & N rank | General Knowledge Question | Correct Answer | Correct | DR | DK | CE | OE | Correct | DR | DK | CE | OE |
| D | 292 | 275 | 256 | What is the last name of the singer who made a hit recording of the song "Who's Sorry Now?" | Francis | .11 | .24 | .32 | .33 | .00 | 18159 | 25518 | 16721 | 20427 |  |
| A | 299 | NA | NA | Who designed Central Park in New York City? | Frederick Law Olmstead | .11 | .14 | .61 | .14 | .00 | 36316 | 7522 | 7969 | 15723 |  |
| A | 299 | NA | NA | The Mayan and Aztec peoples used cocoa beans not only to make a delicious beverage but also as...? | Currency | .11 | .02 | .42 | .46 | .00 | 16839 | 14289 | 12274 | 16392 |  |
| A | 299 | NA | NA | Which Bronte sister died of tuberculosis in 1848 and was famously buried in a coffin that was a meager 16 inches (41 cm) wide? | Emily | .11 | .02 | .67 | .21 | .00 | 29157 | 4482 | 9981 | 18630 |  |
| A | 299 | NA | NA | What breed of dog is known for making a yodeling noise instead of barking? | Basenji | .11 | .04 | .60 | .26 | .00 | 21285 | 6231 | 6610 | 12449 |  |
| A | 299 | NA | NA | What was author George Eliot's real name? | Mary Ann Evans | .11 | .16 | .63 | .11 | .00 | 23669 | 9986 | 5990 | 18985 |  |
| A | 299 | NA | NA | In what geological period did birds evolve? | Jurassic | .11 | .04 | .70 | .16 | .00 | 15563 | 12689 | 7598 | 11431 |  |
| A | 299 | NA | NA | Polo consists of 8 periods called what? | Chukkers | .11 | .07 | .67 | .16 | .00 | 21285 | 9136 | 5721 | 14133 |  |
| A | 299 | NA | NA | With which band was David Johansen the lead singer? | The New York Dolls | .11 | .12 | .70 | .07 | .00 | 23285 | 11808 | 6719 | 10027 |  |
| A | 299 | NA | NA | What does a philomath have a love for? | Learning | .11 | .02 | .44 | .44 | .00 | 40747 | 6116 | 8350 | 11217 |  |
| A | 299 | NA | NA | What was the title of Dr. Seuss' first book? | And to Think That I Saw It on Mulberry Street | .11 | .07 | .26 | .54 | .02 | 39341 | 7315 | 7440 | 12195 | 4478 |
|  |  |  |  |  |  | CR (proportion of responses) | | | | | CR (response times) | | | | |
| Set | CR Rank | T. et al. rank | N & N rank | General Knowledge Question | Correct Answer | Correct | DR | DK | CE | OE | Correct | DR | DK | CE | OE |
| A | 299 | NA | NA | What was the name of George of the Jungle's pet elephant? | Shep | .11 | .16 | .60 | .14 | .00 | 33914 | 9662 | 7684 | 8908 |  |
| B | 306 | NA | NA | What word means the form of something in the form of a spiral or coil? | Helix | .09 | .13 | .46 | .31 | .00 | 14271 | 27586 | 17240 | 21407 |  |
| B | 306 | NA | NA | What do you call a verb that does not take a direct object? | Intransitive | .09 | .15 | .37 | .37 | .02 | 12782 | 18412 | 8718 | 14873 | 1535 |
| B | 306 | NA | NA | What is the last name of the candidate who ran as an independent against Reagan and Carter in 1980? | Anderson | .09 | .33 | .20 | .37 | .00 | 24007 | 19857 | 15882 | 14512 |  |
| D | 308.5 | 242 | 232 | What is the name of the brightest star in the sky excluding the sun? | Sirius | .09 | .17 | .21 | .53 | .00 | 11556 | 22064 | 17745 | 16119 |  |
| D | 308.5 | 261 | 197 | What is the last name of the doctor who performed the first successful human heart transplant? | Barnard | .09 | .56 | .21 | .14 | .00 | 13421 | 16996 | 12359 | 13273 |  |
| C | 310 | NA | NA | What mythical creature did Perseus kill? | Medusa | .09 | .10 | .45 | .33 | .03 | 21333 | 11692 | 9114 | 15287 | 19954 |
| A | 313 | NA | NA | Who was the greatest poet of Russia's golden age? | Alexander Pushkin | .09 | .02 | .75 | .14 | .00 | 20525 | 11523 | 5359 | 16082 |  |
| A | 313 | NA | NA | Who wrote the "Threepenny Opera"? | Bertolt Brecht | .09 | .11 | .65 | .16 | .00 | 12969 | 10299 | 5859 | 9852 |  |
| A | 313 | NA | NA | What was the first product to have a barcode? | Wrigley gum | .09 | .07 | .70 | .14 | .00 | 12826 | 8190 | 6569 | 19163 |  |
| A | 313 | NA | NA | In which Cleveland suburb did actor Paul Newman grow up? | Shaker Heights | .09 | .04 | .77 | .11 | .00 | 15439 | 4394 | 6829 | 9603 |  |
| A | 313 | NA | NA | The term "op-ed" is a shortened form of which phrase? | Opposite the editorial page | .09 | .05 | .14 | .72 | .00 | 25210 | 20903 | 24251 | 20171 |  |
| D | 316.5 | 286 | 277 | What was the last name of Billy the Kid? | Bonney | .08 | .18 | .62 | .12 | .00 | 10035 | 13799 | 8053 | 31135 |  |
|  |  |  |  |  |  |  |  |  |  |  |  |  |  |  |  |
|  |  |  |  |  |  | CR (proportion of responses) | | | | | CR (response times) | | | | |
| Set | CR Rank | T. et al. rank | N & N rank | General Knowledge Question | Correct Answer | Correct | DR | DK | CE | OE | Correct | DR | DK | CE | OE |
| D | 316.5 | 288 | 279 | From what musical is the song “Baubles Bangles And Beads”? | Kismet | .08 | .23 | .56 | .14 | .00 | 15043 | 18352 | 9284 | 30152 |  |
| B | 318 | NA | NA | What word means lasting only a very brief time? | Ephemeral | .07 | .11 | .17 | .65 | .00 | 10347 | 27578 | 13110 | 19020 |  |
| A | 324 | NA | NA | What is the name of Ulysses Everett McGill's hair pomade of choice in "O Brother Where Art Thou"? | Dapper Dan | .07 | .16 | .68 | .09 | .00 | 20230 | 13041 | 10119 | 11102 |  |
| A | 324 | NA | NA | What philosopher was imprisoned by the British government in 1918 for campaigning against war and conscription? | Bertrand Russell | .07 | .04 | .79 | .11 | .00 | 12712 | 6899 | 8768 | 16539 |  |
| A | 324 | NA | NA | The theme tune for 'Monty Python's Flying Circus' was written by which composer? | John Philip Sousa | .07 | .04 | .74 | .16 | .00 | 11039 | 15978 | 5406 | 11159 |  |
| A | 324 | NA | NA | What is a chord consisting of three tones of a diatonic scale called? | Triad | .07 | .11 | .72 | .11 | .00 | 8144 | 10207 | 8345 | 9650 |  |
| A | 324 | NA | NA | What was the name of the U.S. surveillance ship that was attacked by North Vietnam in the Gulf of Tonkin? | USS Maddox | .07 | .35 | .44 | .14 | .00 | 29591 | 11837 | 12397 | 17125 |  |
| A | 324 | NA | NA | What do A. E. Housman's initials stand for? | Alfred Edward | .07 | .05 | .77 | .11 | .00 | 9415 | 12839 | 7349 | 11795 |  |
| A | 324 | NA | NA | In which film did Doris Day sing the Oscar winning song "Secret Love"? | Calamity Jane | .07 | .14 | .65 | .14 | .00 | 31604 | 11193 | 7489 | 26195 |  |
| A | 324 | NA | NA | Which first lady initiated the Easter egg roll on the White House lawn? | Lucy Hayes | .07 | .07 | .33 | .53 | .00 | 21233 | 22281 | 11368 | 19008 |  |
| A | 324 | NA | NA | Who is the author of "Ozymandias"? | Percy Bysshe Shelley | .07 | .07 | .75 | .11 | .00 | 15205 | 11468 | 6705 | 13483 |  |
|  |  |  |  |  |  | CR (proportion of responses) | | | | | CR (response times) | | | | |
| Set | CR Rank | T. et al. rank | N & N rank | General Knowledge Question | Correct Answer | Correct | DR | DK | CE | OE | Correct | DR | DK | CE | OE |
| A | 324 | NA | NA | What is LL Cool J.'s real name? | James Todd Smith | .07 | .09 | .70 | .14 | .00 | 25102 | 7285 | 4660 | 9084 |  |
| A | 324 | NA | NA | Aldebaran is the brightest star in which constellation? | Taurus | .07 | .02 | .68 | .23 | .00 | 43832 | 4806 | 6541 | 9553 |  |
| B | 330 | NA | NA | What do you call a solemn declaration made by a person who conscientiously declines taking an oath? | Affirmation | .06 | .07 | .46 | .41 | .00 | 33333 | 33898 | 21773 | 20778 |  |
| A | 331 | NA | NA | Who assassinated Mohandas Gandhi? | A Hindu nationalist | .05 | .18 | .68 | .09 | .00 | 80718 | 5162 | 6071 | 12428 |  |
| A | 338.5 | NA | NA | What were the first tennis racket strings made of? | Sheep gut | .05 | .00 | .26 | .68 | .00 | 65887 |  | 12367 | 9068 |  |
| A | 338.5 | NA | NA | Myosotis is the latin name for which type of flower? | Forget-Me-Not | .05 | .02 | .77 | .16 | .00 | 35891 | 10667 | 13684 | 11348 |  |
| A | 338.5 | NA | NA | What is the world governing body of tennis? | International Tennis Federation | .05 | .07 | .63 | .25 | .00 | 20699 | 10550 | 9707 | 13956 |  |
| A | 338.5 | NA | NA | Who was the lead vocalist for 'The Dakotas' during the 1960s?" | Billy J. Kramer | .05 | .02 | .86 | .07 | .00 | 22038 | 8985 | 7820 | 14865 |  |
| A | 338.5 | NA | NA | Where were the 1952 Olympics held? | Helsinki | .05 | .14 | .53 | .28 | .00 | 23330 | 12450 | 5344 | 16238 |  |
| A | 338.5 | NA | NA | The 1912 Summer Olympics were held in what city? | Stockholm | .05 | .00 | .58 | .37 | .00 | 23785 |  | 6357 | 13951 |  |
| A | 338.5 | NA | NA | W. Somerset Maugham's 1919 novel "The Moon and Sixpence" is based on the life of what artist? | Paul Gauguin | .05 | .00 | .81 | .14 | .00 | 10106 |  | 8766 | 14850 |  |
| A | 338.5 | NA | NA | In which city is the Encyclopedia Britannica published? | Chicago | .05 | .00 | .46 | .49 | .00 | 36673 |  | 8252 | 12085 |  |
| A | 338.5 | NA | NA | Who discovered the law of electrolysis? | Michael Faraday | .05 | .05 | .77 | .12 | .00 | 16759 | 9599 | 4773 | 13635 |  |
| A | 338.5 | NA | NA | Which French author's novel "Germinal" depicts life in a mining community? | Emile Zola | .05 | .02 | .82 | .11 | .00 | 11587 | 7068 | 8166 | 11823 |  |
|  |  |  |  |  |  | CR (proportion of responses) | | | | | CR (response times) | | | | |
| Set | CR Rank | T. et al. rank | N & N rank | General Knowledge Question | Correct Answer | Correct | DR | DK | CE | OE | Correct | DR | DK | CE | OE |
| A | 338.5 | NA | NA | What was the most played song on the radio in the United States during the 20th century? | You've Lost That Lovin' Feelin' | .05 | .04 | .51 | .40 | .00 | 63740 | 11777 | 11603 | 20277 |  |
| A | 338.5 | NA | NA | What is Australia's highest mountain? | Mount Kosciuszko | .05 | .04 | .81 | .11 | .00 | 33928 | 5988 | 4693 | 10465 |  |
| A | 338.5 | NA | NA | Tony Drago is a snooker professional from which country? | Malta | .05 | .00 | .63 | .32 | .00 | 18428 |  | 5170 | 13894 |  |
| A | 338.5 | NA | NA | Who was the first person to admit to practicing witchcraft in Salem? | Tituba | .05 | .09 | .74 | .12 | .00 | 16089 | 12010 | 6574 | 17484 |  |
| D | 346.5 | 208 | 259 | What is the last name of the artist who painted "The Persistence of Memory"? | Dali | .05 | .03 | .88 | .05 | .00 | 6984 | 9294 | 8272 | 23703 |  |
| D | 346.5 | 279 | 266 | What is the last name of the author of "The Agony and the Ecstasy"? | Stone | .05 | .39 | .47 | .09 | .00 | 14587 | 14959 | 8578 | 18728 |  |
| B | 348 | NA | NA | What word means to deposit (something valuable) as a security for money borrowed? | Pawn | .04 | .07 | .07 | .81 | .00 | 31271 | 23299 | 9451 | 20206 |  |
| A | 359 | NA | NA | Jockey Lester Piggott served 3 years in prison for what crime? | Tax evasion | .04 | .02 | .67 | .28 | .00 | 35531 | 10254 | 8784 | 15413 |  |
| A | 359 | NA | NA | What is the capital of Guinea-Bissau? | Bissau | .04 | .00 | .88 | .09 | .00 | 26390 |  | 5508 | 10835 |  |
| A | 359 | NA | NA | Who patented a burglar proof lock described as "magic and infallible"? | Linus Yale | .04 | .00 | .77 | .19 | .00 | 6951 |  | 10869 | 18322 |  |
| A | 359 | NA | NA | What was the first graphical browser for the World Wide Web? | Mosaic | .04 | .19 | .40 | .37 | .00 | 14467 | 13427 | 18345 | 18816 |  |
| A | 359 | NA | NA | What is Spike Milligan's real first name? | Terence | .04 | .00 | .79 | .18 | .00 | 24581 |  | 5104 | 7134 |  |
| A | 359 | NA | NA | Who was the famous actor who played the Swedish Chef's uncle in an episode of "The Muppet Show"? | Danny Kaye | .04 | .07 | .82 | .07 | .00 | 17850 | 11195 | 8541 | 11022 |  |
|  |  |  |  |  |  | CR (proportion of responses) | | | | | CR (response times) | | | | |
| Set | CR Rank | T. et al. rank | N & N rank | General Knowledge Question | Correct Answer | Correct | DR | DK | CE | OE | Correct | DR | DK | CE | OE |
| A | 359 | NA | NA | Who became Archbishop of Canterbury in 1980? | Robert Runcie | .04 | .04 | .84 | .09 | .00 | 40382 | 8301 | 6372 | 10824 |  |
| A | 359 | NA | NA | What opera by Giacomo Puccini was left unfinished at his death? | Turandot | .04 | .05 | .82 | .09 | .00 | 21760 | 15518 | 5792 | 10938 |  |
| A | 359 | NA | NA | Which Cleveland mayor was the first African American mayor of a major U.S. city? | Carl Stokes | .04 | .16 | .72 | .09 | .00 | 16633 | 8581 | 7951 | 10417 |  |
| A | 359 | NA | NA | What is the largest known butterfly? | Queen Alexandra's Birdwing | .04 | .02 | .56 | .39 | .00 | 28945 | 10710 | 6795 | 11331 |  |
| A | 359 | NA | NA | Who ruled Jerusalem from 1099 to 1187 CE? | European Crusaders | .04 | .05 | .53 | .39 | .00 | 27699 | 16859 | 10032 | 16245 |  |
| A | 359 | NA | NA | Name the title character of a popular rap-themed video game that was originally released on the Sony PlayStation in 1996. | PaRappa the Rapper | .04 | .00 | .84 | .12 | .00 | 11024 |  | 11773 | 13394 |  |
| A | 359 | NA | NA | What was the first rap song to hit number one on the Billboard Hot 100 chart? | Ice Ice Baby | .04 | .02 | .81 | .14 | .00 | 31573 | 14267 | 7820 | 22162 |  |
| A | 359 | NA | NA | Which British city was named Deva by the Romans? | Chester | .04 | .00 | .67 | .30 | .00 | 30408 |  | 6713 | 9990 |  |
| A | 359 | NA | NA | Who wrote the 1859 novel "Oblomov" whose lazy and daydreaming titular character satirizes the contemporary Russian nobility? | Ivan Goncharov | .04 | .04 | .77 | .16 | .00 | 15504 | 8821 | 7708 | 14721 |  |
| A | 359 | NA | NA | The ice-cream cone originates from what American state?) | Missouri | .04 | .02 | .42 | .53 | .00 | 9370 | 6016 | 6849 | 15466 |  |
| A | 359 | NA | NA | What does a brandophile collect? | Cigar bands | .04 | .00 | .67 | .30 | .00 | 20645 |  | 7719 | 14666 |  |
|  |  |  |  |  |  |  |  |  |  |  |  |  |  |  |  |
|  |  |  |  |  |  |  |  |  |  |  |  |  |  |  |  |
|  |  |  |  |  |  | CR (proportion of responses) | | | | | CR (response times) | | | | |
| Set | CR Rank | T. et al. rank | N & N rank | General Knowledge Question | Correct Answer | Correct | DR | DK | CE | OE | Correct | DR | DK | CE | OE |
| A | 359 | NA | NA | Which baseball player holds the record for most professional hits across all baseball leagues? | Ichiro Suzuki | .04 | .07 | .39 | .51 | .00 | 17143 | 24274 | 11393 | 16441 |  |
| A | 359 | NA | NA | Who received the 1951 Nobel prize in physics? | Ernest Walton | .04 | .02 | .68 | .25 | .02 | 27616 | 3779 | 7475 | 11163 | 2300 |
| A | 359 | NA | NA | What is the name for Earth on the Little Prince's planet? | Asteroid MU-330 | .04 | .02 | .84 | .09 | .02 | 23985 | 10111 | 6521 | 16990 | 484 |
| A | 359 | NA | NA | What is the name given to the short tail of a rabbit? | Scut | .04 | .05 | .44 | .47 | .00 | 33676 | 22513 | 11839 | 14848 |  |
| D | 370.5 | 282 | 270 | What is the last name of the discoverer of the vaccination for smallpox? | Jenner | .03 | .38 | .30 | .29 | .00 | 15349 | 17347 | 15901 | 17995 |  |
| D | 370.5 | 292 | 287 | What is the last name of the poet who wrote the line "Into each life a little rain must fall"? | Longfellow | .03 | .17 | .68 | .11 | .02 | 19172 | 12322 | 10961 | 15474 | 6360.00 |
| B | 372 | NA | NA | What do you call the weapon used by Indians and gauchos of South America to entangle the legs of cattle and other animals? | Bola | .02 | .09 | .31 | .57 | .00 | 14187 | 17274 | 10183 | 11157 |  |
| A | 384 | NA | NA | What school did Billy Bunter attend? | Greyfriars | .02 | .00 | .86 | .12 | .00 | 36425 |  | 5627 | 8578 |  |
| A | 384 | NA | NA | Who is the patron saint of Armenia? | St. Gregory | .02 | .02 | .86 | .11 | .00 | 23513 | 5000 | 6777 | 9907 |  |
| A | 384 | NA | NA | What is the name for a curry made of spinach and cheese? | Palak Paneer | .02 | .05 | .79 | .14 | .00 | 6519 | 9198 | 8748 | 13263 |  |
| A | 384 | NA | NA | Who was Australia's first prime minister? | Edmund Barton | .02 | .00 | .88 | .11 | .00 | 20938 |  | 4767 | 7679 |  |
| A | 384 | NA | NA | What is caterpillar waste called? | Frass | .02 | .04 | .67 | .28 | .00 | 17023 | 8902 | 6455 | 9221 |  |
| A | 384 | NA | NA | What is the basic unit of currency for Myanmar? | Kyat | .02 | .02 | .82 | .14 | .00 | 23922 | 19752 | 7474 | 8504 |  |
| A | 384 | NA | NA | What Latin American author wrote in green ink? | Pablo Neruda | .02 | .04 | .81 | .14 | .00 | 63131 | 6615 | 6770 | 12664 |  |
|  |  |  |  |  |  |  |  |  |  |  |  |  |  |  |  |
|  |  |  |  |  |  | CR (proportion of responses) | | | | | CR (response times) | | | | |
| Set | CR Rank | T. et al. rank | N & N rank | General Knowledge Question | Correct Answer | Correct | DR | DK | CE | OE | Correct | DR | DK | CE | OE |
| A | 384 | NA | NA | Who is the Hindu god associated with rain? | Indra | .02 | .02 | .81 | .16 | .00 | 21960 | 3316 | 5507 | 8357 |  |
| A | 384 | NA | NA | Gwendolyn Brooks won a Pulitzer in 1950 for which book of poems? | Annie Allen | .02 | .04 | .88 | .07 | .00 | 36469 | 6919 | 14028 | 11925 |  |
| A | 384 | NA | NA | Who invented the aerosol spray can? | Erik Rotheim | .02 | .04 | .88 | .07 | .00 | 30853 | 18156 | 7536 | 11005 |  |
| A | 384 | NA | NA | Who is the only one of Milton's contemporaries to be mentioned by name in"Paradise Lost"? | Galileo | .02 | .09 | .75 | .14 | .00 | 24965 | 13004 | 8020 | 14513 |  |
| A | 384 | NA | NA | What type of Cubism incorporated pieces of newspaper? | Synthetic Cubism | .02 | .04 | .77 | .18 | .00 | 41332 | 12809 | 7997 | 19329 |  |
| A | 384 | NA | NA | Who sang "Amityville House On The Hill"? | Lovebug Starski | .02 | .00 | .91 | .07 | .00 | 26174 |  | 4826 | 9930 |  |
| A | 384 | NA | NA | Which German duo have sold over 85 million records? | Modern Talking | .02 | .04 | .79 | .16 | .00 | 30052 | 11138 | 12244 | 22857 |  |
| A | 384 | NA | NA | In which British national daily newspaper does Rupert The Bear appear? | The Daily Express | .02 | .00 | .75 | .23 | .00 | 25434 |  | 5703 | 15536 |  |
| A | 384 | NA | NA | What is the tallest mountain in Iran? | Mount Damavand | .02 | .02 | .88 | .09 | .00 | 17115 | 5116 | 4461 | 6989 |  |
| A | 384 | NA | NA | What was the name of William Wordsworth's sister? | Dorothy | .02 | .02 | .79 | .18 | .00 | 22380 | 9899 | 5376 | 7361 |  |
| A | 384 | NA | NA | Which team won the first World Series in 1903? | Boston Americans | .02 | .02 | .58 | .39 | .00 | 26686 | 7780 | 6462 | 12324 |  |
| A | 384 | NA | NA | What television talent show did Mary Hopkin win? | Opportunity Knocks | .02 | .00 | .84 | .14 | .00 | 58720 |  | 8310 | 25501 |  |
| A | 384 | NA | NA | What is the name of the woman Romeo is infatuated with before he meets Juliet? | Rosaline | .02 | .19 | .65 | .14 | .00 | 19058 | 10446 | 9822 | 14098 |  |
|  |  |  |  |  |  | CR (proportion of responses) | | | | | CR (response times) | | | | |
| Set | CR Rank | T. et al. rank | N & N rank | General Knowledge Question | Correct Answer | Correct | DR | DK | CE | OE | Correct | DR | DK | CE | OE |
| A | 384 | NA | NA | Which Suffolk town was the birthplace of Benjamin Britten? | Lowestoft | .02 | .00 | .86 | .12 | .00 | 29768 |  | 5166 | 28439 |  |
| A | 384 | NA | NA | What liqueur goes into making a 'Snowball' cocktail? | Advocaat | .02 | .00 | .70 | .28 | .00 | 26015 |  | 12779 | 14928 |  |
| A | 384 | NA | NA | Alan McGee signed Oasis to which label in 1993? | Creation | .02 | .02 | .79 | .18 | .00 | 35046 | 4812 | 6032 | 11608 |  |
| D | 398.5 | 203 | 286 | What is the last name of the twenty-first U.S. President? | Arthur | .02 | .27 | .36 | .35 | .00 | 45963 | 13687 | 11887 | 22267 |  |
| D | 398.5 | 220 | 296 | What is the last name of the man who supposedly killed Jesse James? | Ford | .02 | .30 | .39 | .29 | .00 | 5337 | 13886 | 10878 | 23342 |  |
| D | 398.5 | 229 | 297 | What is the last name of the First American author to win the Nobel Prize for Literature? | Lewis | .02 | .06 | .70 | .23 | .00 | 13019 | 20264 | 12598 | 30700 |  |
| D | 398.5 | 240 | 288 | What was the name of the nuclear submarine that sunk in the Atlantic in 1963? | Thresher | .02 | .35 | .52 | .11 | .02 | 9856 | 15156 | 11049 | 18355 | 7720 |
| D | 398.5 | 281 | 269 | What is the name of the first movie to receive the Academy Award for Best Picture? | Wings | .02 | .06 | .61 | .32 | .00 | 4146 | 9859 | 10352 | 24511 |  |
| D | 398.5 | 291 | 283 | What is the name of a number two wood in golf? | Brassie | .02 | .05 | .61 | .33 | .00 | 9641 | 25130 | 11792 | 14946 |  |
| A | 411.5 | NA | NA | What is the first-person plural pronoun in Hindi? | Hum | .00 | .00 | .88 | .12 | .00 |  |  | 8843 | 13014 |  |
| A | 411.5 | NA | NA | Who dubbed Australia "the lucky country"? | Donald Horne | .00 | .00 | .89 | .11 | .00 |  |  | 5487 | 9685 |  |
| A | 411.5 | NA | NA | Which Pulitzer Prize winner has won more than twice? | Robert Frost | .00 | .07 | .72 | .19 | .02 |  | 8027 | 10565 | 16286 | 5172 |
| A | 411.5 | NA | NA | What was the first newspaper to utilize advertisements for revenue and the "penny paper" format? | The Sun | .00 | .04 | .63 | .33 | .00 |  | 9795 | 10159 | 20479 |  |
|  |  |  |  |  |  | CR (proportion of responses) | | | | | CR (response times) | | | | |
| Set | CR Rank | T. et al. rank | N & N rank | General Knowledge Question | Correct Answer | Correct | DR | DK | CE | OE | Correct | DR | DK | CE | OE |
| A | 411.5 | NA | NA | What is the capital of Burundi? | Bujumbura | .00 | .04 | .84 | .12 | .00 |  | 7754 | 6243 | 13595 |  |
| A | 411.5 | NA | NA | Which country won the first women's Olympic handball gold medal in 1976? | The Soviet Union | .00 | .02 | .63 | .35 | .00 |  | 15476 | 5963 | 11039 |  |
| A | 411.5 | NA | NA | A sufferer from Boanthropy believes he is what? | An ox | .00 | .00 | .79 | .21 | .00 |  |  | 8956 | 11360 |  |
| A | 411.5 | NA | NA | Which animal has the widest hearing range? | Dolphin | .00 | .02 | .44 | .54 | .00 |  | 3259 | 9510 | 19285 |  |
| A | 411.5 | NA | NA | What is the name of a dried legume? | Pulse | .00 | .05 | .30 | .65 | .00 |  | 14634 | 9328 | 14940 |  |
| A | 411.5 | NA | NA | What was the name of Grotbags' pet in the TV show "Emu's World"? | Croc | .00 | .00 | .93 | .07 | .00 |  |  | 7506 | 9643 |  |
| A | 411.5 | NA | NA | Who replaced Betty Boothroyd as "Speaker of the House of Commons"? | Michael Martin | .00 | .00 | .91 | .09 | .00 |  |  | 6159 | 10743 |  |
| A | 411.5 | NA | NA | Which mountain range runs along the south shore of the Caspian Sea? | Elburz | .00 | .05 | .54 | .40 | .00 |  | 6911 | 7238 | 14466 |  |
| A | 411.5 | NA | NA | What is the meaning of the name Himalaya in Sanskrit? | Abode of snow | .00 | .00 | .75 | .25 | .00 |  |  | 5610 | 12512 |  |
| A | 411.5 | NA | NA | What was the first Arcade game ever released? | Computer Space | .00 | .02 | .23 | .75 | .00 |  | 4474 | 6649 | 10060 |  |
| A | 411.5 | NA | NA | Which branch of physics is particularly useful in designing bridges? | Statics | .00 | .04 | .70 | .26 | .00 |  | 24094 | 11262 | 16598 |  |
| A | 411.5 | NA | NA | Who invented the Christmas Cracker? | Tom Smith | .00 | .00 | .82 | .16 | .02 |  |  | 5964 | 23231 | 29572 |
| A | 411.5 | NA | NA | What was raced in the first competitive event at the Indianapolis Motor Speedway? | Balloons | .00 | .04 | .47 | .49 | .00 |  | 11194 | 9738 | 16755 |  |
| B | 411.5 | NA | NA | What do you call the accumulation of earth and stones carried and finally deposited by a glacier? | Moraine | .00 | .19 | .52 | .30 | .00 |  | 15502 | 15028 | 16696 |  |
|  |  |  |  |  |  |  |  |  |  |  |  |  |  |  |  |
|  |  |  |  |  |  | CR (proportion of responses) | | | | | CR (response times) | | | | |
| Set | CR Rank | T. et al. rank | N & N rank | General Knowledge Question | Correct Answer | Correct | DR | DK | CE | OE | Correct | DR | DK | CE | OE |
| D | 411.5 | 246 | 194 | What is the name of the baseball player with the highest lifetime batting average in the major leagues? | Cobb | .00 | .08 | .24 | .68 | .00 |  | 31984 | 9219 | 17631 |  |
| D | 411.5 | 297 | 298 | What is the last name of the man who wrote the poem "It Couldn't Be Done"? | Guest | .00 | .06 | .92 | .02 | .00 |  | 8620 | 7164 | 17126 |  |

*Notes*: CR = cued-recall; T et al. = Tauber et al. (2013); N & N = Nelson and Narens (1980), CE = commission errors; DR = don't remember; DK = don't know; OE = omission errors

Table A2

*Multiple Choice Performance (Accuracy and Response Times)*

|  |  |  |  |  |  |  |  | MC  (response times) | | MC FOILS  (% of times selected as response) | | |  |
| --- | --- | --- | --- | --- | --- | --- | --- | --- | --- | --- | --- | --- | --- |
| Set | CR Rank | MC rank | T. et al. rank | N & N rank | Question | CORRECT ANSWER | Correct | Errors | Correct | Foil 1 | Foil 2 | Foil 3 | Difference (MC-CR) |
| B | 1 | 19 | NA | NA | Which band was Paul McCartney a member of? | The Beatles | .96 | 8795 | 4329 | The Monkees (2.0%) | The Rolling Stones (2.0%) | The Yardbirds (0.0%) | -.02 |
| B | 2 | 140 | NA | NA | What is the short pleated skirt worn by Scottish men? | Kilt | .78 | 5279 | 4757 | Glengarry (0.0%) | Kelt (20.4%) | Shendyt (2.0%) | -.19 |
| B | 3 | 2.5 | NA | NA | What is the hard, white material sourced from elephant tusks? | Ivory | 1.00 |  | 5265 | Enamel (0.0%) | Baleen (0.0%) | Porcelain (0.0%) | .06 |
| B | 4 | 50 | NA | NA | What word means to trade by exchanging goods for other goods rather than money? | Barter | .92 | 9128 | 7562 | Export (0.0%) | Haggle (2.0%) | Swap (6.1%) | -.01 |
| B | 5 | 50 | NA | NA | What is an airplane without an engine called? | Glider | .92 | 11400 | 5656 | Airship (2.0%) | Biplane (4.1%) | Prop (2.0%) | .03 |
| C | 6 | 50 | NA | NA | What is the term for the first aid instrument which stops the flow of blood in an artery preventing it from being lost through a wound? | Tourniquet | .92 | 15044 | 10482 | Bandage (4.1%) | Splint (2.0%) | Stabilizer (2.0%) | .04 |
|  |  |  |  |  |  |  |  | MC  (response times) | | MC FOILS  (% of times selected as response) | | |  |
| Set | CR Rank | MC rank | T. et al. rank | N & N rank | Question | CORRECT ANSWER | Correct | Errors | Correct | Foil 1 | Foil 2 | Foil 3 | Difference (MC-CR) |
| B | 7 | 74 | NA | NA | What is the name of the nylon fabric which has two pieces which stick to each other and is used as a fastener? | Velcro | .90 | 9350 | 7973 | Hook And Eye (10.2%) | Poppers (0.0%) | Toggles (0.0%) | .03 |
| C | 8.5 | 2.5 | NA | NA | What is the last name of the person who sang 'I did it my way'? | Sinatra | 1.00 |  | 6004 | Gaye (0.0%) | Holly (0.0%) | Humperdinck (0.0%) | .13 |
| C | 8.5 | 50 | NA | NA | What is the name of the song traditionally sung at the stroke of midnight on New Year's Eve? | Auld Lang Syne | .92 | 14585 | 5639 | Ave Maria (2.0%) | Happy Days Are Here Again (4.1%) | Memories (2.0%) | .05 |
| B | 11 | 19 | NA | NA | What is the last name of the boxer who later become known as Mohammed Ali? | Clay | .96 | 8616 | 11501 | Frazier (0.0%) | Lewis (0.0%) | Liston (4.1%) | .11 |
| B | 11 | 93 | NA | NA | What is the traditional daytime sleep in Spain? | Siesta | .88 | 7842 | 5355 | Despierto (0.0%) | Dormir (4.1%) | Fiesta (8.2%) | .03 |
| B | 11 | 110 | NA | NA | In what park is "Old Faithful" located? | Yellowstone | .84 | 9510 | 5935 | Glacier (0.0%) | Yosemite (14.3%) | Zion (2.0%) | -.02 |
| B | 14.5 | 19 | NA | NA | What is the name of the art of Japanese paper folding? | Origami | .96 | 3098 | 4605 | Amigurumi (0.0%) | Bonsai (2.0%) | Temari (2.0%) | .13 |
| B | 14.5 | 74 | NA | NA | Who was England's prime minister during World War II? | Churchill | .90 | 9592 | 6035 | Chamberlain (4.1%) | Roosevelt (4.1%) | Thatcher (2.0%) | .06 |
|  |  |  |  |  |  |  |  | MC  (response times) | | MC FOILS  (% of times selected as response) | | |  |
| Set | CR Rank | MC rank | T. et al. rank | N & N rank | Question | CORRECT ANSWER | Correct | Errors | Correct | Foil 1 | Foil 2 | Foil 3 | Difference (MC-CR) |
| B | 14.5 | 93 | NA | NA | What is the word "memo" short for? | Memorandum | .88 | 8958 | 4619 | Memoir (0.0%) | Memorable (0.0%) | Short Note (12.2%) | .04 |
| B | 14.5 | 212 | NA | NA | What is the colored portion of the eye? | Iris | .61 | 8771 | 6965 | Pupil (24.5%) | Retina (14.3%) | Sclera (0.0%) | -.22 |
| C | 17 | 50 | NA | NA | What is the name of the shell from which people hear the sea? | Conch | .92 | 17581 | 7569 | Clam (2.0%) | Jingle (0.0%) | Oyster (6.1%) | .10 |
| B | 18.5 | 2.5 | NA | NA | What do you call the vessel, usually an ornamental vase on a pedestal, which is used to preserve the ashes of the dead? | Urn | 1.00 | 7917 |  | Coffin (0.0%) | Shrine (0.0%) | Ewer (0.0%) | .19 |
| B | 18.5 | 19 | NA | NA | What do you call an instrument for performing calculations by sliding beads along rods or grooves? | Abacus | .96 | 12104 | 6341 | Caliper (2.0%) | Nomogram (0.0%) | Protractor (2.0%) | .14 |
| C | 20 | 50 | NA | NA | What device blows air on to a fire to make it hotter? | Bellows | .92 | 12967 | 6144 | Chute (2.0%) | Fan (4.1%) | Oscillating fan (2.0%) | .11 |
| B | 21 | 19 | NA | NA | What is the proper name for a "tidal wave"? | Tsunami | .96 | 6063 | 6490 | Hurricane (2.0%) | Typhoon (0.0%) | Whirlpool (2.0%) | .16 |
| C | 22.5 | 33 | NA | NA | What is a goat's offspring called? | Kid | .94 | 7179 | 4601 | Colt (0.0%) | Cub (0.0%) | Doe (6.1%) | .15 |
|  |  |  |  |  |  |  |  |  |  |  |  |  |  |
|  |  |  |  |  |  |  |  | MC  (response times) | | MC FOILS  (% of times selected as response) | | |  |
| Set | CR Rank | MC rank | T. et al. rank | N & N rank | Question | CORRECT ANSWER | Correct | Errors | Correct | Foil 1 | Foil 2 | Foil 3 | Difference (MC-CR) |
| C | 22.5 | 33 | NA | NA | What optical instrument allows crews of submerged submarines to look at surface ships? | Periscope | .94 | 6138 | 8395 | Macroscope (0.0%) | Oscilloscope (2.0%) | Telescope (4.1%) | .15 |
| B | 25.5 | 6.5 | NA | NA | What is the term for nautical mile per hour? | Knot | .98 | 22652 | 4516 | Horsepower (0.0%) | Rate (0.0%) | Speed (2.0%) | .20 |
| B | 25.5 | 33 | NA | NA | What is the name of the loch (lake) in Scotland that is supposedly home of a legendary monster? | Ness | .94 | 7955 | 7740 | Lochy (2.0%) | Lomond (4.1%) | Oich (0.0%) | .16 |
| B | 25.5 | 50 | NA | NA | What is the name of the Egyptian plant that is cut into strips and pressed into a material to write on? | Papyrus | .92 | 13027 | 8070 | Birch (2.0%) | Flax (2.0%) | Hemp (4.1%) | .14 |
| B | 25.5 | 293 | NA | NA | Which video game character is a plumber? | Super Mario | .41 | 7941 | 6091 | Luigi (46.9%) | Pikachu (0.0%) | Sponge Bob (12.2%) | -.37 |
| C | 28 | 93 | NA | NA | What is the craft of tying knots to make belts, bags, and plant hangers? | Macrame | .88 | 18343 | 6887 | Crochet (6.1%) | Knotting (4.1%) | Stitchery (2.0%) | .12 |
| D | 29 | 165 | 189 | 157 | What is the last name of the doctor who first developed a vaccine against polio? | Salk | .73 | 8038 | 5886 | Fleming (12.2%) | Pauling (6.1%) | Sabin (8.2%) | -.02 |
|  |  |  |  |  |  |  |  | MC  (response times) | | MC FOILS  (% of times selected as response) | | |  |
| Set | CR Rank | MC rank | T. et al. rank | N & N rank | Question | CORRECT ANSWER | Correct | Errors | Correct | Foil 1 | Foil 2 | Foil 3 | Difference (MC-CR) |
| C | 31 | 19 | NA | NA | What is the name of the hillbilly family who had the famous feud with the McCoys? | Hatfield | .96 | 37352 | 6025 | Ferguson (2.0%) | Hickok (0.0%) | McDaniel (2.0%) | .21 |
| C | 31 | 33 | NA | NA | What is the name of the man who created the comic strip 'Peanuts'? | Shultz | .94 | 9256 | 5462 | Goodman (2.0%) | Keene (4.1%) | Rogers (0.0%) | .19 |
| C | 31 | 74 | NA | NA | In mammals the period of time between fertilization of the egg and the birth of the young is known by what scientific term? | Gestation | .90 | 13586 | 12733 | Blastulation (2.0%) | Conception (4.1%) | Germination (4.1%) | .15 |
| B | 33 | 235 | NA | NA | Which was the 49th state to join the Union? | Alaska | .55 | 12739 | 8648 | Arizona (2.0%) | Hawaii (36.7%) | New Mexico (6.1%) | -.19 |
| C | 34.5 | 93 | NA | NA | What is the term frequently applied to Christ's doctrine of doing to others as we would wish them to do unto us? | Golden Rule | .88 | 10797 | 9511 | Apostle's Creed (10.2%) | Beatitude (0.0%) | Sermon on the Mount (2.0%) | .15 |
| C | 34.5 | 93 | NA | NA | What was the name given to the geographic line in the United States which was the separation of slavery and freedom? | Mason-Dixon Line | .88 | 11110 | 10175 | Abolition Divide (2.0%) | Freedom Void (2.0%) | Great Divide (8.2%) | .15 |
|  |  |  |  |  |  |  |  | MC  (response times) | | MC FOILS  (% of times selected as response) | | |  |
| Set | CR Rank | MC rank | T. et al. rank | N & N rank | Question | CORRECT ANSWER | Correct | Errors | Correct | Foil 1 | Foil 2 | Foil 3 | Difference (MC-CR) |
| D | 36 | 110 | 181 | 150 | Of which country is Budapest the capital? | Hungary | .84 | 9104 | 6739 | Austria (0.0%) | Croatia (0.0%) | Romania (16.3%) | .11 |
| B | 38.5 | 19 | NA | NA | What do you call a question that is asked for effect with no answer expected? | Rhetorical | .96 | 14244 | 6917 | Euphemism (0.0%) | Literal (2.0%) | Metaphor (2.0%) | .24 |
| B | 38.5 | 50 | NA | NA | What word means to formally renounce a throne? | Abdicate | .92 | 5373 | 5616 | Abjure (0.0%) | Presides (0.0%) | Recant (8.2%) | .20 |
| B | 38.5 | 93 | NA | NA | Which president suffered from polio? | Roosevelt | .88 | 12159 | 6298 | Kennedy (0.0%) | Truman (6.1%) | Wilson (6.1%) | .16 |
| B | 38.5 | 103 | NA | NA | What do you call a savory sauce in which meat, fish, or a vegetable is soaked before cooking to enhance the flavor? | Marinade | .86 | 14333 | 7573 | Brine (12.2%) | Gravy (0.0%) | Stew (2.0%) | .13 |
| C | 41.5 | 50 | NA | NA | What is the term for someone who doubts but does not deny the existence of God? | Agnostic | .92 | 12228 | 9858 | Apathetic (2.0%) | Apostolic (0.0%) | Atheist (6.1%) | .20 |
| C | 41.5 | 140 | NA | NA | What was the name of the body of water where Thoreau studied for two years? | Walden Pond | .78 | 9972 | 9595 | Golden Pond (8.2%) | Lake Champlain (4.1%) | Lake Huron (10.2%) | .06 |
|  |  |  |  |  |  |  |  |  |  |  |  |  |  |
|  |  |  |  |  |  |  |  | MC  (response times) | | MC FOILS  (% of times selected as response) | | |  |
| Set | CR Rank | MC rank | T. et al. rank | N & N rank | Question | CORRECT ANSWER | Correct | Errors | Correct | Foil 1 | Foil 2 | Foil 3 | Difference (MC-CR) |
| B | 44 | 74 | NA | NA | Where was the Declaration of Independence signed? | Philadelphia | .90 | 8001 | 5392 | Boston (2.0%) | Charlottesville (0.0%) | Washington (8.2%) | .19 |
| B | 44 | 93 | NA | NA | What word means to shed hair, feathers, or an outer layer periodically, as with the changing seasons? | Molt | .88 | 10987 | 7331 | Divest (2.0%) | Flake (4.1%) | Renew (6.1%) | .17 |
| B | 44 | 123 | NA | NA | What is the musical term that means "without instrumental accompaniment"? | A capella | .82 | 8294 | 5816 | Acoustic (12.2%) | Harmony (2.0%) | Solo (4.1%) | .11 |
| C | 47.5 | 33 | NA | NA | What do we call the bone which composes the lower jaw? | Mandible | .94 | 10390 | 8052 | Femur (0.0%) | Goman Arch (2.0%) | Maxilla (4.1%) | .24 |
| C | 47.5 | 50 | NA | NA | What is the last name of the first female pilot to cross the Atlantic? | Earhart | .92 | 6816 | 8548 | Johnson (0.0%) | Lindbergh (8.2%) | Rickenbacker (0.0%) | .22 |
| C | 47.5 | 151 | NA | NA | What nickname was given to Northerners who went south after the Civil War and took advantage of Southern poverty? | Carpetbaggers | .76 | 16136 | 9464 | Reconstructionists (8.2%) | Scallywags (14.3%) | Tories (2.0%) | .05 |
|  |  |  |  |  |  |  |  | MC  (response times) | | MC FOILS  (% of times selected as response) | | |  |
| Set | CR Rank | MC rank | T. et al. rank | N & N rank | Question | CORRECT ANSWER | Correct | Errors | Correct | Foil 1 | Foil 2 | Foil 3 | Difference (MC-CR) |
| C | 47.5 | 151 | NA | NA | What was the last name of the author who wrote the fable about the fox who assumed the grapes he couldn't reach were sour anyway? | Aesop | .76 | 12472 | 11147 | Grimm (18.4%) | James (4.1%) | Rhein (2.0%) | .05 |
| C | 50 | 50 | NA | NA | What are the wooden clappers called that Spanish dancers hold in their hands? | Castanets | .92 | 9868 | 7328 | Caracas (6.1%) | Cymbals (0.0%) | Glockenspiels (2.0%) | .23 |
| C | 51 | 50 | NA | NA | What was the name of the vehicle used to carry and cook food on cattle drives? | Chuckwagon | .92 | 11546 | 8876 | Covered Wagon (2.0%) | Foodwagon (4.1%) | Grubwagon (2.0%) | .25 |
| B | 53 | 19 | NA | NA | What are people who make maps called? | Cartographers | .96 | 7007 | 5430 | Drafters (0.0%) | Geographers (4.1%) | Mappers (0.0%) | .29 |
| B | 53 | 74 | NA | NA | What is the unit that marks sound intensity? | Decibel | .90 | 9195 | 7093 | Hertz (4.1%) | Volume (6.1%) | Watt (0.0%) | .23 |
| B | 53 | 186 | NA | NA | What is the front section of a boat? | Bow | .67 | 7501 | 8830 | Port (2.0%) | Starboard (4.1%) | Stern (26.5%) | .01 |
| C | 55.5 | 19 | NA | NA | Humans are classified as what species? | Sapiens | .96 | 4855 | 6232 | Erectus (4.1%) | Habilis (0.0%) | Robustus (0.0%) | .30 |
| C | 55.5 | 19 | NA | NA | What is the name of the palace in London in which the Monarch of England resides? | Buckingham | .96 | 9922 | 8438 | Balmoral (0.0%) | Elsinore (0.0%) | Kensington (4.1%) | .30 |
|  |  |  |  |  |  |  |  | MC  (response times) | | MC FOILS  (% of times selected as response) | | |  |
| Set | CR Rank | MC rank | T. et al. rank | N & N rank | Question | CORRECT ANSWER | Correct | Errors | Correct | Foil 1 | Foil 2 | Foil 3 | Difference (MC-CR) |
| B | 61.5 | 19 | NA | NA | What is the resort city on the French Riviera between Nice and St. Tropez where a famous film festival is held? | Cannes | .96 | 12719 | 7182 | Antibes (0.0%) | Paris (2.0%) | Venice (2.0%) | .31 |
| B | 61.5 | 50 | NA | NA | What do you call a feeling of resentment, often at some fancied slight or insult? (usually follows the verb "take") | Umbrage | .47 | 13952 | 9443 | Indignation (12.2%) | Offense (38.8%) | Wrath (2.0%) | .23 |
| B | 61.5 | 50 | NA | NA | What is the name of a mixture of dried spices and flowers used for perfuming a room? | Potpourri | .92 | 9690 | 7809 | Bouquet (0.0%) | Incense (4.1%) | Sachet (4.1%) | .27 |
| B | 61.5 | 50 | NA | NA | What is the order of lower mammals including kangaroos and opossums which carry their young in an abdominal pouch? | Marsupialia | .92 | 15734 | 7603 | Carnivora (0.0%) | Dermoptera (2.0%) | Tubulidentata (6.1%) | .27 |
| B | 61.5 | 50 | NA | NA | Who is the artist that painted everyday objects such as soup cans? | Warhol | .92 | 7946 | 5760 | Dali (6.1%) | Monet (0.0%) | Picasso (2.0%) | .27 |
|  |  |  |  |  |  |  |  |  |  |  |  |  |  |
|  |  |  |  |  |  |  |  | MC  (response times) | | MC FOILS  (% of times selected as response) | | |  |
| Set | CR Rank | MC rank | T. et al. rank | N & N rank | Question | CORRECT ANSWER | Correct | Errors | Correct | Foil 1 | Foil 2 | Foil 3 | Difference (MC-CR) |
| B | 61.5 | 74 | NA | NA | What do you call a plant material such as straw used as a roofing material for a house? | Thatch | .90 | 15687 | 7090 | Hatch (4.1%) | Reed (4.1%) | Weave (2.0%) | .25 |
| B | 61.5 | 74 | NA | NA | What is the name of the unrhymed verse form of Japanese origin having three lines containing usually 5, 7, and 5 syllables? | Haiku | .90 | 14914 | 6774 | Kabuki (10.2%) | Senryu (0.0%) | Tanka (0.0%) | .25 |
| B | 61.5 | 151 | NA | NA | What is the capital of Thailand? | Bangkok | .76 | 10059 | 6891 | Beijing (0.0%) | Hanoi (10.2%) | Phnom Penh (14.3%) | .11 |
| B | 61.5 | 175 | NA | NA | Who is the oldest sister in "The Brady Bunch"? | Marcia | .71 | 6724 | 6058 | Cindy (6.1%) | Jan (18.4%) | Martha (4.1%) | .07 |
| B | 61.5 | 181 | NA | NA | What word means to divide an area into electoral district in order to give special advantage to one political party? | Gerrymander | .69 | 12869 | 9094 | Disenfranchise (2.0%) | Logrolling (4.1%) | Redistricting (24.5%) | .05 |
| C | 68.5 | 19 | NA | NA | What is the fin on the back of a fish called? | Dorsal | .96 | 6471 | 6161 | Back (0.0%) | Front (0.0%) | Temporal (4.1%) | .32 |
| C | 68.5 | 19 | NA | NA | What mammal has armor-like bony plates as its most distinguishing feature? | Armadillo | .96 | 8966 | 8400 | Aardvark (0.0%) | Anteater (2.0%) | Platypus (2.0%) | .32 |
|  |  |  |  |  |  |  |  | MC  (response times) | | MC FOILS  (% of times selected as response) | | |  |
| Set | CR Rank | MC rank | T. et al. rank | N & N rank | Question | CORRECT ANSWER | Correct | Errors | Correct | Foil 1 | Foil 2 | Foil 3 | Difference (MC-CR) |
| C | 68.5 | 151 | NA | NA | What did the Seven Dwarves do for a living? | Mining | .76 | 10484 | 5929 | Carpentry (16.3%) | Farming (2.0%) | Hunting (6.1%) | .11 |
| C | 68.5 | 186 | NA | NA | What river separates Washington D.C. from Virginia? | Potomac | .67 | 10098 | 8958 | Delaware (18.4%) | Hudson (10.2%) | Missouri (4.1%) | .03 |
| B | 71 | 50 | NA | NA | What do you call a member of a volunteer group organized to suppress and punish crime (as when legal processes seem inadequate)? | Vigilante | .92 | 17493 | 12511 | Defender (2.0%) | Gang (4.1%) | Patrol (2.0%) | .29 |
| C | 74 | 19 | NA | NA | What instrument is used to measure earthquakes? | Seismograph | .96 | 8941 | 5496 | Altimeter (0.0%) | Oscilloscope (0.0%) | Richtograph (4.1%) | .33 |
| C | 74 | 74 | NA | NA | What was the land where Puff the Magic Dragon lives? | Honalee | .90 | 8535 | 6482 | Amber (2.0%) | Middle Earth (0.0%) | Never-Never Land (8.2%) | .27 |
| C | 74 | 110 | NA | NA | What sailor's disease resulted from a deficiency in vitamin C? | Scurvy | .84 | 12199 | 7433 | Polio (0.0%) | Rickets (12.2%) | Scarlet Fever (4.1%) | .21 |
| C | 74 | 123 | NA | NA | What is the technical term for the phenomenon commonly known as the Northern Lights? | Aurora Borealis | .82 | 7423 | 7279 | Aurora Australis (12.2%) | Polar Halo (2.0%) | Polar Lights (4.1%) | .19 |
|  |  |  |  |  |  |  |  |  |  |  |  |  |  |
|  |  |  |  |  |  |  |  | MC  (response times) | | MC FOILS  (% of times selected as response) | | |  |
| Set | CR Rank | MC rank | T. et al. rank | N & N rank | Question | CORRECT ANSWER | Correct | Errors | Correct | Foil 1 | Foil 2 | Foil 3 | Difference (MC-CR) |
| C | 74 | 123 | NA | NA | What type of paper is commonly used as an acid-base indicator in chemistry classes? | Litmus | .82 | 12067 | 6824 | Barium (12.2%) | Chromatin Red (0.0%) | Chromium (6.1%) | .19 |
| C | 77 | 93 | NA | NA | What is the name of the short sword fastened to the end of a musket or rifle? | Bayonet | .88 | 10067 | 6921 | Cutlass (0.0%) | Dagger (8.2%) | Dirk (4.1%) | .27 |
| B | 78 | 50 | NA | NA | Where is the Amazon rainforest located? | Brazil | .92 | 5178 | 5399 | Columbia (4.1%) | Seattle (0.0%) | Venezuela (4.1%) | .31 |
| C | 80 | 19 | NA | NA | What device is used to measure levels of radioactivity? | Geiger Counter | .96 | 6739 | 6724 | Hertz Counter (0.0%) | Ohm Receiver (2.0%) | Radiometer (2.0%) | .36 |
| C | 80 | 33 | NA | NA | Who was the magician and prophet from King Arthur's era? | Merlin | .94 | 6610 | 7312 | Loki (0.0%) | Mephistopheles (6.1%) | Mordred (0.0%) | .34 |
|  |  |  |  |  |  |  |  |  |  |  |  |  |  |
|  |  |  |  |  |  |  |  |  |  |  |  |  |  |
|  |  |  |  |  |  |  |  | MC  (response times) | | MC FOILS  (% of times selected as response) | | |  |
| Set | CR Rank | MC rank | T. et al. rank | N & N rank | Question | CORRECT ANSWER | Correct | Errors | Correct | Foil 1 | Foil 2 | Foil 3 | Difference (MC-CR) |
| C | 80 | 93 | NA | NA | What was the name of the disorder depicted by Dustin Hoffman's character in the movie 'Rainman' otherwise known as Idiot Savantism? | Autism | .88 | 20036 | 9899 | Down Syndrome (6.1%) | Dyslexia (4.1%) | Paranoia (2.0%) | .28 |
| A | 82.5 | 74 | NA | NA | What does VoIP stand for? | Voice over Internet Protocol | .90 | 24570 | 8633 | Vision of Interest Piece (0.0%) | Vivendi operation Internet Protocol (4.1%) | Vocal or Internet Program (6.1%) | .30 |
| A | 82.5 | 165 | NA | NA | Who defeated Pres. Harry Truman in the 1948 election according to an infamous Chicago Tribune headline? | Thomas Dewey | .73 | 19665 | 7604 | Alben W. Barkley (4.1%) | Dwight D. Eisenhower (20.4%) | Earl Warren (2.0%) | .14 |
| B | 86 | 74 | NA | NA | What is the name of the migratory grasshopper that travels in vast swarms and strips areas passed of all vegetation? | Locust | .90 | 14857 | 8730 | Cicada (10.2%) | Katydid (0.0%) | Weevil (0.0%) | .31 |
| B | 86 | 123 | NA | NA | What city is the capital of Iceland? | Reykjavik | .82 | 5944 | 5262 | Arborg (12.2%) | Kopavogur (4.1%) | Reykjanesbaer (2.0%) | .22 |
| B | 86 | 165 | NA | NA | Who was the first female Supreme Court justice? | O'Connor | .73 | 6489 | 7370 | Ginsburg (20.4%) | Kagan (0.0%) | Sotomayor (6.1%) | .14 |
|  |  |  |  |  |  |  |  | MC  (response times) | | MC FOILS  (% of times selected as response) | | |  |
| Set | CR Rank | MC rank | T. et al. rank | N & N rank | Question | CORRECT ANSWER | Correct | Errors | Correct | Foil 1 | Foil 2 | Foil 3 | Difference (MC-CR) |
| B | 86 | 172 | NA | NA | Who was the composer who worked with deafness? | Beethoven | .71 | 7512 | 7783 | Bach (4.1%) | Chopin (4.1%) | Mozart (20.4%) | .12 |
| B | 86 | 254 | NA | NA | What is the last name of the man who assassinated Robert Kennedy? | Sirhan | .51 | 5856 | 9892 | Oswald (42.9%) | Ray (2.0%) | Ruby (4.1%) | -.08 |
| C | 89 | 151 | NA | NA | What is a community of ants called? | Colony | .76 | 7431 | 7512 | Farm (12.2%) | Hill (12.2%) | Hive (0.0%) | .17 |
| B | 91.5 | 19 | NA | NA | What is the last name of the actor who played Perry Mason on TV? | Burr | .96 | 5686 | 5020 | Arness (2.0%) | Connors (2.0%) | Stevens (0.0%) | .39 |
| B | 91.5 | 74 | NA | NA | What do you call a stone building (often found in cemetery) with places for entombment of the dead above ground? | Mausoleum | .90 | 10666 | 9197 | Catacomb (0.0%) | Crematorium (0.0%) | Crypt (10.2%) | .32 |
| B | 91.5 | 74 | NA | NA | What is the former name of Istanbul, which was used when the city was occupied by Christians? | Constantinople | .90 | 12450 | 9327 | Ankara (6.1%) | Prussia (2.0%) | Stalinabad (2.0%) | .32 |
| B | 91.5 | 262 | NA | NA | What was the last name of Lucille Ball's first husband, who starred with her on "I Love Lucy"? | Arnaz | .49 | 6778 | 8099 | Mertz (0.0%) | Ricardo (51.0%) | Santiago (0.0%) | -.08 |
|  |  |  |  |  |  |  |  | MC  (response times) | | MC FOILS  (% of times selected as response) | | |  |
| Set | CR Rank | MC rank | T. et al. rank | N & N rank | Question | CORRECT ANSWER | Correct | Errors | Correct | Foil 1 | Foil 2 | Foil 3 | Difference (MC-CR) |
| C | 96 | 2.5 | NA | NA | What is the name of the three leaf clover which is the emblem of Ireland? | Shamrock | 1.00 |  | 7451 | Blarney (0.0%) | Heather (0.0%) | Lavender (0.0%) | .43 |
| C | 96 | 93 | NA | NA | What was the name of the infamous American traitor in the Revolutionary War? | Arnold | .88 | 7611 | 7457 | Brown (0.0%) | Jackson (10.2%) | Johnson (2.0%) | .31 |
| C | 96 | 110 | NA | NA | What is a group of geese called? | Gaggle | .84 | 7645 | 6459 | Flock (12.2%) | Gathering (2.0%) | Herd (2.0%) | .27 |
| C | 96 | 134 | NA | NA | What is a young female horse called? | Filly | .80 | 10229 | 6690 | Colt (6.1%) | Foal (14.3%) | Gelding (0.0%) | .23 |
| C | 96 | 151 | NA | NA | What is the name of the disease of the liver caused by abnormally high levels of bile in the blood that gives the skin a yellow color? | Jaundice | .76 | 11352 | 8778 | Diabetes (0.0%) | Hepatitis (24.5%) | Korsakoff (0.0%) | .19 |
| D | 99 | 165 | 190 | 181 | What is the last name of the man who began the Reformation in Germany? | Luther | .73 | 13529 | 6590 | Calvin (8.2%) | Hus (12.2%) | Zwingli (6.1%) | .17 |
|  |  |  |  |  |  |  |  |  |  |  |  |  |  |
|  |  |  |  |  |  |  |  |  |  |  |  |  |  |
|  |  |  |  |  |  |  |  | MC  (response times) | | MC FOILS  (% of times selected as response) | | |  |
| Set | CR Rank | MC rank | T. et al. rank | N & N rank | Question | CORRECT ANSWER | Correct | Errors | Correct | Foil 1 | Foil 2 | Foil 3 | Difference (MC-CR) |
| B | 101 | 50 | NA | NA | What is the name of the islands off the coast of Ecuador that Darwin visited to study unique species of birds and animals? | Galapagos | .92 | 12333 | 6770 | Easter (4.1%) | Isla De San Carlos (4.1%) | Malpelo (0.0%) | .36 |
| B | 101 | 50 | NA | NA | What is the word that means to cause to explode or to set off, for example: a bomb? | Detonate | .92 | 8338 | 8213 | Diffuse (0.0%) | Implode (0.0%) | Trigger (8.2%) | .36 |
| B | 101 | 254 | NA | NA | What is the orbiting particle of an atom? | Electron | .51 | 9099 | 8669 | Neutron (26.5%) | Nucleus (6.1%) | Proton (16.3%) | -.05 |
| C | 104 | 33 | NA | NA | What was the last name of the female mouseketeer who later appeared in commercials for peanut butter? | Funicello | .94 | 11490 | 9613 | Duke (2.0%) | Lopez (2.0%) | McCormick (2.0%) | .39 |
| C | 104 | 74 | NA | NA | What is the famous prehistoric structure situated on Salisbury Plain England? | Stonehenge | .90 | 8031 | 12008 | Blarney Stone (6.1%) | Druid Ruins (0.0%) | Easter Statues (4.1%) | .35 |
| C | 104 | 123 | NA | NA | What seasonal South Asian wind is characterized by heavy rains? | Monsoon | .82 | 11762 | 9503 | Hurricane (4.1%) | Trade Wind (8.2%) | Tsunami (6.1%) | .26 |
|  |  |  |  |  |  |  |  | MC  (response times) | | MC FOILS  (% of times selected as response) | | |  |
| Set | CR Rank | MC rank | T. et al. rank | N & N rank | Question | CORRECT ANSWER | Correct | Errors | Correct | Foil 1 | Foil 2 | Foil 3 | Difference (MC-CR) |
| C | 107 | 50 | NA | NA | What is the name of the legendary race of female warriors? | Amazons | .92 | 10899 | 5463 | Dryads (0.0%) | Nillians (4.1%) | Titans (4.1%) | .38 |
| C | 107 | 50 | NA | NA | What is the wand called which is held in the hand as a symbol of regal or imperial power? | Scepter | .92 | 15944 | 11860 | Baton (2.0%) | Javelin (2.0%) | Staff (4.1%) | .38 |
| C | 107 | 74 | NA | NA | What was the name of the medieval code of honor practiced by knights that among other things elevated the position of women? | Chivalry | .90 | 19838 | 9189 | Feudalism (2.0%) | Gentility (8.2%) | Grade (0.0%) | .36 |
| B | 110 | 175 | NA | NA | Who said the phrase, "To be or not to be"? | Hamlet | .71 | 9565 | 7962 | Caesar (8.2%) | Macbeth (16.3%) | Romeo (4.1%) | .18 |
| B | 110 | 235 | NA | NA | What is a group of lines in a poem called? | Stanza | .55 | 8123 | 6231 | Paragraph (0.0%) | Prose (6.1%) | Verse (38.8%) | .01 |
| D | 112 | 123 | 168 | 221 | Over which river is the George Washington Bridge? | Hudson | .82 | 8310 | 9203 | East (14.3%) | Hackensack (2.0%) | Harlem (2.0%) | .29 |
| D | 112 | 134 | 179 | 183 | What is the last name of the actor who received the Best Actor award for the movie "On The Waterfront"? | Brando | .80 | 8671 | 6131 | Holden (12.2%) | Newman (8.2%) | Niven (0.0%) | .27 |
|  |  |  |  |  |  |  |  | MC  (response times) | | MC FOILS  (% of times selected as response) | | |  |
| Set | CR Rank | MC rank | T. et al. rank | N & N rank | Question | CORRECT ANSWER | Correct | Errors | Correct | Foil 1 | Foil 2 | Foil 3 | Difference (MC-CR) |
| C | 114 | 93 | NA | NA | What was the location of George Washington's encampment where his men suffered every conceivable hardship from 1777-1778? | Valley Forge | .88 | 13607 | 9782 | Antietam (2.0%) | Bull Run (0.0%) | Gettysburg (10.2%) | .36 |
| C | 114 | 140 | NA | NA | What was the last name of the nearly blind impressionist artist who painted a series of mural-sized pictures of water lilies near the end of his life? | Monet | .78 | 16918 | 14507 | Cezanne (8.2%) | Manet (4.1%) | Renoir (10.2%) | .25 |
| C | 114 | 151 | NA | NA | What dwarf kills himself when a young bride guesses his highly unusual name? | Rumpelstiltskin | .76 | 17771 | 8979 | Galnon (6.1%) | Quasimodo (18.4%) | Rip Van Winkle (0.0%) | .23 |
| B | 117 | 6.5 | NA | NA | What word means to bleed heavily or uncontrollably? | Hemorrhage | .98 | 24852 | 5888 | Coagulation (2.0%) | Hematemesis (0.0%) | Imbrue (0.0%) | .46 |
| B | 117 | 134 | NA | NA | What is the name of the strait between Alaska and Siberia? | Bering | .80 | 6640 | 6419 | Dover (0.0%) | Gibraltar (20.4%) | Malacca (0.0%) | .28 |
| B | 117 | 225 | NA | NA | What is the last name of the current mayor of New York City? | De Blasio | .57 | 8316 | 6245 | Bloomberg (18.4%) | Cuomo (14.3%) | Giuliani (10.2%) | .05 |
|  |  |  |  |  |  |  |  | MC  (response times) | | MC FOILS  (% of times selected as response) | | |  |
| Set | CR Rank | MC rank | T. et al. rank | N & N rank | Question | CORRECT ANSWER | Correct | Errors | Correct | Foil 1 | Foil 2 | Foil 3 | Difference (MC-CR) |
| C | 120 | 123 | NA | NA | What beetle was held sacred by ancient Egyptians? | Scarab | .82 | 12822 | 5823 | Black (2.0%) | Emerald (8.2%) | Japanese (8.2%) | .31 |
| C | 120 | 186 | NA | NA | What river in Hades does Charon ferry dead souls across? | Styx | .67 | 11225 | 10502 | Black (10.2%) | Death (10.2%) | Hades (12.2%) | .17 |
| C | 121 | 19 | NA | NA | Who shot an apple off of his son's head in the 14th century? | William Tell | .96 | 12187 | 5999 | King Arthur (2.0%) | St. Augustine (0.0%) | Sir Lancelot (2.0%) | .47 |
| B | 123 | 110 | NA | NA | What is the last name of the author of Little Women? | Alcott | .84 | 6086 | 6006 | Austen (10.2%) | Montgomery (2.0%) | Wharton (4.1%) | .36 |
| B | 123 | 181 | NA | NA | What is last name of the woman who wrote Gone With the Wind? | Mitchell | .69 | 7828 | 5939 | Atwood (4.1%) | Lee (8.2%) | O'Hara (18.4%) | .21 |
| C | 124 | 74 | NA | NA | What is the last name of the author of 'The Hobbit'? | Tolkien | .90 | 8955 | 5033 | Asimov (2.0%) | Castaneda (2.0%) | Lewis (6.1%) | .42 |
| A | 125 | 123 | NA | NA | What did the Wright Brothers do before inventing an aircraft? | Built bicycles | .82 | 15885 | 8616 | Designed buildings (6.1%) | Taught school (2.0%) | Wrote for a newspaper (10.2%) | .34 |
| B | 128 | 103 | NA | NA | What is the last name of the actress who played Gloria on TV's "All in the Family"? | Struthers | .86 | 9236 | 5778 | Anderson (4.1%) | Dickinson (0.0%) | Stapleton (10.2%) | .39 |
|  |  |  |  |  |  |  |  | MC  (response times) | | MC FOILS  (% of times selected as response) | | |  |
| Set | CR Rank | MC rank | T. et al. rank | N & N rank | Question | CORRECT ANSWER | Correct | Errors | Correct | Foil 1 | Foil 2 | Foil 3 | Difference (MC-CR) |
| B | 128 | 123 | NA | NA | What is the last name of the actor who starred in both the Broadway production and the film, "A Streetcar Named Desire"? | Brando | .82 | 11825 | 8343 | Dean (4.1%) | McQueen (8.2%) | Newman (6.1%) | .35 |
| B | 128 | 134 | NA | NA | What is the last name of the Romanian gymnast who scored 7 perfect "10's" and won 3 gold medals in the 1976 Olympics? | Comaneci | .80 | 21237 | 10355 | Amanar (0.0%) | Korbut (16.3%) | Latynina (4.1%) | .33 |
| B | 128 | 140 | NA | NA | What do you call a habitual spasmodic motion or twitching of particular muscles, especially in the face? | Tic | .78 | 8495 | 8367 | Contraction (2.0%) | Jerk (2.0%) | Twitch (18.4%) | .31 |
| B | 128 | 151 | NA | NA | What is the river that flows north from Switzerland to Holland and forms the border between Germany and France? | Rhine | .76 | 13557 | 10218 | Elbe (4.1%) | Loire (6.1%) | Seine (14.3%) | .29 |
|  |  |  |  |  |  |  |  |  |  |  |  |  |  |
|  |  |  |  |  |  |  |  | MC  (response times) | | MC FOILS  (% of times selected as response) | | |  |
| Set | CR Rank | MC rank | T. et al. rank | N & N rank | Question | CORRECT ANSWER | Correct | Errors | Correct | Foil 1 | Foil 2 | Foil 3 | Difference (MC-CR) |
| C | 131 | 186 | NA | NA | What was the harp-like instrument used in ancient Greece to accompany singing and recitation? | Lyre | .67 | 14898 | 11422 | Dulcimer (6.1%) | Lute (16.3%) | Mandolin (10.2%) | .21 |
| C | 134 | 19 | NA | NA | What was the name of the zeppelin, blimp, that exploded in Lake Hurst, New Jersey in 1937? | Hindenburg | .96 | 17616 | 7367 | Graph (2.0%) | Ludendorf (0.0%) | Mannheim (2.0%) | .51 |
| C | 134 | 74 | NA | NA | What is a male witch called? | Warlock | .90 | 14146 | 6384 | Magician (2.0%) | Sorcerer (8.2%) | Witch (0.0%) | .45 |
| C | 134 | 93 | NA | NA | What is the first name of the school teacher who was chased by the headless horseman in 'The Legend of Sleepy Hollow'? | Ichabod | .88 | 17775 | 8573 | Ethan (2.0%) | Oliver (6.1%) | Pip (4.1%) | .43 |
| C | 134 | 181 | NA | NA | What is the last name of the author of 'Uncle Tom's Cabin'? | Stowe | .69 | 8749 | 7604 | Christie (2.0%) | Keyes (8.2%) | Mitchell (20.4%) | .25 |
|  |  |  |  |  |  |  |  |  |  |  |  |  |  |
|  |  |  |  |  |  |  |  | MC  (response times) | | MC FOILS  (% of times selected as response) | | |  |
| Set | CR Rank | MC rank | T. et al. rank | N & N rank | Question | CORRECT ANSWER | Correct | Errors | Correct | Foil 1 | Foil 2 | Foil 3 | Difference (MC-CR) |
| C | 134 | 212 | NA | NA | What was the last name of Scrooge's dead partner in Dickens' 'A Christmas Carol?' | Marley | .61 | 11086 | 11499 | Copperfield (6.1%) | Cratchit (32.7%) | Nickleby (0.0%) | .16 |
| B | 138 | 123 | NA | NA | What is the term for when three musical notes are played together? | Chord | .82 | 6590 | 6585 | Melody (12.2%) | Octave (0.0%) | Scale (6.1%) | .37 |
| B | 138 | 140 | NA | NA | What do you call the three periods(…) used to indicate an omission or a pause? | Ellipsis | .78 | 9325 | 7517 | Allusion (0.0%) | Elliptical (4.1%) | Et Cetera (18.4%) | .33 |
| B | 138 | 316 | NA | NA | What word would means having a healthy reddish color? | Ruddy | .35 | 9822 | 10877 | Blush (12.2%) | Rosy (53.1%) | Sallow (0.0%) | -.10 |
| D | 140 | 123 | 175 | 192 | What is the last name of the author who wrote the Sherlock Holmes stories? | Doyle | .82 | 9589 | 5769 | Brett (6.1%) | Stevenson (8.2%) | Stoker (4.1%) | .38 |
| A | 142 | 245 | NA | NA | What game was Deep Blue skilled at? | Chess | .53 | 10263 | 7459 | Backgammon (26.5%) | Checkers (8.2%) | Dominoes (12.2%) | .09 |
| A | 142 | 282 | NA | NA | Who was Time Magazine's 1938 "Man of the Year"? | Adolph Hitler | .43 | 15920 | 9000 | Chiang Kai-shek (12.2%) | Franklin D. Roosevelt (26.5%) | Winston Churchill (18.4%) | -.01 |
| C | 144 | 93 | NA | NA | Who was the fictional character who married his mother and blinded himself? | Oedipus | .88 | 14465 | 9838 | Jason (2.0%) | Medea (4.1%) | Sophocles (6.1%) | .44 |
|  |  |  |  |  |  |  |  | MC  (response times) | | MC FOILS  (% of times selected as response) | | |  |
| Set | CR Rank | MC rank | T. et al. rank | N & N rank | Question | CORRECT ANSWER | Correct | Errors | Correct | Foil 1 | Foil 2 | Foil 3 | Difference (MC-CR) |
| C | 144 | 110 | NA | NA | What was the name of the German Secret State Police? | Gestapo | .84 | 14265 | 7871 | KGB (8.2%) | Nazis (8.2%) | Schutz (0.0%) | .40 |
| C | 144 | 134 | NA | NA | What peace treaty ended World War I? | Versailles | .80 | 9158 | 9785 | Berlin (6.1%) | Hague (6.1%) | Paris (8.2%) | .36 |
| B | 146 | 33 | NA | NA | Which bone does the colloquial term "jawbone" refer to? | Mandible | .94 | 9664 | 6869 | Femur (0.0%) | Humerus (4.1%) | Tibia (2.0%) | .51 |
| A | 147 | 140 | NA | NA | Which Aerosmith song was re-made by Run D.M.C.? | Walk this Way | .78 | 8027 | 7003 | Dream On (16.3%) | Dude (2.0%) | Mama Kin (4.1%) | .35 |
| C | 148 | 202 | NA | NA | What is the name for the legendary Egyptian sculpture which asked people a famous riddle? | Sphinx | .63 | 19550 | 11776 | Gate Of The Nile (18.4%) | Mantacor (10.2%) | Ra (8.2%) | .21 |
| D | 150 | 151 | 191 | 211 | What is the capital of Finland? | Helsinki | .76 | 7180 | 6744 | Oslo (10.2%) | Stockholm (6.1%) | Tampere (8.2%) | .35 |
| D | 150 | 254 | 202 | 218 | What is the capital of Canada? | Ottawa | .51 | 6044 | 6713 | Calgary (6.1%) | Toronto (40.8%) | Winnipeg (2.0%) | .10 |
| D | 150 | 193 | 230 | 219 | What is the last name of the inventor of the wireless radio? | Marconi | .65 | 8877 | 6002 | Hertz (20.4%) | Morse (12.2%) | Volta (2.0%) | .24 |
| B | 152 | 50 | NA | NA | What is the largest city and capital of Kenya? | Nairobi | .92 | 7198 | 7649 | Malindi (0.0%) | Mombasa (6.1%) | Nakuru (2.0%) | .51 |
| D | 154 | 19 | 173 | 161 | Who was the most famous Greek doctor? | Hippocrates | .96 | 11936 | 5395 | Democritus (2.0%) | Galen (0.0%) | Parmenides (2.0%) | .57 |
|  |  |  |  |  |  |  |  | MC  (response times) | | MC FOILS  (% of times selected as response) | | |  |
| Set | CR Rank | MC rank | T. et al. rank | N & N rank | Question | CORRECT ANSWER | Correct | Errors | Correct | Foil 1 | Foil 2 | Foil 3 | Difference (MC-CR) |
| D | 154 | 50 | 183 | 193 | What is the last name of the husband-wife spies who were electrocuted in 1951 for passing atomic secrets to Russia? | Rosenberg | .92 | 34325 | 6569 | Hiss (6.1%) | Kramer (2.0%) | Lafayette (0.0%) | .52 |
| B | 155 | 334 | NA | NA | What is the gas that forms dry ice when frozen? | Carbon dioxide | .31 | 8777 | 13646 | Carbon monoxide (2.0%) | Nitrogen (65.3%) | Oxygen (2.0%) | -.08 |
| C | 157 | 93 | NA | NA | What was the last name of the explorer who tramped through what is now Florida looking for the fountain of youth? | Ponce De Leon | .88 | 11952 | 9954 | De La Vega (2.0%) | De Soto (6.1%) | Diego (4.1%) | .49 |
| C | 157 | 165 | NA | NA | What is the last name of the author of 'Call of the Wild'? | London | .73 | 9753 | 6880 | Blake (2.0%) | Bradbury (20.4%) | Heller (4.1%) | .35 |
| D | 159 | 181 | 157 | 156 | What kind of poison did Socrates take as his execution? | Hemlock | .69 | 6445 | 6928 | Arsenic (14.3%) | Belladonna (4.1%) | Nightshade (12.2%) | .32 |
| D | 159 | 235 | 216 | 230 | What is the last name of the woman who founded the American Red Cross? | Barton | .55 | 6759 | 5646 | Dix (2.0%) | Nightingale (38.8%) | Tompkins (4.1%) | .17 |
| C | 161 | 93 | NA | NA | Who was the person who visited the Lilliputians? | Gulliver | .88 | 8660 | 5881 | Bongo (0.0%) | Gargantua (4.1%) | Goliath (8.2%) | .50 |
|  |  |  |  |  |  |  |  | MC  (response times) | | MC FOILS  (% of times selected as response) | | |  |
| Set | CR Rank | MC rank | T. et al. rank | N & N rank | Question | CORRECT ANSWER | Correct | Errors | Correct | Foil 1 | Foil 2 | Foil 3 | Difference (MC-CR) |
| C | 161 | 114 | NA | NA | Who was Robin Hood's greatest enemy? | Sheriff of Nottingham | .82 | 11071 | 7572 | King Arthur (6.1%) | Little John (8.2%) | Prince Valiant (4.1%) | .44 |
| B | 166 | 123 | NA | NA | What is the navigation instrument used at sea to plot positioning by the stars? | Sextant | .82 | 14285 | 7233 | Altimeter (2.0%) | Barometer (4.1%) | Compass (12.2%) | .45 |
| B | 166 | 193 | NA | NA | What do you call a word or sentence that reads the same backward or forward, such as "Madam, I'm Adam"? | Palindrome | .65 | 11045 | 10294 | Anagram (24.5%) | Pangram (8.2%) | Tautonym (2.0%) | .28 |
| B | 166 | 193 | NA | NA | Which body of water separates Central and South America? | Panama Canal | .65 | 14753 | 12244 | Atlantic Ocean (4.1%) | Caribbean Sea (10.2%) | Gulf Of Mexico (20.4%) | .28 |
| B | 166 | 202 | NA | NA | What word means to cut or chop (food) into very small pieces? | Mince | .63 | 9070 | 8980 | Cube (0.0%) | Dice (36.7%) | Julienne (0.0%) | .26 |
| B | 166 | 212 | NA | NA | What was the capital of Czechoslovakia? | Prague | .61 | 8946 | 7457 | Budapest (26.5%) | Czechia (12.2%) | Ostrava (0.0%) | .24 |
| B | 166 | 235 | NA | NA | What is the mixture of two or more metallic elements? | Alloy | .55 | 7149 | 8949 | Alchemy (4.1%) | Amalgam (10.2%) | Compound (30.6%) | .18 |
|  |  |  |  |  |  |  |  |  |  |  |  |  |  |
|  |  |  |  |  |  |  |  | MC  (response times) | | MC FOILS  (% of times selected as response) | | |  |
| Set | CR Rank | MC rank | T. et al. rank | N & N rank | Question | CORRECT ANSWER | Correct | Errors | Correct | Foil 1 | Foil 2 | Foil 3 | Difference (MC-CR) |
| B | 166 | 245 | NA | NA | What do you call the leather band formerly used for sharpening an old-fashioned razor? | Strop | .53 | 8352 | 6829 | Coticule (0.0%) | Strap (44.9%) | Switch (2.0%) | .16 |
| B | 166 | 245 | NA | NA | What is the colloquial term for patella? | Kneecap | .53 | 10823 | 6943 | Elbow (10.2%) | Funny Bone (24.5%) | Shin (12.2%) | .16 |
| B | 166 | 274 | NA | NA | What is the name of the hardwood of an (Asian) Indian timber tree often used for furniture? | Teak | .45 | 12781 | 12452 | Bamboo (20.4%) | Cypress (2.0%) | Mahogany (32.7%) | .08 |
| A | 172 | 181 | NA | NA | Who was Elton John's chief lyricist? | Bernie Taupin | .69 | 14492 | 5748 | Alan Gordon (14.3%) | Jimmy Webb (10.2%) | Robert Hunter (6.1%) | .33 |
| A | 172 | 202 | NA | NA | Which serial killer called himself "Son of Sam"? | David Berkowitz | .63 | 11329 | 5633 | John Wayne Gacy (22.4%) | Samuel Jeffries (6.1%) | Ted Bundy (8.2%) | .26 |
| D | 174 | 343 | 182 | 191 | Who is known as "The Father of Geometry"? | Euclid | .29 | 6793 | 14402 | Archimedes (18.4%) | Ptolemy (8.2%) | Pythagoras (44.9%) | -.08 |
| D | 174 | 225 | 260 | 186 | What was the name of the Union ironclad ship that fought the Confederate ironclad Merrimack? | Monitor | .57 | 9054 | 8582 | Cumberland (24.5%) | Manassas (14.3%) | Virginia (4.1%) | .21 |
| C | 175 | 140 | NA | NA | In 'Romeo and Juliet' who were the Montagues feuding with? | Capulets | .78 | 11661 | 6451 | Goodfellows (10.2%) | Minarets (10.2%) | Rolettes (2.0%) | .42 |
|  |  |  |  |  |  |  |  | MC  (response times) | | MC FOILS  (% of times selected as response) | | |  |
| Set | CR Rank | MC rank | T. et al. rank | N & N rank | Question | CORRECT ANSWER | Correct | Errors | Correct | Foil 1 | Foil 2 | Foil 3 | Difference (MC-CR) |
| B | 178 | 103 | NA | NA | What is the last name of the actor who starred in "Hart to Hart" and "Switch" on TV and also in many movies? | Wagner | .86 | 11330 | 8224 | Conrad (2.0%) | Garner (8.2%) | Reynolds (4.1%) | .51 |
| B | 178 | 175 | NA | NA | What do you call a person who collects and studies stamps? | Philatelist | .71 | 10802 | 8177 | Falerist (0.0%) | Numismatist (8.2%) | Phillumenist (20.4%) | .36 |
| B | 178 | 245 | NA | NA | What is the name of the river that runs through Rome? | Tiber | .53 | 9012 | 7677 | Arno (22.4%) | Elbe (20.4%) | Rubicon (4.1%) | .18 |
| D | 178 | 334 | 265 | 222 | What is the name of the mountain range that separates Asia from Europe? | Ural | .31 | 9802 | 10309 | Alps (32.7%) | Dolomites (4.1%) | Pyrenees (32.7%) | -.05 |
| A | 183 | 103 | NA | NA | What was the 2001 event that led to the bankruptcy of a U.S. energy company and resulted in the dissolution of Arthur Anderson? | The Enron Scandal | .86 | 11380 | 12844 | The Dot-Com Scandal (0.0%) | The Madoff Scandal (8.2%) | The Watergate Scandal (6.1%) | .51 |
| A | 183 | 110 | NA | NA | Whose autobiography is titled "Lady Sings the Blues"? | Billie Holiday | .84 | 5635 | 6322 | Nina Simone (6.1%) | Peggy Lee (2.0%) | Rosemary Clooney (8.2%) | .49 |
| A | 183 | 151 | NA | NA | What is the name of the Viking who discovered Greenland? | Erik the Red | .76 | 11524 | 8837 | Eric Bloodaxe (6.1%) | Freydis Eriksdottir (12.2%) | Ragnar Lodbrok (6.1%) | .40 |
|  |  |  |  |  |  |  |  | MC  (response times) | | MC FOILS  (% of times selected as response) | | |  |
| Set | CR Rank | MC rank | T. et al. rank | N & N rank | Question | CORRECT ANSWER | Correct | Errors | Correct | Foil 1 | Foil 2 | Foil 3 | Difference (MC-CR) |
| A | 183 | 212 | NA | NA | What was Bob Dylan's birth name? | Robert Zimmerman | .61 | 11629 | 9158 | Dylan Roberts (12.2%) | Robert Dillon (16.3%) | Robert Zimman (10.2%) | .26 |
| A | 183 | 245 | NA | NA | Where was the 1939 World's Fair held? | New York City | .53 | 7720 | 9374 | Berlin (8.2%) | London (20.4%) | Paris (18.4%) | .18 |
| A | 183 | 293 | NA | NA | What did the irascible Ted Williams do in the final at bat of his 19-year major-league career? | Hit a home run | .41 | 13651 | 12513 | Get hit by a pitch (6.1%) | Punch the umpire (22.4%) | Strike out (30.6%) | .06 |
| C | 186 | 165 | NA | NA | On top of which mountain was Moses given the Ten Commandments? | Sinai | .73 | 8233 | 7900 | Calgary (8.2%) | Mount of Olives (14.3%) | Mt. Vesuvius (4.1%) | .39 |
| B | 189 | 74 | NA | NA | What is the last name of the Cuban leader that Castro overthrew? | Batista | .90 | 11401 | 7069 | Alfonso (4.1%) | Palma (4.1%) | Torrado (2.0%) | .56 |
| B | 189 | 225 | NA | NA | What is the name of the fountain in Rome into which coins are thrown for good luck? | Trevi | .57 | 9126 | 6439 | Bellagio (32.7%) | Latona (10.2%) | Turtle (0.0%) | .24 |
| B | 189 | 282 | NA | NA | What was the English settlement that vanished in the late 1580s? | Roanoke | .43 | 10104 | 7615 | Jamestown (32.7%) | Plymouth (12.2%) | Yorktown (12.2%) | .10 |
|  |  |  |  |  |  |  |  |  |  |  |  |  |  |
|  |  |  |  |  |  |  |  | MC  (response times) | | MC FOILS  (% of times selected as response) | | |  |
| Set | CR Rank | MC rank | T. et al. rank | N & N rank | Question | CORRECT ANSWER | Correct | Errors | Correct | Foil 1 | Foil 2 | Foil 3 | Difference (MC-CR) |
| B | 189 | 334 | NA | NA | What do you call a moderately fast gait of a horse in which the legs move in diagonal pairs? | Trot | .31 | 11887 | 15862 | Canter (42.9%) | Gallop (24.5%) | Running walk (2.0%) | -.03 |
| C | 192 | 6.5 | NA | NA | What is the term given to pure spiritual love which is devoid of carnal desires between members of opposite sexes? | Platonic | .98 | 17290 | 11672 | Idealistic (0.0%) | Incorporeal (2.0%) | Rational (0.0%) | .65 |
| C | 192 | 74 | NA | NA | What was the disease known as the 'Black Plague'? | Bubonic | .90 | 7701 | 5956 | Cholera (6.1%) | Leprosy (0.0%) | Smallpox (4.1%) | .57 |
| C | 192 | 202 | NA | NA | What was the last name of the person known as the 'desert fox' | Rommel | .63 | 11143 | 11117 | Alexander (2.0%) | Eisenhower (22.4%) | Montgomery (12.2%) | .30 |
| D | 196 | 172 | 199 | 209 | What is the last name of the first person to climb Mount Everest? | Hillary | .71 | 5691 | 6367 | Brown (12.2%) | Desio (6.1%) | Wickersham (10.2%) | .40 |
| D | 196 | 225 | 223 | 237 | What is the last name of the British admiral who won the Battle of Trafalgar? | Nelson | .57 | 8797 | 7477 | Abbott (2.0%) | Drake (28.6%) | Wellesley (12.2%) | .25 |
| D | 196 | 347 | 235 | 210 | What is the last name of the French author who wrote “The Stranger”? | Camus | .29 | 7484 | 6542 | Proust (26.5%) | Sartre (34.7%) | Simenon (10.2%) | -.03 |
|  |  |  |  |  |  |  |  | MC  (response times) | | MC FOILS  (% of times selected as response) | | |  |
| Set | CR Rank | MC rank | T. et al. rank | N & N rank | Question | CORRECT ANSWER | Correct | Errors | Correct | Foil 1 | Foil 2 | Foil 3 | Difference (MC-CR) |
| D | 196 | 123 | 267 | 229 | What is the last name of the man who created the comic strip "Li'l Abner"? | Capp | .82 | 8140 | 6142 | Adams (10.2%) | Caniff (6.1%) | Frank (2.0%) | .50 |
| A | 198 | 304 | NA | NA | To which continent is the yam native? | Africa | .39 | 8587 | 7510 | Australia (10.2%) | Europe (10.2%) | South America (40.8%) | .07 |
| B | 200 | 50 | NA | NA | What word means to raise into position by means of a pulley? | Hoist | .92 | 6858 | 7093 | Erect (2.0%) | Lift (4.1%) | Upheave (2.0%) | .60 |
| B | 200 | 293 | NA | NA | What is the old name of Taiwan? | Formosa | .41 | 7833 | 7540 | Burma (20.4%) | Kampuchea (2.0%) | Siam (36.7%) | .09 |
| C | 201 | 235 | NA | NA | What was the creature killed by Theseus which had the body of a man and the head of a bull? | Minotaur | .55 | 13245 | 12854 | Centaur (38.8%) | Griffon (2.0%) | Hobgoblin (4.1%) | .24 |
| D | 203 | 293 | 153 | 203 | In which city is Michelangelo's statue of David located? | Florence | .41 | 7835 | 7360 | Milan (8.2%) | Rome (34.7%) | Venice (16.3%) | .11 |
| D | 203 | 274 | 169 | 189 | What is the last name of the man who invented dynamite? | Nobel | .45 | 11640 | 6345 | Oppenheimer (22.4%) | Penniman  (14.3%) | Wilbrand (18.4%) | .15 |
| D | 203 | 262 | 259 | 180 | What is John Kenneth Galbraith's profession? | Economist | .49 | 9524 | 9976 | Doctor (6.1%) | Historian (28.6%) | Musician (16.3%) | .19 |
|  |  |  |  |  |  |  |  |  |  |  |  |  |  |
|  |  |  |  |  |  |  |  | MC  (response times) | | MC FOILS  (% of times selected as response) | | |  |
| Set | CR Rank | MC rank | T. et al. rank | N & N rank | Question | CORRECT ANSWER | Correct | Errors | Correct | Foil 1 | Foil 2 | Foil 3 | Difference (MC-CR) |
| C | 205 | 193 | NA | NA | Who was the bouncy and egotistical friend of Christopher Robin? | Tigger | .65 | 12109 | 9606 | Piglet (12.2%) | Rabbit (4.1%) | Roo (18.4%) | .35 |
| A | 207 | 123 | NA | NA | What is a network designed to allow communication within an organization? | An intranet | .82 | 11493 | 10020 | Portal (10.2%) | The internet (4.1%) | Yahoo (4.1%) | .52 |
| A | 207 | 165 | NA | NA | What was invented by Wilhelm Rontgen in 1895? | X-ray machine | .73 | 11881 | 8666 | Diesel engine (16.3%) | Jukebox (4.1%) | Radio (6.1%) | .44 |
| A | 207 | 245 | NA | NA | Who is the national poet of Scotland? | Robert Burns | .53 | 8544 | 6203 | Robert Louis Stevenson (26.5%) | Walter Scott (8.2%) | William Dunbar (12.2%) | .23 |
| B | 210 | 217 | NA | NA | What do you call a sharp and sudden pain in the side? | Stitch | .59 | 8111 | 5999 | Cramp (32.7%) | Hitch (2.0%) | Pull (6.1%) | .30 |
| B | 210 | 293 | NA | NA | What is the metric for gemstone quality? | Carat | .41 | 9876 | 11646 | Clarity (53.1%) | Cut (6.1%) | Ounces (0.0%) | .11 |
| D | 211 | 235 | 196 | 176 | What was the last name of the composer of the "Maple Leaf Rag"? | Joplin | .55 | 7207 | 7063 | Ellington (16.3%) | Gershwin (22.4%) | Parker (6.1%) | .26 |
| C | 212 | 123 | NA | NA | What is the long process by which a dead organism turns to stone? | Petrification | .82 | 10970 | 9337 | Decomposition (2.0%) | Ossification (12.2%) | Rigor Mortis (4.1%) | .53 |
|  |  |  |  |  |  |  |  | MC  (response times) | | MC FOILS  (% of times selected as response) | | |  |
| Set | CR Rank | MC rank | T. et al. rank | N & N rank | Question | CORRECT ANSWER | Correct | Errors | Correct | Foil 1 | Foil 2 | Foil 3 | Difference (MC-CR) |
| A | 214 | 235 | NA | NA | What was the first feature-length motion picture with sound? | The Jazz Singer | .55 | 10543 | 8754 | 42nd Street (12.2%) | Gone With the Wind (22.4%) | Wings (10.2%) | .27 |
| A | 214 | 235 | NA | NA | Which sport has the highest amount of doping? | Cycling | .55 | 8676 | 16835 | Baseball (16.3%) | Football (12.2%) | Wrestling (16.3%) | .27 |
| A | 214 | 316 | NA | NA | What is the only nut tree native to North America? | Pecan | .35 | 9103 | 10374 | Almond (4.1%) | Pistachio (2.0%) | Walnut (59.2%) | .07 |
| B | 218 | 151 | NA | NA | What do you call the purification or purgation of the emotions that is supposed to happen through art? | Catharsis | .76 | 17225 | 9562 | Ablution (14.3%) | Pathos (8.2%) | Repression (2.0%) | .48 |
| B | 218 | 212 | NA | NA | What is the name of the island on which Napoleon was born? | Corsica | .61 | 7945 | 7484 | Cyprus (14.3%) | Malta (18.4%) | Palma (6.1%) | .33 |
| B | 218 | 225 | NA | NA | What is the last name of the man who said, "I only regret that I have but one life to lose for my country"? | Hale | .57 | 10305 | 10313 | Arnold (34.7%) | Greene (4.1%) | Howe (4.1%) | .29 |
| B | 218 | 308 | NA | NA | What is Jane Goodall famous for studying? | Chimpanzees | .37 | 6851 | 5042 | Bears (0.0%) | Gorillas (61.2%) | Turtles (2.0%) | .09 |
| D | 221 | 202 | 161 | 231 | What is the city in which the baseball hall of fame is located? | Cooperstown | .63 | 6401 | 6576 | Canton (18.4%) | Newport (2.0%) | Springfield (16.3%) | .36 |
|  |  |  |  |  |  |  |  | MC  (response times) | | MC FOILS  (% of times selected as response) | | |  |
| Set | CR Rank | MC rank | T. et al. rank | N & N rank | Question | CORRECT ANSWER | Correct | Errors | Correct | Foil 1 | Foil 2 | Foil 3 | Difference (MC-CR) |
| D | 221 | 110 | 215 | 184 | What is the last name of the movie actor who portrayed Spartacus? | Douglas | .84 | 6685 | 7068 | Curtis (6.1%) | Olivier (10.2%) | Strode (0.0%) | .56 |
| A | 222 | 245 | NA | NA | Which famous explorer first introduced iced desserts into Europe? | Marco Polo | .53 | 10725 | 11521 | Christopher Columbus (2.0%) | Ferdinand Magellan (22.4%) | James Cook (22.4%) | .27 |
| B | 223 | 262 | NA | NA | What is the name of the Mediterranean island south of Turkey that is an independent republic and a member of the Commonwealth? | Cyprus | .49 | 11557 | 11164 | Crete (16.3%) | Malta (24.5%) | Sardinia (10.2%) | .23 |
| D | 225 | 50 | 165 | 164 | In addition to the Kentucky Derby and the Belmont Stakes what horse race comprises the Triple Crown? | Preakness | .92 | 9910 | 6666 | Ascot (2.0%) | Haskell (0.0%) | Santa Anita (6.1%) | .66 |
| D | 225 | 74 | 185 | 190 | What is the last name of the author who wrote the James Bond novels? | Fleming | .90 | 6400 | 5577 | Hamilton (8.2%) | Saltzman (2.0%) | Young (0.0%) | .64 |
| C | 226 | 293 | NA | NA | What open-air public theater was home to William Shakespeare's theatrical company? | Globe | .41 | 8940 | 11659 | Avon (51.0%) | Fortune (2.0%) | Haven (6.1%) | .15 |
|  |  |  |  |  |  |  |  | MC  (response times) | | MC FOILS  (% of times selected as response) | | |  |
| Set | CR Rank | MC rank | T. et al. rank | N & N rank | Question | CORRECT ANSWER | Correct | Errors | Correct | Foil 1 | Foil 2 | Foil 3 | Difference (MC-CR) |
| A | 228 | 151 | NA | NA | What is a Japanese dance drama featuring stylized narrative choreographic movements? | Kabuki | .76 | 10532 | 7634 | Bon Odori (8.2%) | Butoh (4.1%) | Kabuki (12.2%) | .51 |
| A | 228 | 254 | NA | NA | Auguste Rodin's "The Thinker" is found in the garden of a museum in which city? | Paris | .51 | 10345 | 12070 | Amsterdam (12.2%) | Madrid (8.2%) | Rome (28.6%) | .26 |
| A | 228 | 293 | NA | NA | What is Tiger Woods' real first name? | Eldrick | .41 | 8014 | 7043 | Herman (8.2%) | Lionel (40.8%) | Timothy (10.2%) | .16 |
| D | 231 | 274 | 197 | 216 | What is the last name of the playwright who wrote "A Streetcar Named Desire"? | Williams | .45 | 8073 | 7355 | Capote (18.4%) | Kazan (6.1%) | Miller (30.6%) | .21 |
| D | 231 | 217 | 258 | 179 | What is the last name of the judge who was known as "The Law West of the Pecos?" | Bean | .59 | 9228 | 8780 | Cooper (6.1%) | Earp (22.4%) | Parker (12.2%) | .35 |
| B | 234 | 74 | NA | NA | What is the word meaning the promises from God to humans? | Covenant | .90 | 12016 | 7112 | Contract (0.0%) | Oath (4.1%) | Pact (6.1%) | .66 |
| B | 234 | 225 | NA | NA | What is the last name of the cosmonaut who was the first person to orbit the earth? | Gagarin | .57 | 11158 | 7161 | Kizim (10.2%) | Tereshkova (26.5%) | Thagard (6.1%) | .33 |
|  |  |  |  |  |  |  |  | MC  (response times) | | MC FOILS  (% of times selected as response) | | |  |
| Set | CR Rank | MC rank | T. et al. rank | N & N rank | Question | CORRECT ANSWER | Correct | Errors | Correct | Foil 1 | Foil 2 | Foil 3 | Difference (MC-CR) |
| B | 234 | 262 | NA | NA | What word denotes the series of units of weight used to measure gold, in which a pound equals twelve ounces? | Troy | .49 | 13203 | 14800 | Gram (46.9%) | Pennyweight (4.1%) | Tola (0.0%) | .25 |
| B | 234 | 268 | NA | NA | What do you call a formal exercise by a team of marchers? | Drill | .82 | 10674 | 8832 | Choreography (6.1%) | Ensemble (0.0%) | Parade (12.2%) | .61 |
| B | 234 | 293 | NA | NA | What do you call stage entertainment consisting of various unrelated acts, such as magicians, acrobats, etc? | Vaudeville | .41 | 13206 | 10750 | Circus (38.8%) | Revue (18.4%) | Spoof (2.0%) | .17 |
| C | 238 | 19 | NA | NA | What was the last name of the man who asked you not to squeeze the Charmin in the toilet paper commercial? | Whipple | .96 | 12437 | 7115 | Douglas (2.0%) | Rogers (2.0%) | Smith (0.0%) | .72 |
| C | 238 | 103 | NA | NA | What was built to confine the Minotaur and now refers to a maze? | Labyrinth | .86 | 12809 | 10315 | Catacomb (6.1%) | Dungeon (6.1%) | Leviathon (2.0%) | .62 |
| A | 240 | 175 | NA | NA | What was Bugsy Siegel's real first name? | Benjamin | .71 | 5830 | 8252 | Anthony (10.2%) | Meyer (8.2%) | Samuel (10.2%) | .49 |
| A | 240 | 293 | NA | NA | Which first lady was the first to live in the White House? | Abigail Adams | .41 | 9127 | 17103 | Dolley Madison (36.7%) | Elizabeth Monroe (8.2%) | Martha Jefferson (14.3%) | .18 |
|  |  |  |  |  |  |  |  | MC  (response times) | | MC FOILS  (% of times selected as response) | | |  |
| Set | CR Rank | MC rank | T. et al. rank | N & N rank | Question | CORRECT ANSWER | Correct | Errors | Correct | Foil 1 | Foil 2 | Foil 3 | Difference (MC-CR) |
| C | 241 | 151 | NA | NA | Who was the mythical Roman goddess of love? | Venus | .76 | 7255 | 12060 | Athena (12.2%) | Diana (12.2%) | Hera (0.0%) | .53 |
| B | 244 | 202 | NA | NA | What word means to voluntarily sacrifice (e.g., cargo) in order to lighten a ship or aircraft's load in time of distress? | Jettison | .63 | 17610 | 8303 | Abandon (14.3%) | Drop (12.2%) | Scrap (10.2%) | .41 |
| B | 244 | 212 | NA | NA | What is the last name of the actor who played the scarecrow in the movie, "The Wizard of Oz"? | Bolger | .61 | 8905 | 9921 | Ebsen (10.2%) | Haley (16.3%) | Lahr (12.2%) | .39 |
| B | 244 | 274 | NA | NA | What do you call the position of the arms when the hands are on the hips and the elbows are out? | Akimbo | .45 | 13505 | 10518 | Attention (6.1%) | Supine (14.3%) | Teapot (34.7%) | .23 |
| B | 244 | 327 | NA | NA | What is the name of the substance derived from a whale that is used to make perfume? | Ambergris | .33 | 10940 | 11033 | Blubber (38.8%) | Oil (20.4%) | Spermaceti (8.2%) | .10 |
| D | 247 | 304 | 178 | 236 | What is the longest river In Asia? | Yangtze | .39 | 6075 | 8923 | Ganges (36.7%) | Mekong (12.2%) | Yellow (12.2%) | .18 |
| D | 247 | 245 | 244 | 220 | What was the last name of the female star of the movie "Casablanca"? | Bergman | .53 | 8685 | 8249 | Bogart (0.0%) | Hepburn (42.9%) | Rossellini (4.1%) | .32 |
|  |  |  |  |  |  |  |  | MC  (response times) | | MC FOILS  (% of times selected as response) | | |  |
| Set | CR Rank | MC rank | T. et al. rank | N & N rank | Question | CORRECT ANSWER | Correct | Errors | Correct | Foil 1 | Foil 2 | Foil 3 | Difference (MC-CR) |
| D | 247 | 165 | 255 | 163 | What was the last name of the Captain of the British ship "Bounty" when the mutiny occurred? | Bligh | .73 | 10240 | 9622 | Christian (8.2%) | Cook (10.2%) | Laughton (8.2%) | .52 |
| A | 250 | 293 | NA | NA | Halloween traces its origins to which pagan festival? | Samhain | .41 | 9162 | 5596 | Imbolc (8.2%) | Lughnassadh (24.5%) | Ostara (26.5%) | .20 |
| A | 250 | 293 | NA | NA | Who wrote "The Rime of the Ancient Mariner"? | Samuel Taylor Coleridge | .41 | 7158 | 8155 | Hannah More (2.0%) | Percy Bysshe Shelley (24.5%) | William Blake (32.7%) | .20 |
| C | 251 | 50 | NA | NA | What was Fonzie's first name in television's 'Happy Days'? | Arthur | .92 | 14983 | 7536 | Allen (4.1%) | Charlie (2.0%) | Ralph (2.0%) | .71 |
| B | 252 | 123 | NA | NA | What do you call a chain of rocks or coral at or near the surface of the water in an ocean? | Reef | .92 | 14427 | 8545 | Bank (2.0%) | Fjord (0.0%) | Shoal (6.1%) | .27 |
| D | 254 | 6.5 | 257 | 173 | What is the name of Germany's largest battleship that was sunk in World War II? | Bismarck | .98 | 10950 | 7501 | Gneisenau (0.0%) | Kronprinz (0.0%) | Tirpitz  (2.0%) | .78 |
| D | 254 | 378 | 269 | 239 | What is the last name of the first man to run the mile in under four minutes? | Bannister | .20 | 7472 | 7718 | Brasher (8.2%) | Owens (63.3%) | Santee (8.2%) | .01 |
|  |  |  |  |  |  |  |  | MC  (response times) | | MC FOILS  (% of times selected as response) | | |  |
| Set | CR Rank | MC rank | T. et al. rank | N & N rank | Question | CORRECT ANSWER | Correct | Errors | Correct | Foil 1 | Foil 2 | Foil 3 | Difference (MC-CR) |
| A | 257 | 74 | NA | NA | What album holds the world record for copies sold? | Thriller | .90 | 9697 | 9046 | Back in Black (2.0%) | Dark Side of the Moon (6.1%) | Saturday Night Fever Soundtrack (2.0%) | .70 |
| A | 257 | 151 | NA | NA | What food is often used as a substitute for ice cream in advertising photo shoots? | Mashed Potato | .76 | 11702 | 11915 | Cottage Cheese (4.1%) | Milk (2.0%) | Tapioca (18.4%) | .56 |
| A | 257 | 282 | NA | NA | What was Sinatra's chart-topping duet with his daughter Nancy? | Somethin' Stupid | .43 | 9794 | 8959 | Jackson (2.0%) | These Boots Are Made for Walkin' (16.3%) | Unforgettable (38.8%) | .24 |
| A | 257 | 304 | NA | NA | What philosopher was considered the ugliest man in Athens? | Socrates | .39 | 9643 | 7983 | Democritus (24.5%) | Empedocles (22.4%) | Plato (14.3%) | .19 |
| B | 259 | 181 | NA | NA | What word means relating to or situated on the back, especially of an animal? | Dorsal | .69 | 12653 | 8302 | Abaxial (2.0%) | Anterior (22.4%) | Ventral (6.1%) | .51 |
| D | 260 | 262 | 270 | 240 | What is the last name of the author who wrote "The Brothers Karamazov"? | Dostoyevsky | .49 | 9528 | 7064 | Kafka (10.2%) | Solzhenitsyn (10.2%) | Tolstoy (30.6%) | .31 |
| C | 261 | 93 | NA | NA | What is the name of the obnoxious rooster in Warner Brothers cartoons? | Foghorn Leghorn | .88 | 20560 | 8476 | Marvin (4.1%) | Sam (2.0%) | Sylvester (6.1%) | .70 |
| A | 263 | 235 | NA | NA | Who is the lead singer of Limp Bizkit? | Fred Durst | .55 | 9871 | 6897 | Lil Dicky (14.3%) | Lil' Kim (18.4%) | Slim Shady (12.2%) | .38 |
|  |  |  |  |  |  |  |  | MC  (response times) | | MC FOILS  (% of times selected as response) | | |  |
| Set | CR Rank | MC rank | T. et al. rank | N & N rank | Question | CORRECT ANSWER | Correct | Errors | Correct | Foil 1 | Foil 2 | Foil 3 | Difference (MC-CR) |
| A | 263 | 362 | NA | NA | In 1984 Pres. Ronald Reagan won every state except one. Which one did he lose? | Minnesota | .24 | 8963 | 16853 | Massachusetts (22.4%) | New York (28.6%) | Vermont (24.5%) | .07 |
| B | 267 | 225 | NA | NA | What is the name of the man who removed the thorn from the lion's paw in the story from Aesop's fables? | Androcles | .57 | 9998 | 13875 | Cleisthenes (4.1%) | Pericles (34.7%) | Thucydides (4.1%) | .40 |
| B | 267 | 268 | NA | NA | What is the last name of 1980 Olympic hopeful Randy Gardener's ice skating partner? | Babilonia | .47 | 13263 | 8583 | Gordeeva (10.2%) | Harding (28.6%) | Kwan (14.3%) | .30 |
| B | 267 | 316 | NA | NA | What is the name of the formerly independent country north of Lithuania and Latvia that was absorbed into USSR? | Estonia | .35 | 16329 | 15289 | Belarus (12.2%) | Serbia (20.4%) | Slovakia (32.7%) | .18 |
| B | 267 | 327 | NA | NA | Who is the messenger of the gods in Greek mythology? | Hermes | .33 | 9536 | 7984 | Ares (10.2%) | Eos (6.1%) | Mercury (51.0%) | .16 |
| D | 267 | 217 | 170 | 228 | What is the name of the Chinese religion founded by Lao Tse? | Taoism | .59 | 11823 | 8798 | Confucianism (34.7%) | Shamanism (0.0%) | Zen (6.1%) | .43 |
|  |  |  |  |  |  |  |  |  |  |  |  |  |  |
|  |  |  |  |  |  |  |  | MC  (response times) | | MC FOILS  (% of times selected as response) | | |  |
| Set | CR Rank | MC rank | T. et al. rank | N & N rank | Question | CORRECT ANSWER | Correct | Errors | Correct | Foil 1 | Foil 2 | Foil 3 | Difference (MC-CR) |
| D | 267 | 186 | 195 | 171 | What was the name of Alexander Graham Bell's assistant? | Watson | .67 | 10048 | 8904 | Gray (28.6%) | Meucci (2.0%) | Reis (2.0%) | .51 |
| A | 272 | 202 | NA | NA | What is a poem in which the first letters of each line spell a word? | An acrostic | .63 | 12726 | 13521 | A haiku (14.3%) | An ode (12.2%) | A sestina (10.2%) | .47 |
| A | 272 | 202 | NA | NA | What story is the first to feature Edgar Allan Poe's amateur detective C. Auguste Dupin? | Murders in the Rue Morgue | .63 | 14216 | 10844 | A Study in Scarlet (2.0%) | The Mystery of Marie Rogêt (10.2%) | The Purloined Letter (24.5%) | .47 |
| A | 272 | 308 | NA | NA | Niels Bohr used quantum mechanics to describe which element? | Hydrogen | .37 | 13636 | 11272 | Carbon (26.5%) | Helium (20.4%) | Zinc (16.3%) | .21 |
| A | 272 | 362 | NA | NA | What is the alternative name for the Sea of Cortez? | Gulf of California | .24 | 11023 | 9990 | Caribbean Sea (14.3%) | Gulf of Mexico (38.8%) | Gulf of Panama (22.4%) | .09 |
| A | 272 | 412 | NA | NA | Ailurophobia is the fear of what? | Cats | .08 | 10218 | 17200 | Accidents (6.1%) | Clowns (42.9%) | Illness (42.9%) | -.08 |
| D | 276 | 372 | 184 | 261 | What is the last name of the composer who wrote the opera "Don Giovanni?" | Mozart | .22 | 6887 | 9343 | Puccini (30.6%) | Verdi (20.4%) | Wagner (26.5%) | .07 |
|  |  |  |  |  |  |  |  |  |  |  |  |  |  |
|  |  |  |  |  |  |  |  | MC  (response times) | | MC FOILS  (% of times selected as response) | | |  |
| Set | CR Rank | MC rank | T. et al. rank | N & N rank | Question | CORRECT ANSWER | Correct | Errors | Correct | Foil 1 | Foil 2 | Foil 3 | Difference (MC-CR) |
| D | 276 | 165 | 274 | 253 | What is the last name of the author of "Our Town"? | Wilder | .73 | 7535 | 7127 | Akins (2.0%) | Berry (10.2%) | Williams (14.3%) | .58 |
| B | 278 | 254 | NA | NA | What word means to observe and offer unwanted advice or comment, especially at a card game? | Kibitz | .51 | 14021 | 12812 | Chatter (26.5%) | Chutzpah (14.3%) | Shmooze (8.2%) | .36 |
| B | 278 | 327 | NA | NA | What is the last name of the author of Jonathan Livingston Seagull? | Bach | .33 | 10164 | 11418 | Dahl (6.1%) | Hemingway (22.4%) | Segal (38.8%) | .18 |
| A | 281 | 165 | NA | NA | Who invented flexible photographic film? | George Eastman | .73 | 6841 | 7037 | David Houston (2.0%) | Louis Daguerre (12.2%) | Thomas Edison (12.2%) | .59 |
| A | 281 | 274 | NA | NA | Who said "You'd be surprised how much it costs to look this cheap?" | Dolly Parton | .45 | 9971 | 9176 | Joan Rivers (53.1%) | Jessica Simpson (0.0%) | Kim Kardashian (2.0%) | .31 |
| A | 281 | 334 | NA | NA | Which Egyptian god was often represented as a falcon? | Horus | .31 | 8362 | 8780 | Osiris (53.1%) | Seth (6.1%) | Thoth (10.2%) | .17 |
| A | 281 | 372 | NA | NA | What is U2 singer Bono's real name? | Paul Hewson | .22 | 9316 | 10134 | Dave Evans (6.1%) | Jon Bono (42.9%) | Robert Norwood (28.6%) | .08 |
|  |  |  |  |  |  |  |  | MC  (response times) | | MC FOILS  (% of times selected as response) | | |  |
| Set | CR Rank | MC rank | T. et al. rank | N & N rank | Question | CORRECT ANSWER | Correct | Errors | Correct | Foil 1 | Foil 2 | Foil 3 | Difference (MC-CR) |
| D | 284 | 268 | 174 | 165 | In which city does the Cotton Bowl take place? | Dallas | .47 | 9615 | 12769 | Austin (6.1%) | Houston (12.2%) | New Orleans (34.7%) | .33 |
| D | 284 | 406 | 207 | 284 | What is the capital of Australia? | Canberra | .10 | 7013 | 8686 | Brisbane (2.0%) | Melbourne (34.7%) | Sydney (53.1%) | -.03 |
| B | 286 | 193 | NA | NA | What do you call a secret agreement or pact (as between lovers)? | Tryst | .65 | 10721 | 9939 | Date (0.0%) | Rendezvous (24.5%) | Tontine (10.2%) | .52 |
| B | 286 | 274 | NA | NA | What was the name of the capital of the Aztec empire, which was located where Mexico City is today? | Tenochtitlan | .45 | 11246 | 11383 | Mixtec (12.2%) | Teotitlan (20.4%) | Tlaxcala (22.4%) | .32 |
| B | 286 | 293 | NA | NA | What do you call a law that gives exclusive right of inheritance to the eldest son? | Primogeniture | .41 | 11283 | 13325 | Bequest (16.3%) | Estate (10.2%) | Patrimony (32.7%) | .28 |
| A | 288 | 362 | NA | NA | Which lawyer and presidential adviser in 1969 became the only Supreme Court justice to resign under threat of impeachment? | Abe Fortas | .24 | 14985 | 17334 | Arthur Goldberg (18.4%) | William Brennan (16.3%) | William O. Douglas (40.8%) | .12 |
| D | 289 | 245 | 236 | 250 | Who was the first ruler of the Holy Roman Empire? | Charlemagne | .53 | 9112 | 8047 | Alexander (30.6%) | Clovis (8.2%) | Louis (8.2%) | .41 |
|  |  |  |  |  |  |  |  |  |  |  |  |  |  |
|  |  |  |  |  |  |  |  | MC  (response times) | | MC FOILS  (% of times selected as response) | | |  |
| Set | CR Rank | MC rank | T. et al. rank | N & N rank | Question | CORRECT ANSWER | Correct | Errors | Correct | Foil 1 | Foil 2 | Foil 3 | Difference (MC-CR) |
| B | 290 | 343 | NA | NA | What is the national park in Utah, northeast of Zion, which is famous for the rock formations in its canyon? | Bryce | .29 | 18491 | 13930 | Arches (16.3%) | Grand Canyon (38.8%) | Zion (16.3%) | .17 |
| D | 292 | 151 | 166 | 204 | What is the last name of the criminal who was killed by FBI agents outside of a Chicago movie theater? | Dillinger | .76 | 11746 | 8704 | Barker (2.0%) | Floyd (20.4%) | Karis (2.0%) | .65 |
| D | 292 | 165 | 273 | 252 | What is the name of the instrument used to measure wind speed? | Anemometer | .73 | 11606 | 8212 | Actinometer (8.2%) | Ceilometer (12.2%) | Pyranometer (6.1%) | .63 |
| D | 292 | 254 | 275 | 256 | What is the last name of the singer who made a hit recording of the song "Who's Sorry Now?" | Francis | .51 | 11913 | 7129 | Darrin (16.3%) | Day (20.4%) | Sedaka (12.2%) | .40 |
| A | 299 | 245 | NA | NA | Who designed Central Park in New York City? | Frederick Law Olmstead | .53 | 7765 | 11009 | Andre Le Notre (18.4%) | Beatrix Farrand (8.2%) | Jens Jensen (20.4%) | .43 |
| A | 299 | 262 | NA | NA | The Mayan and Aztec peoples used cocoa beans not only to make a delicious beverage but also as...? | Currency | .49 | 11982 | 9154 | Dye (16.3%) | Fertilizer (6.1%) | Medicine (28.6%) | .38 |
|  |  |  |  |  |  |  |  |  |  |  |  |  |  |
|  |  |  |  |  |  |  |  | MC  (response times) | | MC FOILS  (% of times selected as response) | | |  |
| Set | CR Rank | MC rank | T. et al. rank | N & N rank | Question | CORRECT ANSWER | Correct | Errors | Correct | Foil 1 | Foil 2 | Foil 3 | Difference (MC-CR) |
| A | 299 | 262 | NA | NA | Which Bronte sister died of tuberculosis in 1848 and was famously buried in a coffin that was a meager 16 inches (41 cm) wide? | Emily | .49 | 24965 | 11706 | Anne (22.4%) | Charlotte (24.5%) | Elaine (4.1%) | .38 |
| A | 299 | 282 | NA | NA | What breed of dog is known for making a yodeling noise instead of barking? | Basenji | .43 | 9308 | 8002 | Mexican hairless (6.1%) | New-Guinea Singing Dog (49.0%) | Vizsla (2.0%) | .32 |
| A | 299 | 293 | NA | NA | What was author George Eliot's real name? | Mary Ann Evans | .41 | 13447 | 11812 | Elizabeth MacKintosh (28.6%) | Jessie Margaret King (14.3%) | Mary Torrans Lathrap (16.3%) | .30 |
| A | 299 | 316 | NA | NA | In what geological period did birds evolve? | Jurassic | .35 | 8698 | 8679 | Permian (12.2%) | Pleistocene (34.7%) | Triassic (18.4%) | .24 |
| A | 299 | 316 | NA | NA | Polo consists of 8 periods called what? | Chukkers | .35 | 10480 | 10171 | Ends (16.3%) | Octos (20.4%) | Sets (28.6%) | .24 |
| A | 299 | 316 | NA | NA | With which band was David Johansen the lead singer? | The New York Dolls | .35 | 7838 | 6529 | T. Rex (14.3%) | The Clash (44.9%) | The Stooges (6.1%) | .24 |
| A | 299 | 362 | NA | NA | What does a philomath have a love for? | Learning | .24 | 10238 | 11670 | Math (32.7%) | Philanthropy (12.2%) | Philosophy (30.6%) | .14 |
|  |  |  |  |  |  |  |  | MC  (response times) | | MC FOILS  (% of times selected as response) | | |  |
| Set | CR Rank | MC rank | T. et al. rank | N & N rank | Question | CORRECT ANSWER | Correct | Errors | Correct | Foil 1 | Foil 2 | Foil 3 | Difference (MC-CR) |
| A | 299 | 372 | NA | NA | What was the title of Dr. Seuss' first book? | And to Think That I Saw It on Mulberry Street | .22 | 8561 | 9840 | Green Eggs and Ham (22.4%) | Horton Hears a Who! (14.3%) | The Cat in the Hat (40.8%) | .12 |
| A | 299 | 378 | NA | NA | What was the name of George of the Jungle's pet elephant? | Shep | .20 | 10134 | 26481 | Elmer (24.5%) | Jumbo (28.6%) | Tookie (26.5%) | .10 |
| B | 306 | 262 | NA | NA | What word means the form of something in the form of a spiral or coil? | Helix | .49 | 11570 | 11152 | Funnel (8.2%) | Sphere (6.1%) | Spring (36.7%) | .40 |
| B | 306 | 304 | NA | NA | What do you call a verb that does not take a direct object? | Intransitive | .39 | 11233 | 10707 | Active (0.0%) | Passive (42.9%) | Transitive (18.4%) | .30 |
| B | 306 | 412 | NA | NA | What is the last name of the candidate who ran as an independent against Reagan and Carter in 1980? | Anderson | .08 | 10653 | 6005 | Nader (20.4%) | Perot (63.3%) | Wallace (8.2%) | -.01 |
| D | 309 | 316 | 242 | 232 | What is the name of the brightest star in the sky excluding the sun? | Sirius | .35 | 8347 | 11465 | Polaris (44.9%) | Rigel (6.1%) | Vega (14.3%) | .26 |
| D | 309 | 225 | 261 | 197 | What is the last name of the doctor who performed the first successful human heart transplant? | Barnard | .57 | 7170 | 10090 | Blackwell (12.2%) | Darnell (8.2%) | Mayo (22.4%) | .48 |
|  |  |  |  |  |  |  |  | MC  (response times) | | MC FOILS  (% of times selected as response) | | |  |
| Set | CR Rank | MC rank | T. et al. rank | N & N rank | Question | CORRECT ANSWER | Correct | Errors | Correct | Foil 1 | Foil 2 | Foil 3 | Difference (MC-CR) |
| C | 310 | 193 | NA | NA | What mythical creature did Perseus kill? | Medusa | .65 | 14375 | 10412 | Andromeda (8.2%) | Hydra (20.4%) | Kraken (6.1%) | .56 |
| A | 313 | 225 | NA | NA | Who was the greatest poet of Russia's golden age? | Alexander Pushkin | .57 | 7692 | 7443 | Osip Mandelstam (10.2%) | Sergei Yesenin (16.3%) | Yevgeny Yevtushenko (16.3%) | .48 |
| A | 313 | 282 | NA | NA | Who wrote the "Threepenny Opera"? | Bertolt Brecht | .43 | 7342 | 7482 | Carl Zuckmayer (20.4%) | Ernst Toller (28.6%) | Frank Wedekind (8.2%) | .34 |
| A | 313 | 293 | NA | NA | What was the first product to have a barcode? | Wrigley gum | .41 | 7809 | 7611 | Coca-cola (34.7%) | Milky Way (4.1%) | Wonder Bread (20.4%) | .32 |
| A | 313 | 316 | NA | NA | In which Cleveland suburb did actor Paul Newman grow up? | Shaker Heights | .35 | 6476 | 8159 | Beachwood (16.3%) | Cleveland Heights (24.5%) | Lakewood (24.5%) | .26 |
| A | 313 | 412 | NA | NA | The term "op-ed" is a shortened form of which phrase? | Opposite the editorial page | .08 | 11239 | 21467 | Opinion editorial (87.8%) | Optional editorial (4.1%) | Open engagement (0.0%) | -.01 |
| D | 317 | 225 | 286 | 277 | What was the last name of Billy the Kid? | Bonney | .57 | 8992 | 7962 | Brady (6.1%) | Garrett (26.5%) | James (10.2%) | .50 |
| D | 317 | 384 | 288 | 279 | From what musical is the song “Baubles Bangles And Beads”? | Kismet | .18 | 9801 | 17535 | Carousel (34.7%) | Gigi (22.4%) | Kiss Me, Kate (24.5%) | .11 |
| B | 318 | 372 | NA | NA | What word means lasting only a very brief time? | Ephemeral | .22 | 8493 | 7941 | Evanescent (12.2%) | Timeless (2.0%) | Transient (63.3%) | .15 |
|  |  |  |  |  |  |  |  |  |  |  |  |  |  |
|  |  |  |  |  |  |  |  | MC  (response times) | | MC FOILS  (% of times selected as response) | | |  |
| Set | CR Rank | MC rank | T. et al. rank | N & N rank | Question | CORRECT ANSWER | Correct | Errors | Correct | Foil 1 | Foil 2 | Foil 3 | Difference (MC-CR) |
| A | 324 | 165 | NA | NA | What is the name of Ulysses Everett McGill's hair pomade of choice in "O Brother Where Art Thou"? | Dapper Dan | .73 | 16028 | 10382 | Handsome Hank (2.0%) | Natty Ned (10.2%) | Stylish Stu (14.3%) | .66 |
| A | 324 | 212 | NA | NA | What philosopher was imprisoned by the British government in 1918 for campaigning against war and conscription? | Bertrand Russell | .61 | 17669 | 9596 | C.D. Broad (4.1%) | Frank Ramsey (20.4%) | Gilbert Ryle (14.3%) | .54 |
| A | 324 | 225 | NA | NA | The theme tune for 'Monty Python's Flying Circus' was written by which composer? | John Philip Sousa | .57 | 10442 | 9038 | Albert Austin Harding (10.2%) | Charles Benter (20.4%) | Edwin Franko Goldman (12.2%) | .50 |
| A | 324 | 274 | NA | NA | What is a chord consisting of three tones of a diatonic scale called? | Triad | .45 | 11831 | 13090 | Diminished fifth (24.5%) | Treble (4.1%) | Seventh (26.5%) | .38 |
| A | 324 | 304 | NA | NA | What was the name of the U.S. surveillance ship that was attacked by North Vietnam in the Gulf of Tonkin? | USS Maddox | .39 | 11006 | 13603 | HMAS Brisbane (10.2%) | USS Canberra (8.2%) | USS Wichita (42.9%) | .32 |
| A | 324 | 343 | NA | NA | What do A. E. Housman's initials stand for? | Alfred Edward | .29 | 10528 | 11102 | Albert Everett (24.5%) | Alfred Edgar (36.7%) | Anthony Eric (10.2%) | .22 |
|  |  |  |  |  |  |  |  | MC  (response times) | | MC FOILS  (% of times selected as response) | | |  |
| Set | CR Rank | MC rank | T. et al. rank | N & N rank | Question | CORRECT ANSWER | Correct | Errors | Correct | Foil 1 | Foil 2 | Foil 3 | Difference (MC-CR) |
| A | 324 | 362 | NA | NA | In which film did Doris Day sing the Oscar winning song "Secret Love"? | Calamity Jane | .24 | 14140 | 15345 | By the Light of the Silvery Moon (14.3%) | I'll See You in My Dreams (28.6%) | Tea for Two (32.7%) | .17 |
| A | 324 | 362 | NA | NA | Which first lady initiated the Easter egg roll on the White House lawn? | Lucy Hayes | .24 | 9901 | 14305 | Helen Taft (61.2%) | Lou Hoover (10.2%) | Sarah Polk (4.1%) | .17 |
| A | 324 | 378 | NA | NA | Who is the author of "Ozymandias"? | Percy Bysshe Shelley | .20 | 7770 | 6480 | John Keats (24.5%) | William Blake (26.5%) | William Wordsworth (28.6%) | .13 |
| A | 324 | 390 | NA | NA | What is LL Cool J.'s real name? | James Todd Smith | .16 | 9717 | 17030 | Andre Romelle Young (12.2%) | Curtis James Jackson III (61.2%) | Earl Simmons (10.2%) | .09 |
| A | 324 | 416 | NA | NA | Aldebaran is the brightest star in which constellation? | Taurus | .06 | 8298 | 4457 | Orion (44.9%) | Scorpius (10.2%) | Ursa Major (38.8%) | -.01 |
| B | 330 | 316 | NA | NA | What do you call a solemn declaration made by a person who conscientiously declines taking an oath? | Affirmation | .35 | 18486 | 15057 | Averment (46.9%) | Declaration (10.2%) | Testimonial (8.2%) | .29 |
| A | 331 | 175 | NA | NA | Who assassinated Mohandas Gandhi? | A Hindu nationalist | .71 | 17176 | 11145 | A bodyguard (18.4%) | A British officer (4.1%) | A spy (6.1%) | .66 |
| A | 339 | 123 | NA | NA | What were the first tennis racket strings made of? | Sheep gut | .82 | 7530 | 6131 | Aluminum (6.1%) | Horse hair (8.2%) | Plastic (4.1%) | .76 |
|  |  |  |  |  |  |  |  |  |  |  |  |  |  |
|  |  |  |  |  |  |  |  | MC  (response times) | | MC FOILS  (% of times selected as response) | | |  |
| Set | CR Rank | MC rank | T. et al. rank | N & N rank | Question | CORRECT ANSWER | Correct | Errors | Correct | Foil 1 | Foil 2 | Foil 3 | Difference (MC-CR) |
| A | 339 | 202 | NA | NA | Myosotis is the latin name for which type of flower? | Forget-Me-Not | .63 | 9926 | 9863 | Borage (16.3%) | Bugloss (6.1%) | Comfrey (14.3%) | .58 |
| A | 339 | 254 | NA | NA | What is the world governing body of tennis? | International Tennis Federation | .51 | 25394 | 10725 | Association of Tennis Professionals (4.1%) | Federation Internationale de Tennis (20.4%) | World Tennis League (24.5%) | .46 |
| A | 339 | 282 | NA | NA | Who was the lead vocalist for 'The Dakotas' during the 1960s?" | Billy J. Kramer | .43 | 10463 | 8878 | Alma Cogan (12.2%) | Billy Fury (32.7%) | John Lennon (12.2%) | .38 |
| A | 339 | 304 | NA | NA | Where were the 1952 Olympics held? | Helsinki | .39 | 7689 | 6991 | London (22.4%) | Stockholm (24.5%) | Tokyo (14.3%) | .34 |
| A | 339 | 316 | NA | NA | The 1912 Summer Olympics were held in what city? | Stockholm | .35 | 8917 | 8177 | Copenhagen (18.4%) | Helsinki (16.3%) | Oslo (30.6%) | .29 |
| A | 339 | 327 | NA | NA | W. Somerset Maugham's 1919 novel "The Moon and Sixpence" is based on the life of what artist? | Paul Gauguin | .33 | 9876 | 16369 | Claude Monet (34.7%) | Juan Gris (4.1%) | Paul Cezanne (28.6%) | .27 |
| A | 339 | 334 | NA | NA | In which city is the Encyclopedia Britannica published? | Chicago | .31 | 11281 | 5449 | Boston (24.5%) | London (38.8%) | New York (6.1%) | .25 |
| A | 339 | 334 | NA | NA | Who discovered the law of electrolysis? | Michael Faraday | .31 | 9983 | 9219 | Alessandro Volta (34.7%) | James Watt (12.2%) | Samuel Morse (22.4%) | .25 |
|  |  |  |  |  |  |  |  |  |  |  |  |  |  |
|  |  |  |  |  |  |  |  | MC  (response times) | | MC FOILS  (% of times selected as response) | | |  |
| Set | CR Rank | MC rank | T. et al. rank | N & N rank | Question | CORRECT ANSWER | Correct | Errors | Correct | Foil 1 | Foil 2 | Foil 3 | Difference (MC-CR) |
| A | 339 | 362 | NA | NA | Which French author's novel "Germinal" depicts life in a mining community? | Emile Zola | .24 | 12232 | 14716 | Alexandre Dumas (30.6%) | Gustave Flaubert (28.6%) | Victor Hugo (16.3%) | .19 |
| A | 339 | 378 | NA | NA | What was the most played song on the radio in the United States during the 20th century? | You've Lost That Lovin' Feelin' | .20 | 13509 | 29352 | Every Breath You Take (4.1%) | Sitting on the Dock of the Bay (14.3%) | Yesterday (61.2%) | .15 |
| A | 339 | 384 | NA | NA | What is Australia's highest mountain? | Mount Kosciuszko | .18 | 7089 | 5535 | Mount Tate (28.6%) | Mount Townsend (28.6%) | Mount Twynam (24.5%) | .13 |
| A | 339 | 406 | NA | NA | Tony Drago is a snooker professional from which country? | Malta | .10 | 9777 | 19412 | England (16.3%) | Ireland (36.7%) | Italy (36.7%) | .05 |
| A | 339 | 406 | NA | NA | Who was the first person to admit to practicing witchcraft in Salem? | Tituba | .10 | 10950 | 13396 | Mary Bradbury (26.5%) | Rebecca Nurse (14.3%) | Sarah Good (49.0%) | .05 |
| D | 347 | 193 | 208 | 259 | What is the last name of the artist who painted "The Persistence of Memory"? | Dali | .65 | 8339 | 7432 | de Chirico (12.2%) | Duchamp (6.1%) | Miro (16.3%) | .61 |
| D | 347 | 396 | 279 | 266 | What is the last name of the author of "The Agony and the Ecstasy"? | Stone | .14 | 8367 | 6462 | Maugham (26.5%) | Reed (20.4%) | Shaw (38.8%) | .10 |
|  |  |  |  |  |  |  |  |  |  |  |  |  |  |
|  |  |  |  |  |  |  |  | MC  (response times) | | MC FOILS  (% of times selected as response) | | |  |
| Set | CR Rank | MC rank | T. et al. rank | N & N rank | Question | CORRECT ANSWER | Correct | Errors | Correct | Foil 1 | Foil 2 | Foil 3 | Difference (MC-CR) |
| B | 348 | 416 | NA | NA | What word means to deposit (something valuable) as a security for money borrowed? | Pawn | .06 | 8822 | 11496 | Collateral (91.8%) | Mortgage (2.0%) | Pledge (0.0%) | .02 |
| A | 359 | 193 | NA | NA | Jockey Lester Piggott served 3 years in prison for what crime? | Tax evasion | .65 | 10640 | 11489 | Fraud (8.2%) | Identity theft (6.1%) | Insider trading (20.4%) | .62 |
| A | 359 | 202 | NA | NA | What is the capital of Guinea-Bissau? | Bissau | .63 | 5500 | 7654 | Antula (18.4%) | Hafia (2.0%) | Madina (16.3%) | .60 |
| A | 359 | 212 | NA | NA | Who patented a burglar proof lock described as "magic and infallible"? | Linus Yale | .61 | 8932 | 8251 | Elias Howe, Jr. (14.3%) | Henry R. Towne (16.3%) | James Lovelock (8.2%) | .58 |
| A | 359 | 225 | NA | NA | What was the first graphical browser for the World Wide Web? | Mosaic | .57 | 13303 | 10448 | Basic (24.5%) | Firefox (10.2%) | Google (8.2%) | .54 |
| A | 359 | 282 | NA | NA | What is Spike Milligan's real first name? | Terence | .43 | 7367 | 6011 | Alan (4.1%) | Patrick (42.9%) | Robert (10.2%) | .39 |
| A | 359 | 316 | NA | NA | Who was the famous actor who played the Swedish Chef's uncle in an episode of "The Muppet Show"? | Danny Kaye | .35 | 12247 | 15972 | Don Knotts (12.2%) | Steve Martin (38.8%) | Sylvester Stallone (14.3%) | .31 |
|  |  |  |  |  |  |  |  | MC  (response times) | | MC FOILS  (% of times selected as response) | | |  |
| Set | CR Rank | MC rank | T. et al. rank | N & N rank | Question | CORRECT ANSWER | Correct | Errors | Correct | Foil 1 | Foil 2 | Foil 3 | Difference (MC-CR) |
| A | 359 | 334 | NA | NA | Who became Archbishop of Canterbury in 1980? | Robert Runcie | .31 | 8262 | 10356 | Frederick Coggan (30.6%) | George Carey (16.3%) | Rowan Williams (22.4%) | .27 |
| A | 359 | 343 | NA | NA | What opera by Giacomo Puccini was left unfinished at his death? | Turandot | .29 | 8935 | 13336 | La Boheme (30.6%) | Madama Butterfly (22.4%) | The Girl of the Golden West (18.4%) | .25 |
| A | 359 | 343 | NA | NA | Which Cleveland mayor was the first African American mayor of a major U.S. city? | Carl Stokes | .29 | 10112 | 9391 | Harold Washington (42.9%) | Maynard Jackson (28.6%) | Sly James (0.0%) | .25 |
| A | 359 | 351 | NA | NA | What is the largest known butterfly? | Queen Alexandra's Birdwing | .27 | 9561 | 8727 | Goliath Birdwing (40.8%) | Homerus Swallowtail (32.7%) | Homerus Swallowtail (0.0%) | .23 |
| A | 359 | 351 | NA | NA | Who ruled Jerusalem from 1099 to 1187 CE? | European Crusaders | .27 | 11830 | 15937 | Egyptians (24.5%) | Seljuq Turks (16.3%) | The Ottoman Empire (32.7%) | .23 |
| A | 359 | 362 | NA | NA | Name the title character of a popular rap-themed video game that was originally released on the Sony PlayStation in 1996. | PaRappa the Rapper | .24 | 12538 | 17977 | Ali G (12.2%) | Duke Nukem (32.7%) | MC Skat Kat (30.6%) | .21 |
| A | 359 | 372 | NA | NA | What was the first rap song to hit number one on the Billboard Hot 100 chart? | Ice Ice Baby | .22 | 10886 | 11508 | Bust a Move (12.2%) | U Can't Touch This (53.1%) | Wild Thing (12.2%) | .19 |
|  |  |  |  |  |  |  |  | MC  (response times) | | MC FOILS  (% of times selected as response) | | |  |
| Set | CR Rank | MC rank | T. et al. rank | N & N rank | Question | CORRECT ANSWER | Correct | Errors | Correct | Foil 1 | Foil 2 | Foil 3 | Difference (MC-CR) |
| A | 359 | 384 | NA | NA | Which British city was named Deva by the Romans? | Chester | .18 | 8691 | 7856 | Bath (42.9%) | Cambridge (30.6%) | Exeter (8.2%) | .15 |
| A | 359 | 390 | NA | NA | Who wrote the 1859 novel "Oblomov" whose lazy and daydreaming titular character satirizes the contemporary Russian nobility? | Ivan Goncharov | .16 | 14500 | 25349 | Anton Chekhov (20.4%) | Fyodor Dostoyevsky (16.3%) | Leo Tolstoy (46.9%) | .13 |
| A | 359 | 396 | NA | NA | The ice-cream cone originates from what American state?) | Missouri | .14 | 7340 | 11017 | California (6.1%) | Illinois (28.6%) | New York (51.0%) | .11 |
| A | 359 | 396 | NA | NA | What does a brandophile collect? | Cigar bands | .14 | 10544 | 10216 | Brandy (28.6%) | Logos (46.9%) | Wood (10.2%) | .11 |
| A | 359 | 396 | NA | NA | Which baseball player holds the record for most professional hits across all baseball leagues? | Ichiro Suzuki | .14 | 11502 | 19672 | Babe Ruth (30.6%) | Mickey Mantle (18.4%) | Ty Cobb (36.7%) | .11 |
| A | 359 | 396 | NA | NA | Who received the 1951 Nobel prize in physics? | Ernest Walton | .14 | 8224 | 16722 | Albert Einstein (59.2%) | Brian May (6.1%) | Francis Crick (20.4%) | .11 |
| A | 359 | 400 | NA | NA | What is the name for Earth on the Little Prince's planet? | Asteroid MU-330 | .12 | 12633 | 10047 | Asteroid MU-12 (16.3%) | Earth (44.9%) | Zeta Reticuli (26.5%) | .09 |
| A | 359 | 400 | NA | NA | What is the name given to the short tail of a rabbit? | Scut | .12 | 8151 | 12228 | Cottonball (55.1%) | Stub (20.4%) | Tag (12.2%) | .09 |
|  |  |  |  |  |  |  |  | MC  (response times) | | MC FOILS  (% of times selected as response) | | |  |
| Set | CR Rank | MC rank | T. et al. rank | N & N rank | Question | CORRECT ANSWER | Correct | Errors | Correct | Foil 1 | Foil 2 | Foil 3 | Difference (MC-CR) |
| D | 371 | 400 | 282 | 270 | What is the last name of the discoverer of the vaccination for smallpox? | Jenner | .12 | 7493 | 6831 | Hillman (10.2%) | Lederle (14.3%) | Pasteur (63.3%) | .09 |
| D | 371 | 351 | 292 | 287 | What is the last name of the poet who wrote the line "Into each life a little rain must fall"? | Longfellow | .27 | 9340 | 11420 | Emerson (20.4%) | Frost (26.5%) | Thoreau (26.5%) | .24 |
| B | 372 | 362 | NA | NA | What do you call the weapon used by Indians and gauchos of South America to entangle the legs of cattle and other animals? | Bola | .24 | 13333 | 12461 | Bolero (10.2%) | Lariat (24.5%) | Lasso (40.8%) | .23 |
| A | 384 | 202 | NA | NA | What school did Billy Bunter attend? | Greyfriars | .63 | 6641 | 10366 | Dotheboys Hall (16.3%) | Hailsham (10.2%) | Malory Towers (10.2%) | .62 |
| A | 384 | 254 | NA | NA | Who is the patron saint of Armenia? | St. Gregory | .51 | 9602 | 8356 | St. Andrew (16.3%) | St. David (12.2%) | St. George (20.4%) | .49 |
| A | 384 | 268 | NA | NA | What is the name for a curry made of spinach and cheese? | Palak Paneer | .47 | 12659 | 8175 | Aloo Gobhi (8.2%) | Papadum (18.4%) | Tikka (26.5%) | .45 |
| A | 384 | 274 | NA | NA | Who was Australia's first prime minister? | Edmund Barton | .45 | 7043 | 7403 | Alfred Deakin (18.4%) | Andrew Fisher (18.4%) | Arthur Phillip (18.4%) | .43 |
| A | 384 | 316 | NA | NA | What is caterpillar waste called? | Frass | .35 | 8392 | 12538 | Clods (26.5%) | Coprolites (30.6%) | Guano (8.2%) | .33 |
|  |  |  |  |  |  |  |  |  |  |  |  |  |  |
|  |  |  |  |  |  |  |  | MC  (response times) | | MC FOILS  (% of times selected as response) | | |  |
| Set | CR Rank | MC rank | T. et al. rank | N & N rank | Question | CORRECT ANSWER | Correct | Errors | Correct | Foil 1 | Foil 2 | Foil 3 | Difference (MC-CR) |
| A | 384 | 316 | NA | NA | What is the basic unit of currency for Myanmar? | Kyat | .35 | 6927 | 9931 | Dirham (16.3%) | Rupee (36.7%) | Tugrik (12.2%) | .33 |
| A | 384 | 327 | NA | NA | What Latin American author wrote in green ink? | Pablo Neruda | .33 | 13064 | 8621 | Carlos Fuentes (22.4%) | Isabel Allende (28.6%) | Julio Cortazar (16.3%) | .31 |
| A | 384 | 327 | NA | NA | Who is the Hindu god associated with rain? | Indra | .33 | 7735 | 9499 | Brahma (12.2%) | Shiva (22.4%) | Vishnu (32.7%) | .31 |
| A | 384 | 334 | NA | NA | Gwendolyn Brooks won a Pulitzer in 1950 for which book of poems? | Annie Allen | .31 | 12866 | 19680 | Boy Breaking Glass (26.5%) | Riot (16.3%) | The Ballad of Rudolph Reed (26.5%) | .29 |
| A | 384 | 343 | NA | NA | Who invented the aerosol spray can? | Erik Rotheim | .29 | 8725 | 8402 | Fritz Haber (26.5%) | Percy Lavon Julian (10.2%) | Wallace Carothers (34.7%) | .27 |
| A | 384 | 343 | NA | NA | Who is the only one of Milton's contemporaries to be mentioned by name in"Paradise Lost"? | Galileo | .29 | 10730 | 9738 | Francis Bacon (42.9%) | Johannes Vermeer (16.3%) | King Charles I (12.2%) | .27 |
| A | 384 | 362 | NA | NA | What type of Cubism incorporated pieces of newspaper? | Synthetic Cubism | .24 | 10251 | 8118 | Art Deco Cubism (49.0%) | Early Cubism (8.2%) | Existential Cubism (18.4%) | .23 |
| A | 384 | 362 | NA | NA | Who sang "Amityville House On The Hill"? | Lovebug Starski | .24 | 10228 | 13798 | Busy Bee Starski (8.2%) | DJ Kool Herc (14.3%) | Grandmaster Flash (53.1%) | .23 |
|  |  |  |  |  |  |  |  |  |  |  |  |  |  |
|  |  |  |  |  |  |  |  | MC  (response times) | | MC FOILS  (% of times selected as response) | | |  |
| Set | CR Rank | MC rank | T. et al. rank | N & N rank | Question | CORRECT ANSWER | Correct | Errors | Correct | Foil 1 | Foil 2 | Foil 3 | Difference (MC-CR) |
| A | 384 | 372 | NA | NA | Which German duo have sold over 85 million records? | Modern Talking | .22 | 10876 | 8932 | Fair Control (10.2%) | Le Click (20.4%) | Milli Vanilli (46.9%) | .21 |
| A | 384 | 384 | NA | NA | In which British national daily newspaper does Rupert The Bear appear? | The Daily Express | .18 | 10909 | 18990 | The Guardian (30.6%) | The Sun (18.4%) | The Sunday Telegraph (32.7%) | .17 |
| A | 384 | 384 | NA | NA | What is the tallest mountain in Iran? | Mount Damavand | .18 | 6681 | 9285 | Mount Sabalan (42.9%) | Mount Sahand (22.4%) | Mount Taftan (16.3%) | .17 |
| A | 384 | 384 | NA | NA | What was the name of William Wordsworth's sister? | Dorothy | .18 | 6260 | 7672 | Anne (34.7%) | Ellen (10.2%) | Mary (36.7%) | .17 |
| A | 384 | 384 | NA | NA | Which team won the first World Series in 1903? | Boston Americans | .18 | 7592 | 14570 | Chicago Cubs (63.3%) | Cleveland Naps (4.1%) | New York Highlanders (14.3%) | .17 |
| A | 384 | 390 | NA | NA | What television talent show did Mary Hopkin win? | Opportunity Knocks | .16 | 9450 | 14718 | American Idol (18.4%) | New Faces (16.3%) | Star Search (49.0%) | .15 |
| A | 384 | 406 | NA | NA | What is the name of the woman Romeo is infatuated with before he meets Juliet? | Rosaline | .10 | 9686 | 10794 | Ophelia (34.7%) | Rosalind (24.5%) | Rosamund (30.6%) | .08 |
| A | 384 | 406 | NA | NA | Which Suffolk town was the birthplace of Benjamin Britten? | Lowestoft | .10 | 8356 | 8075 | Ipswich (26.5%) | Southwold (26.5%) | Sudbury (36.7%) | .08 |
|  |  |  |  |  |  |  |  |  |  |  |  |  |  |
|  |  |  |  |  |  |  |  | MC  (response times) | | MC FOILS  (% of times selected as response) | | |  |
| Set | CR Rank | MC rank | T. et al. rank | N & N rank | Question | CORRECT ANSWER | Correct | Errors | Correct | Foil 1 | Foil 2 | Foil 3 | Difference (MC-CR) |
| A | 384 | 416 | NA | NA | What liqueur goes into making a 'Snowball' cocktail? | Advocaat | .06 | 9643 | 14139 | Chambord (18.4%) | Drambuie (32.7%) | Sambuca (42.9%) | .04 |
| A | 384 | 420 | NA | NA | Alan McGee signed Oasis to which label in 1993? | Creation | .04 | 8850 | 45500 | Atlantic (26.5%) | Polygram (32.7%) | Sony (36.7%) | .02 |
| D | 399 | 372 | 203 | 286 | What is the last name of the twenty-first U.S. President? | Arthur | .22 | 10463 | 12011 | Cleveland (26.5%) | Garfield (30.6%) | Hayes (20.4%) | .21 |
| D | 399 | 293 | 220 | 296 | What is the last name of the man who supposedly killed Jesse James? | Ford | .41 | 11052 | 10344 | Garrett (44.9%) | Hite (2.0%) | Younger (12.2%) | .39 |
| D | 399 | 378 | 229 | 297 | What is the last name of the First American author to win the Nobel Prize for Literature? | Lewis | .20 | 10072 | 13367 | Faulkner (22.4%) | Hemingway (49.0%) | O'Neill (8.2%) | .19 |
| D | 399 | 362 | 240 | 288 | What was the name of the nuclear submarine that sunk in the Atlantic in 1963? | Thresher | .24 | 8111 | 16773 | Nautilus (28.6%) | Seawolf (26.5%) | Triton (20.4%) | .23 |
| D | 399 | 351 | 281 | 269 | What is the name of the first movie to receive the Academy Award for Best Picture? | Wings | .27 | 9045 | 11310 | Alibi (2.0%) | All Quiet on the Western Front (69.4%) | Sunrise (2.0%) | .25 |
|  |  |  |  |  |  |  |  | MC  (response times) | | MC FOILS  (% of times selected as response) | | |  |
| Set | CR Rank | MC rank | T. et al. rank | N & N rank | Question | CORRECT ANSWER | Correct | Errors | Correct | Foil 1 | Foil 2 | Foil 3 | Difference (MC-CR) |
| D | 399 | 406 | 291 | 283 | What is the name of a number two wood in golf? | Brassie | .10 | 10175 | 11674 | Cleek (2.0%) | Driver (73.5%) | Iron (14.3%) | .09 |
| A | 412 | 282 | NA | NA | What is the first-person plural pronoun in Hindi? | Hum | .43 | 10903 | 9788 | Main (6.1%) | We (8.2%) | Wir (42.9%) | .43 |
| A | 412 | 316 | NA | NA | Who dubbed Australia "the lucky country"? | Donald Horne | .35 | 11683 | 9300 | George Johnston (34.7%) | Joan Lindsay (6.1%) | Thea Astley (24.5%) | .35 |
| A | 412 | 334 | NA | NA | Which Pulitzer Prize winner has won more than twice? | Robert Frost | .31 | 8793 | 12051 | Barbara W. Tuchman (14.3%) | Margaret Leech (6.1%) | William Faulkner (49.0%) | .31 |
| A | 412 | 343 | NA | NA | What was the first newspaper to utilize advertisements for revenue and the "penny paper" format? | The Sun | .29 | 12696 | 11912 | The Daily Courant (18.4%) | The New York Times (40.8%) | The Washington Post (12.2%) | .29 |
| A | 412 | 351 | NA | NA | What is the capital of Burundi? | Bujumbura | .27 | 16531 | 8314 | Kabezi (16.3%) | Muramvya (38.8%) | Ngozi (18.4%) | .27 |
| A | 412 | 351 | NA | NA | Which country won the first women's Olympic handball gold medal in 1976? | The Soviet Union | .27 | 13690 | 10067 | Hungary (20.4%) | Japan (30.6%) | United States (22.4%) | .27 |
| A | 412 | 362 | NA | NA | A sufferer from Boanthropy believes he is what? | An ox | .24 | 11541 | 12730 | A boat (10.2%) | A celebrity (18.4%) | A snake (46.9%) | .24 |
| A | 412 | 362 | NA | NA | Which animal has the widest hearing range? | Dolphin | .24 | 11153 | 10200 | Bat (49.0%) | Dog (22.4%) | Human being (4.1%) | .24 |
|  |  |  |  |  |  |  |  | MC  (response times) | | MC FOILS  (% of times selected as response) | | |  |
| Set | CR Rank | MC rank | T. et al. rank | N & N rank | Question | CORRECT ANSWER | Correct | Errors | Correct | Foil 1 | Foil 2 | Foil 3 | Difference (MC-CR) |
| A | 412 | 390 | NA | NA | What is the name of a dried legume? | Pulse | .16 | 9713 | 7858 | Nut (38.8%) | Pill (2.0%) | Pod (42.9%) | .16 |
| A | 412 | 390 | NA | NA | What was the name of Grotbags' pet in the TV show "Emu's World"? | Croc | .16 | 13196 | 6293 | Brat (16.3%) | Emu (42.9%) | Midnight (24.5%) | .16 |
| A | 412 | 396 | NA | NA | Who replaced Betty Boothroyd as "Speaker of the House of Commons"? | Michael Martin | .14 | 11631 | 11910 | Bernard Weatherill (38.8%) | George Thomas (24.5%) | John Bercow (22.4%) | .14 |
| A | 412 | 406 | NA | NA | Which mountain range runs along the south shore of the Caspian Sea? | Elburz | .10 | 8633 | 9258 | Caucasus (57.1%) | Talysh (8.2%) | Zagros (24.5%) | .10 |
| A | 412 | 412 | NA | NA | What is the meaning of the name Himalaya in Sanskrit? | Abode of snow | .08 | 11022 | 6067 | Majestic mountain (22.4%) | Peak of heaven (40.8%) | The high one (28.6%) | .08 |
| A | 412 | 416 | NA | NA | What was the first Arcade game ever released? | Computer Space | .06 | 7651 | 37786 | Pacman (22.4%) | Pong (67.3%) | Speed Race (4.1%) | .06 |
| A | 412 | 416 | NA | NA | Which branch of physics is particularly useful in designing bridges? | Statics | .06 | 10024 | 11003 | Mechanics (79.6%) | Quantum (10.2%) | Statistical (4.1%) | .06 |
| A | 412 | 420 | NA | NA | Who invented the Christmas Cracker? | Tom Smith | .04 | 9553 | 3034 | Charles Plimpton (36.7%) | Frank Hornby (26.5%) | Ole Kirk Christiansen (32.7%) | .04 |
|  |  |  |  |  |  |  |  |  |  |  |  |  |  |
|  |  |  |  |  |  |  |  | MC  (response times) | | MC FOILS  (% of times selected as response) | | |  |
| Set | CR Rank | MC rank | T. et al. rank | N & N rank | Question | CORRECT ANSWER | Correct | Errors | Correct | Foil 1 | Foil 2 | Foil 3 | Difference (MC-CR) |
| A | 412 | 421 | NA | NA | What was raced in the first competitive event at the Indianapolis Motor Speedway? | Balloons | .02 | 10291 | 201 | Bicycles (16.3%) | Cars (71.4%) | Horses (10.2%) | .02 |
| B | 412 | 316 | NA | NA | What do you call the accumulation of earth and stones carried and finally deposited by a glacier? | Moraine | .35 | 15983 | 11239 | Cirque (10.2%) | Esker (18.4%) | Tarn (36.7%) | .35 |
| D | 412 | 351 | 246 | 194 | What is the name of the baseball player with the highest lifetime batting average in the major leagues? | Cobb | .27 | 11743 | 12323 | Aaron (32.7%) | DiMaggio (22.4%) | Mays (18.4%) | .27 |
| D | 412 | 406 | 297 | 298 | What is the last name of the man who wrote the poem "It Couldn't Be Done"? | Guest | .10 | 7686 | 11249 | Millay (16.3%) | Stevens (20.4%) | Yeats (53.1%) | .10 |

*Notes*: CR = cued-recall; MC= multiple-choice; T et al. = Tauber et al. (2013); N & N = Nelson and Narens (1980), CE = commission errors; DR = don't remember; DK = don't know; OE = omission errors
